# Use of Guideline-Recommended Heart Failure Drugs in High-, Middle-, and Low-Income Countries: A Systematic Review and Meta-Analysis

**DOI:** 10.5334/gh.1355

**Published:** 2024-09-12

**Authors:** Gautam Satheesh, Rupasvi Dhurjati, Laura Alston, Fisaha Tesfay, Rashmi Pant, Ehete Bahiru, Claudia Bambs, Anubha Agarwal, Sanne A. E. Peters, Abdul Salam, Isabelle Johansson

**Affiliations:** 1 The George Institute for Global Health, Hyderabad, India; 2 Deakin Rural Health, School of Medicine, Faculty of Health, Deakin University, Warrnambool, Australia; 3 Global Centre for Preventive Health, Institute for Health Transformation, Deakin University, Geelong, Australia; 4 Department of Cardiology, University of Washington, Seattle, Washington, USA; 5 Department of Public Health, Advanced Center for Chronic Diseases (ACCDiS, Fondap 15130011) and Center for Cancer Prevention and Control (CECAN, Fondap 152220002), School of Medicine, Pontificia Universidad Católica de Chile, Santiago, Chile; 6 Cardiovascular Division, Washington University School of Medicine in St. Louis, St. Louis, Missouri, USA; 7 The George Institute for Global Health, University of New South Wales, Sydney, Australia; 8 Julius Center for Health Sciences and Primary Care, University Medical Center Utrecht, Utrecht, the Netherlands; 9 The George Institute for Global Health, School of Public Health, Imperial College London, London, United Kingdom; 10 Prasanna School of Public Health, Manipal Academy of Higher Education, Manipal, India; 11 Division of Cardiology, Department of Medicine K2, Karolinska University Hospital Solna, Karolinska Institutet, Stockholm, Sweden; 12 Population Health Research Institute, McMaster University, Hamilton, Canada

**Keywords:** guideline-directed medical therapy, heart failure, country income level, population, HFrEF

## Abstract

Optimal use of guideline-directed medical therapy (GDMT) can prevent hospitalization and mortality among patients with heart failure (HF). We aimed to assess the prevalence of GDMT use for HF across geographic regions and country-income levels. We systematically reviewed observational studies (published between January 2010 and October 2020) involving patients with HF with reduced ejection fraction. We conducted random-effects meta-analyses to obtain summary estimates. We included 334 studies comprising 1,507,849 patients (31% female). The majority (82%) of studies were from high-income countries, with Europe (45%) and the Americas (33%) being the most represented regions, and Africa (1%) being the least. Overall prevalence of GDMT use was 80% (95% CI 78%–81%) for β-blockers, 82% (80%–83%) for renin–angiotensin-system inhibitors, and 41% (39%–43%) for mineralocorticoid receptor antagonists. We observed an exponential increase in GDMT use over time after adjusting for country-income levels (*p* < 0.0001), but significant gaps persist in low- and middle-income countries. Multi-level interventions are needed to address health-system, provider, and patient-level barriers to GDMT use.

## Introduction

Heart failure (HF) is a major health concern affecting over 64 million individuals globally and causing substantial individual suffering as well as escalating healthcare and societal costs (1,2,3,4,5). It is among the leading causes of hospitalization and death, especially in low- and middle-income countries (LMICs; 1,2), due to aging populations, treatment advances for myocardial infarction, and the epidemiological transition toward non-communicable diseases. The prevalence of HF, and the resultant economic burden on health systems, is expected to rise in the coming decades (3,6). Major advances in the management of HF over the last decades (especially HF with reduced ejection fraction [HFrEF]) have led to improvements in outcomes for patients with HF (6,7). Recent evidence has shown marked differences in outcomes for patients with HF globally (2,8,9,10). These differences may partly be explained by the availability and utilization of guideline-directed medical therapy (GDMT) and other cardiac interventions across the world.

It is well established that GDMT for HFrEF reduces the risk of hospitalization and mortality (4,11,12). Gaps between recommended care and clinical practice have been highlighted in several studies, including from Europe (13,14), the United States (15,16), Asia, and Africa (12,17,18). A systematic review and meta-analysis by Callender et al. in 2014 found substantial variation in the management of HF across LMICs (2). The World Heart Federation (WHF) HF Roadmap presents the Ideal Heart Failure Continuum of Care Pathway. It identifies potential roadblocks and solutions for improving HF care and outcomes. Gaps in the adoption and use of GDMT were identified as one of the areas requiring the most urgent action (11). Further research on this topic is important to identify areas needing interventions to optimize the use of GDMT to improve HF care and outcomes worldwide.

In this systematic review and meta-analysis, we assessed the prevalence of the use of GDMT among patients with HFrEF and compared it across regions grouped by country and income.

## Methods

The protocol for this systematic review and meta-analysis was registered in the International Prospective Register of Systematic Reviews (PROSPERO [CRD42020210165]). This manuscript follows the Preferred Reporting Items for Systematic Reviews and Meta-Analyses (PRISMA) 2020 guidelines (19).

### Data sources

We developed a systematic search strategy for retrieving relevant studies and consulted a university librarian for its refinement. We searched MEDLINE, Global Health, and CINAHL from inception to October 5, 2020, using database-specific systematic search strategies containing both keywords and controlled vocabulary (Tables S1–S3). In addition, we screened the reference lists of a previous systematic review (2).

### Study selection

We included observational studies (including registry-based studies): cohort (retrospective or prospective) and cross-sectional studies, published in English, between January 1, 2010 and October 5, 2020. Included studies had to have at least 100 patients with confirmed HFrEF or documented left ventricular dysfunction without overt HF and have reported the use of at least one GDMT for HFrEF (Table 1; 12,20).

**Table 1:** Guideline-directed medical therapy for heart failure with reduced ejection fraction.


LIST OF GUIDELINE-DIRECTED MEDICAL THERAPY FOR HEART FAILURE WITH REDUCED EJECTION FRACTION

**Primary**

1. β-blockers [BBs]

2. Renin–angiotensin system [RAS] inhibitors

a. Angiotensin converting enzyme inhibitors [ACEis]

b. Angiotensin receptor II antagonists [ARBs]

3. Mineralocorticoid receptor antagonists [MRAs]*

4. Diuretics (loop- or thiazides)

**Secondary**

5. Digoxin

6. Ivabradine*

7. Angiotensin receptor neprilysin inhibitors [ARNIs]**

8. Hydralazine/nitrate

9. Sodium glucose co-transporter 2 inhibitors [SGLT-2is]***


*Guideline-recommended from 2012. **Guideline-recommended from 2016. ***Guideline-recommended from 2021.

HFrEF definitions were those used in the individual included studies. If HFrEF was not defined in a study, a left ventricular ejection fraction (LVEF) cut-off of <50% was used to cover different definitions of HFrEF throughout the period of included studies. Studies were included irrespective of whether the assessment of HFrEF drug use was among the main study objectives. Studies with broader eligibility criteria, where patients with HFrEF accounted for a subgroup of the total study population, were also included if they reported drug use for the sub-group of HFrEF patients. Studies were excluded if they were duplicates; that is, if the patient population was selected from the same administrative database/registry and covered the identical time span and location. For some studies, the decision to include them was made by prioritizing (in the given order) a longer recruitment period, larger sample size, and the available information on GDMT use as further specified in the supplements. We also excluded studies where data were pooled from more than one study or where only pooled data were presented, as including such studies in our meta-analyses may introduce the risk of including duplicate populations. Furthermore, we excluded studies wherein GDMT use was an inclusion criterion, that is, studies that excluded patients who were not on GDMT.

At least two reviewers (IJ, RD, GS, LA, and FT) and in duplicate screened titles and abstracts and reviewed the full text to assess study eligibility for inclusion. Disagreements were resolved by discussion between at least two reviewers, and a third reviewer was consulted if necessary.

### Outcomes

The primary outcome was the percentage of patients prescribed or using each class of first-line GDMT for HFrEF (BB, RASi, MRA, and diuretics). Secondary outcomes included (1) time trends in the prevalence of GDMT use and (2) prevalence of other classes of GDMT (e.g., digoxin, ivabradine, etc.) for HFrEF. The prevalence of GDMT use was defined as the percentage of HFrEF patients using GDMT drugs based on (1) hospital discharge report, (2) pharmacy refill records, (3) self-reported use, or (4) physical verification of drugs, according to how each study reported GDMT data.

### Data extraction

Data from each included study were collected by two reviewers independently and in duplicate. Conflicts in the data collected between the reviewers were resolved through discussion and consensus, and in case of disagreement, a third reviewer was consulted. The collected data included characteristics of the study, participants, and information on the pre-specified HF drugs. For studies among inpatient populations, we preferentially collected baseline drug use data unless it was unavailable or clearly stated that HF was newly diagnosed, in which case discharge drug use was recorded. For multi-country studies, country-specific data were extracted where possible, otherwise, we contacted the first author to obtain country-specific data.

### Data analysis

We used study-specific percentages of GDMT use as reported or, when presented as sub-groups, we calculated the average percentage weighted by sample size. We conducted random-effects meta-analyses to obtain summary estimate of prevalence of GDMT with corresponding 95% confidence intervals (CIs). We assessed between-study heterogeneity quantitatively using Higgin’s *I*^2^ statistic (21). Pre-specified subgroup analyses were performed according to country income level [based on World Bank 2021 (22) country income classification] and geographic region [based on World Health Organization (23) classification]. Summary estimates were also obtained for subgroups of studies in which index data collection occurred in the last 10 and 5 years. Studies encompassing countries from multiple WHO regions or income groups without country-specific data were included in the systematic review but excluded from the meta-analyses. In a sensitivity analysis, we explored the impact of whether the study population was recruited from a non-acute or acute setting.

We assessed whether the recruitment time period affected the GDMT prevalence using random-effects meta-regression in separate analyses keeping the prevalence of use of each drug as a dependent variable and time period as a primary independent variable adjusting for country income levels as independent covariates. In sensitivity analyses, using bivariable and multivariable meta-regression models, we also included summary-level data for age (mean/median), sex (% female), diagnosis of hypertension, and ischemic heart disease as covariables. The output is presented as bubble plots where bubble sizes are inverse-variance weighted. The effect estimate for the strength of association is expressed as percentage change per year and 95% CI. *R*^2^-values were used in the adjusted meta-regression model to indicate the variability explained by the time period. A two-sided *p*-value <0.05 was considered statistically significant for all analyses. Comprehensive Meta-Analysis (CMA; Version 3; Borenstein et al., 2005) and Stata (version 17.0, StataCorp LLC, College Station, TX, USA) were used for analyses.

### Assessment of risk of bias in included studies

Two reviewers independently and in duplicate assessed the methodological quality of the included studies, using an adapted version of the Joanna Briggs Institute (JBI) Critical Appraisal Checklist for Analytical Cross-Sectional Studies (https://synthesismanual.jbi.global). The JBI checklist form consists of eight questions, and with response options for each question being ‘yes’, ‘no’, ‘unclear’, and ‘not applicable’. Based on the responses to these eight questions, studies were graded on a scale of 1–8 and categorized into low (grade >5), intermediate (grade = 5), and high (grade <5) risk of bias. Differences in ratings between the reviewers were discussed, and any disagreements were resolved through consensus.

## Results

### Study distribution and characteristics

**Figure 1:**
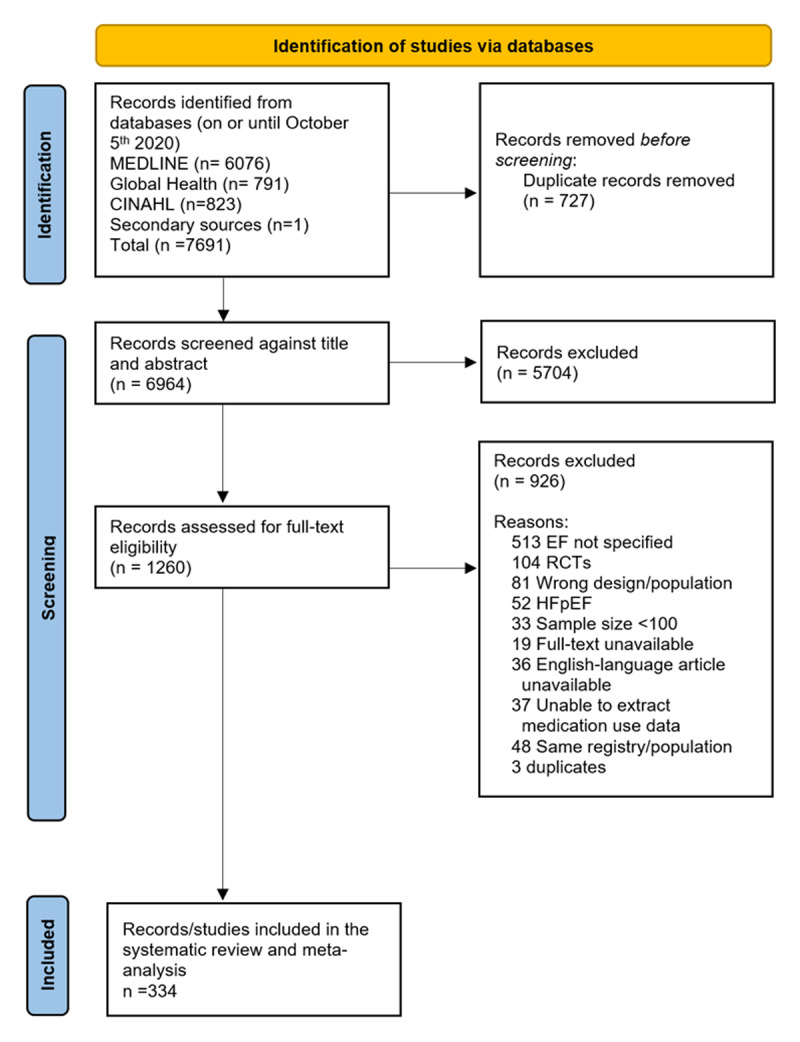
PRISMA chart.

**Figure 2:**
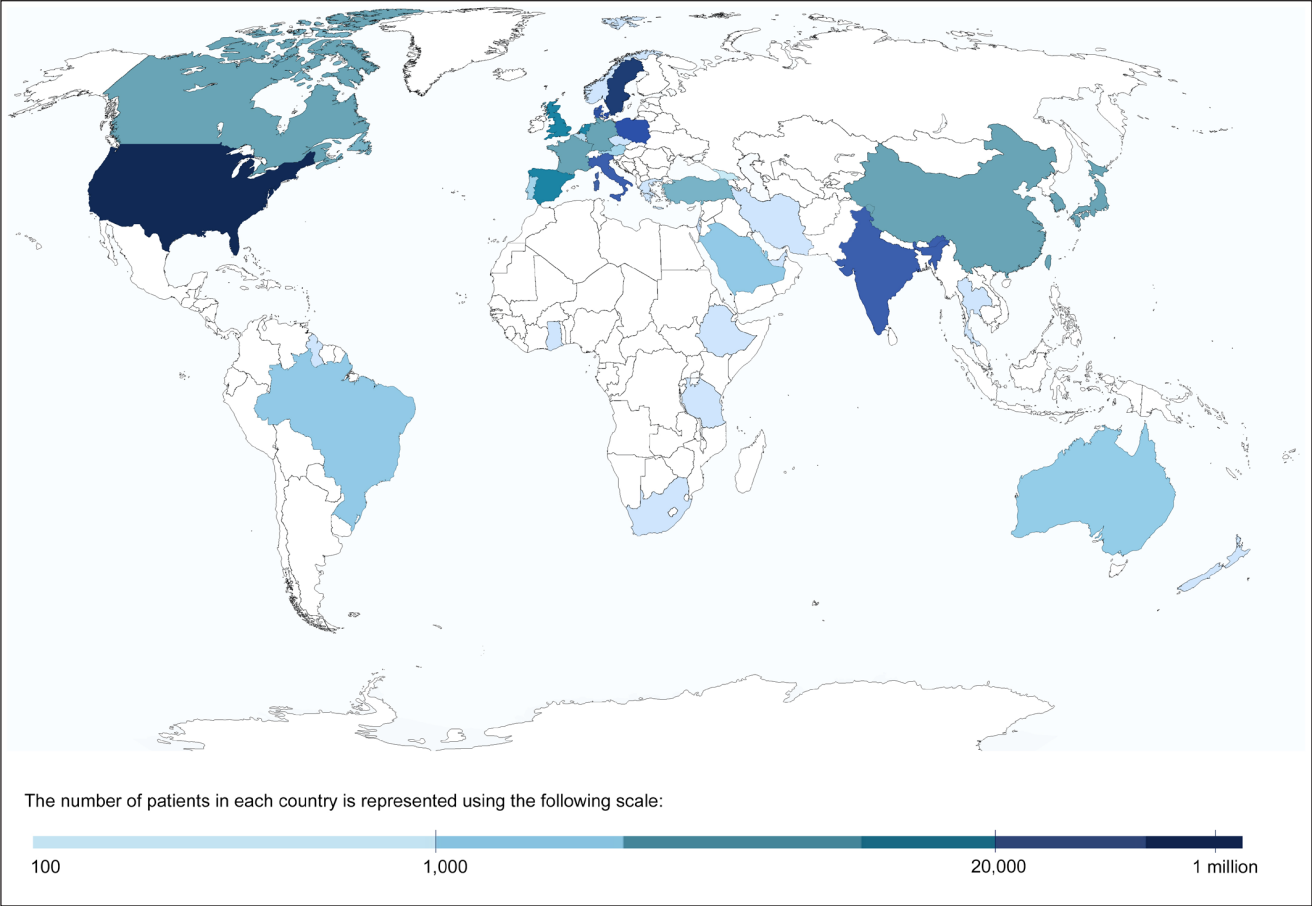
World map with countries and patients represented in the review.^1^ ^1^The actual number of patients from each country can be found in Tables S8–S11.

We identified 7692 published records, of which 334 studies (Figure 1) across 6 geographic regions and 4 country income levels met the inclusion criteria (Tables S4 and S5). In addition, we obtained unpublished data from two studies (24,25). The number of studies and participants by geographic region are summarized in Figure 2. There were 272 (82%) studies from HICs and 45 (13%) from LMICs [37 (11%) from upper MICs, 6 (2%) from lower MICs, and 2 (1%) from LICs]. The most represented regions were Europe (*n* = 151; 45%), followed by the Americas (*n* = 111; 33%), and Africa being the least represented region (*n* = 5; 1%). Of the included studies, 18% were based on patients from an acute setting and the remaining 82% were from a non-acute setting. In total, there were 1,507,849 patients with HF. Echocardiography was the most common method for establishing a diagnosis of HF and to determine LVEF. The risk of bias was low in 37% (*N* = 124) studies, intermediate in 58% (*N* = 195), and high in 5% (*N* = 16) of the studies (Table S14). For the overall prevalence of use of each GDMT class, excluding studies with high-risk bias did not change the estimates significantly.

Baseline characteristics of the patients at the study level by geographic region are presented in Table 2. Women accounted for 31% of patients (56% in LICs, 29% in lower MICs, 33% in upper MICs, and 30% in HICs). The overall mean age was 65.7 years and differed across country income groups; the lowest in LICs (53 years), followed by lower-MICs (58 years), upper-MICs (63 years), and HICs (67 years). Overall, the most reported comorbidity was hypertension (60%) followed by ischemic heart disease (IHD; 53%). This pattern was similar in HICs and MICs while IHD was more common than hypertension in LICs.

**Table 2:** Study-level patient’s demographics and comorbidities by income setting and geographic region.


	STUDIES (PATIENTS)			FEMALE (%)	MEAN OR MEDIAN AGE	MEAN OR MEDIAN LVEF	HTN (%)	DM (%)	IHD/CAD (%)	AF (%)	CKD (%)	COPD (%)	CVA (%)

	OVERALL	ACUTE	NON-ACUTE										

**Total studies (population)**	334 (1,507,849)	60 (278,210)	274 (1,229,639)	30.5	65.7	29.4	59.4	33.7	53.2	29.2	26.9	19.6	11.6

**Income status**													

High-income	272 (1,399,151)	47 (258,165)	226 (1,141,367)	30.3	66.5	28.9	60.8	34.3	53.9	30.1	28.2	21.2	11.8

LMIC	45 (45,781)	9 (8686)	36 (37,095)	33.1	61.8	32.0	52.0	29.0	47.9	23.6	21.8	11.5	12.1

*Upper-middle income*	37 (21,851)	7 (7929)	30 (13,922)	32.6	62.9	31.6	54.7	29.6	50.8	26.9	24.3	11.8	14.7

*Lower-middle income*	6 (23,475)	1 (610)	6 (22865)	28.7	58.3	34.0	50.2	36.6	45.7	10.8	16.2	9.1	4.2

*Low-income*	2 (455)	1 (147)	1 (308)	55.5	52.6	40.4	27.4	9.1	33.1	21.3	5.5	–	–

**WHO regions**													

Africa	5 (1676)	2 (757)	3 (919)	51.5	55.1	36.4	40.6	13.4	21.8	19.0	10.0	2.8	6.5

Americas	111 (1,091,008)	24 (183,731)	87 (907,277)	34.4	65.6	27.5	66.7	38.6	55.6	30.2	26.4	27.4	12.6

E. Mediterranean	6 (5063)	2 (787)	4 (4276)	21.9	59.5	32.4	62.9	60.1	61.5	12.5	14.3	11.2	6.8

Europe	151 (293,235)	15 (67,263)	141 (237,659)	27.2	66.5	30.1	57.6	29.1	54.2	30.1	32.2	17.6	11.0

South-East Asia	10 (31,093)	3 (5401)	7 (25,692)	33.2	63.6	28.8	48.9	35.8	45.2	15.9	15.2	8.0	6.9

Western Pacific	32 (22,147)	11 (9613)	21 (12,534)	31.9	67.0	31.4	50.0	32.2	47.0	31.3	30.8	11.1	14.7


Studies involving data from multiple income groups not included. LMIC: low- and middle-income; AF: atrial fibrillation; LVEF: left ventricular ejection fraction; HTN: hypertension; IHD/CAD: ischemic heart disease/coronary artery disease; CKD: chronic kidney disease; COPD: chronic obstructive pulmonary disease; CVA: cardiovascular accident; DM: diabetes mellitus; E. Mediterranean: Eastern Mediterranean.

### Prevalence of use of GDMT For HFrEF

#### β-blockers

**Figure 3:**
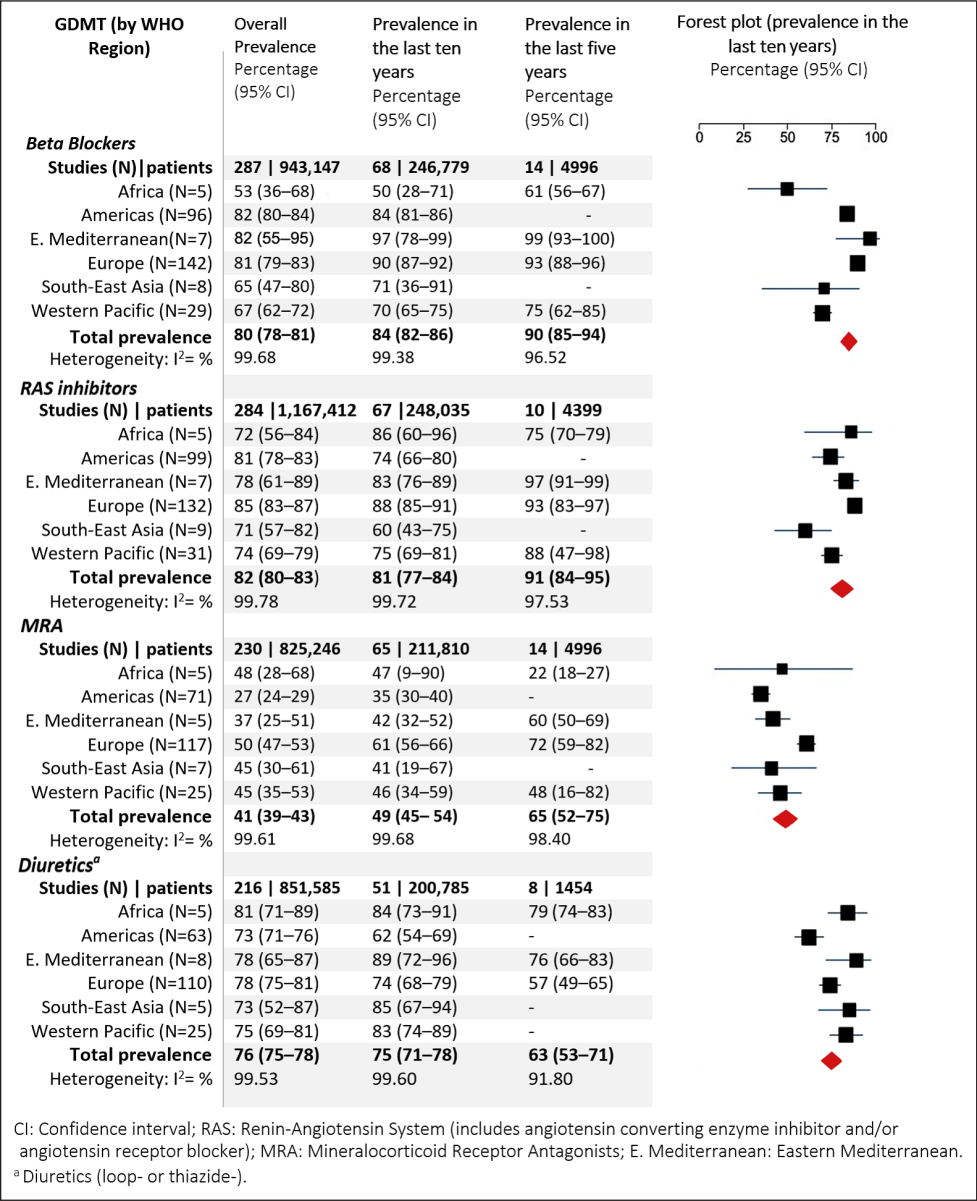
Prevalence of use of GDMT by WHO regions.

**Figure 4:**
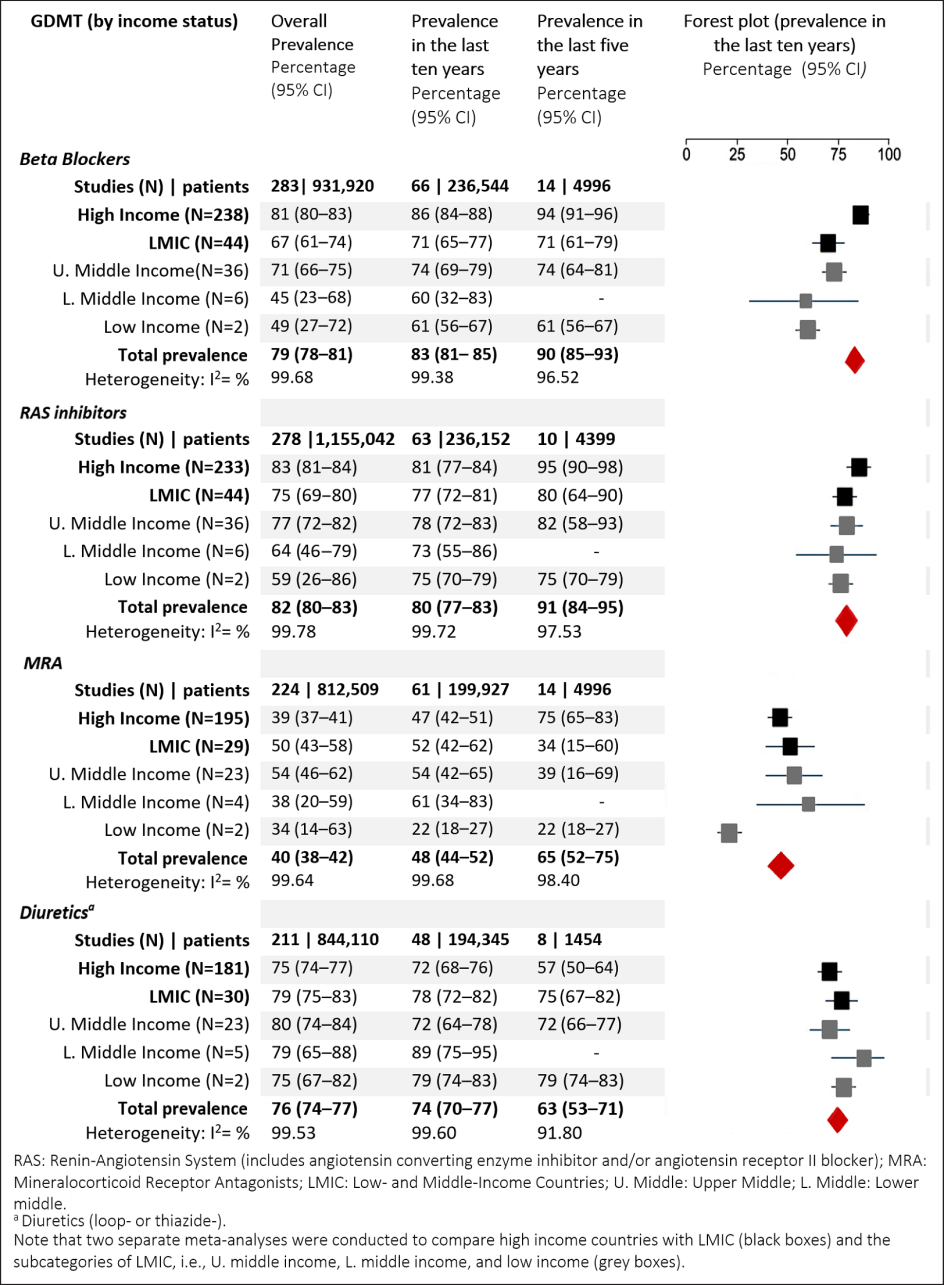
Prevalence of GDMT use by income status.

The overall prevalence of use of BBs was 80% (95% CI 78%–81%; Figure 3). Prevalence of use was the highest in the Americas (82% [80%–84%]) and the lowest in Africa (53% [36%–68%]). Prevalence of use was higher in HICs (81% [80%–83%]) compared to LMICs (67% [61%–74%], Figure 4). Prevalence of use was higher in non-acute (81% [79%–82%]) compared with the acute setting (73% [68%–78%]), Tables S6 and S7).

#### Renin–angiotensin system inhibitors

The overall prevalence of use of RAS inhibitors was 82% (80%–83%; Figure 3). It was the highest in the Americas (81% [78%–83%]) and Europe (85% [83%–87%]) and slightly lower in the other regions (69% [50%–84%] to 78% [61%–89%]). Prevalence of use in HICs was 83% (81%–84%) and 75% (69%–80%) in LMICs, and it was higher in the non-acute (83% [81%–85%] compared with the acute setting (75% [72%–78%], Tables S6 and S7).

#### Mineralocorticoid receptor antagonists

The overall prevalence of use of MRA was 41% (39%–43%). It was the highest in Europe (50% [47%–53%]) and Africa (48% [28%–68%]) and the lowest in the Americas 27% (24%–29%). Prevalence of use was lower in HICs (39% [37%–41%] than in LMICs (50% [43%–58%]) and was marginally higher in the non-acute (42% [39%–44%] compared with the acute setting (40% [33%–47%]), Tables S6 and S7.

#### Diuretics

The overall prevalence of use of diuretics was 76% (75%–78%). It was the highest in Africa (81% [71%–89%]), and the lowest in the Americas (73% [71%–76%]), South-East Asia (73% [52%–87%]. Prevalence of diuretic use was lower in HICs 75% [74%–77%] compared to LMICs (79% [75%–83%]) and was lower in the non-acute (75% [74%–77%] compared with the acute setting (82% [76%–87%], Tables S6 and S7.

Country-wise prevalence of use of GDMT is reported in Tables S8–S11.

### Association of time and comorbid conditions on prevalence of use of GDMT for HFrEF

**Figure 5:**
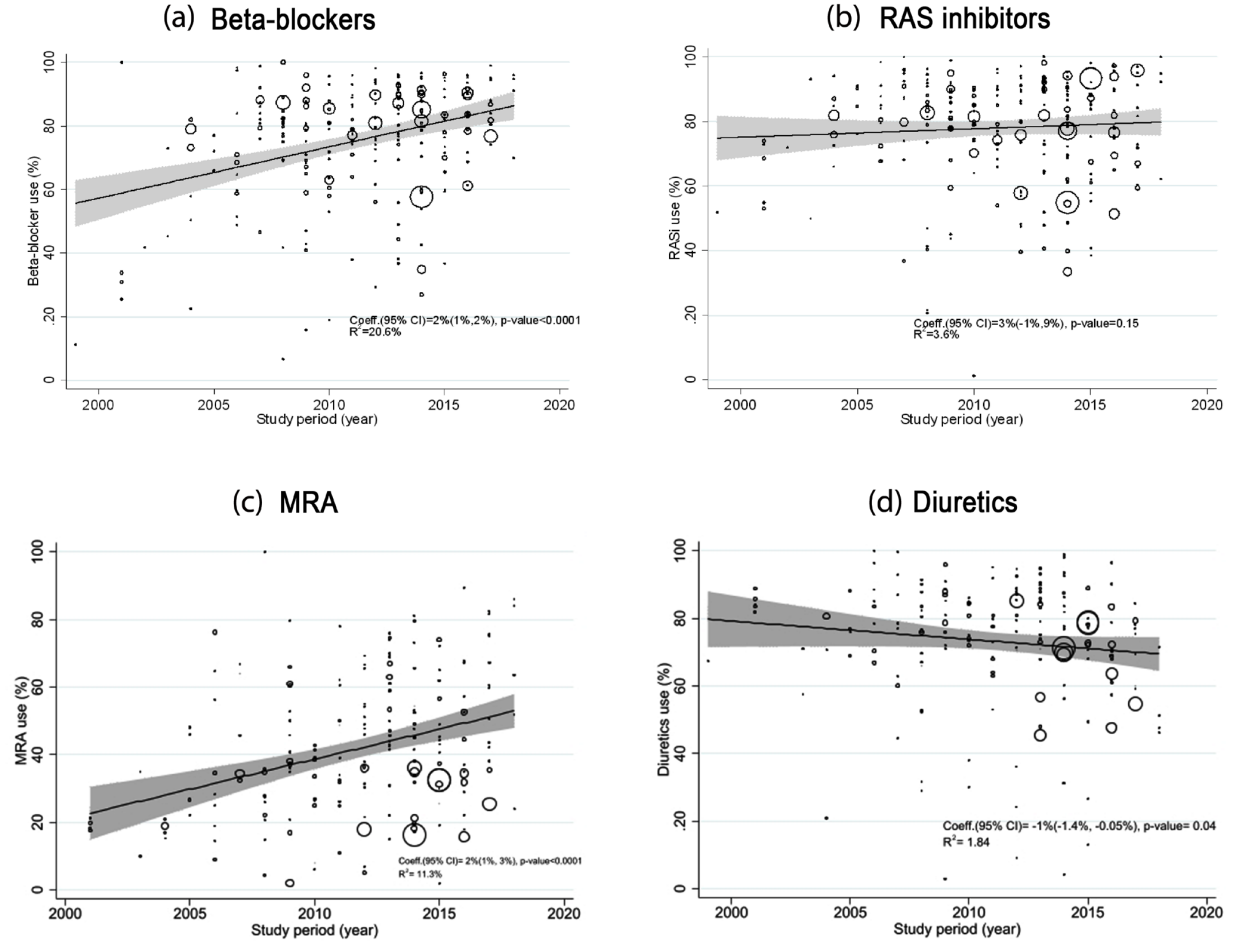
Meta-regression bubble plots for the association between study-year^1^ and prevalence of use of **(a)** β-blockers; **(b)** Renin–angiotensin system (RAS) inhibitors; **(c)** mineralocorticoid receptor antagonists (MRAs) and **(d)** diuretics use. Effect estimates and 95% confidence intervals (CI) are adjusted for country income level. ^1^Study-year was defined as end-date of data collection.

Overall, the prevalence of use showed an increasing trend in the last 10 years for β-blockers, RAS inhibitors, and MRAs, while it was decreasing for diuretics (Figures 3 and 4). After adjusting for country income level, the meta-regression analysis indicated that β-blocker use increased by an average of 2% (95% CI 1%–2%, *p* < 0.0001) per year and average MRA use increased by 2% (95% CI 1%–3%, *p* < 0.0001; Figure 5). Average diuretics use decreased by 1% (95% CI –1% to –4%, *p* = 0.04) and average RAS inhibitor use did not change significantly; 1% (95% CI –1% to 9%, *p* = 0.15) per year. The between-study heterogeneity in prevalence of use was explained by study period and country income level to some extent for β-blockers (adjusted *R*^2^ = 21%) and MRA (adjusted *R*^2^ = 11%), and not for RAS inhibitor use (adjusted *R*^2^ = 3.6%) and diuretic use (adjusted *R*^2^ = 1.8%).

Study-level age, sex, and presence of hypertension or IHD did not materially determine the treatment prevalence (Tables S15 and S16).

### Prevalence of use of other GDMT for HFrEF

Results of the use of digoxin, ivabradine, ARNI, hydralazine/nitrate, and SGLT 2 inhibitors are presented in Tables S12 and S13. Prevalence of use of digoxin was 25% (95% CI 24–27%; *I*^2^ 99.3%), and it was lower in the last 10 and further lower in the last 5 years. The overall as well as 10- and 5-year prevalence of digoxin use was higher in LMICs than HICs.

## Discussion

### Summary of findings

This systematic review and meta-analysis of 334 observational studies including 1.5 million patients estimated the global prevalence of use of GDMT for HFrEF. The prevalence of GDMT use was high for BBs (80%) and RAS inhibitors (82%), and relatively low for MRAs (41%), and increased over time, however with considerable variations across geographic regions and income groups. Between-study heterogeneity in global use was explained, to some extent, by the study period and country income level for BBs and MRAs but not for RAS inhibitors. Use of BBs and RAS inhibitors were higher, and MRAs were lower, in HICs compared to LMICs. Further, the prevalence of use was higher for BBs, RAS inhibitors, and MRAs in non-acute compared to acute settings.

The use of diuretics was high, and the use decreased over the last 10 and 5 years. The increasing use of BBs, RAS inhibitors, and MRAs over the years are likely attributable to the accumulation of clear evidence of their benefits, consequent evolution of stronger recommendations in guidelines, and improved availability.

### Findings in the context of other relevant evidence

Compared to the estimates reported by Callender et al. in 2014 (2), for LMICs, our systematic review estimated a higher prevalence of use for BBs, RAS inhibitors, and MRAs overall, and this observation was more pronounced in studies published in the last 10 years. This is expected, considering our findings of an increasing prevalence of GDMT use over time and that their meta-analysis covered an earlier time-period (studies published between 1995 and 2014). We report an age distribution (mean age: 66 years, ranging from 53 in LICs to 67 in HICs), which is consistent with that reported by Callender et al (mean age: 63 years. We only included studies where patients had LVEF <50%, which is a clear evidence-based indication for BB, RAS inhibitors, and MRA use, while Callender et al., included HFpEF patients (LVEF ≥50%) as well. They still observed considerable differences in GDMT use between different LMIC regions and a suboptimal use overall (2). Our meta-analyses provide optimism in that the treatment prevalence has somewhat improved over time although considerable differences remain between LMIC regions. Moreover, we add comparable data from approximately 230 HICs and show further inequalities in the prevalence of GDMT use between regions and income levels. In line with our findings, the REPORT HF study of 8669 patients hospitalized for acute HF between 2014 and 2017 in 44 countries, found considerable differences between HICs and LMICs 6 months after discharge (RAS inhibitors [71% vs. 57%], BBs [84% vs. 52%], and MRAs [59% vs. 45%] (26). The G-CHF study of 23,000 HF patients (majority outpatients) recruited from 40 countries between 2014 and 2017, has so far reported country income- and region-specific data, but not HF phenotype-specific data. Their findings suggest a higher prevalence of use of at least BBs in HICs compared to LICs, however phenotype-specific analyses may provide further insights (27,28).

INTER-CHF enrolled 5823 HF patients from 16 LMICs across Africa, Asia, and the Middle East between 2012 and 2014 (8). Compared to our observations, they reported lower estimates for BBs (67%) and RAS inhibitors (74%) but higher for MRAs (48%), which may partly be explained by 25% of the INTER-CHF population having HFpEF. Similar to our observations however, the use of BBs was the lowest in Africa and Asia (48% and 61% vs. 73% in South America and 86% in the Middle East), while RAS inhibitors and MRA use was the lowest in Asia and much higher in Africa, Middle East, and South America (29). Further, regional data from the ASIAN-HF, comprising 5276 HFrEF patients from 11 countries recruited between 2012 and 2015, found an overall prevalence of use of 77% for RAS inhibitors, 79% for BBs, and 58% for MRAs, which is higher than our findings for the corresponding WHO Regions, South-East Asia, and Western Pacific (71% and 70% respectively for BBs, 60% and 75% respectively for RAS inhibitors, and 41% and 46% respectively for MRAs) for studies published in the last 10 years (30). The ASIAN-HF also observed differences in GDMT uptake between HICs and lower income levels. Of note, China had the lowest uptake of RAS inhibitors but the highest uptake of MRAs, presumably driven by the low cost of spironolactone in China (30). From Europe, the BIOSTAT-CHF study of 2516 HFrEF patients enrolled from 11 European countries between 2010 and 2012 reported marked differences in the use of GDMT across Northern, Central, and Mediterranean areas. Patients from Central European centers in general received more RAS inhibitors, BBs, and MRAs compared to those from Northern European and Mediterranean centers (31). Similarly, although the ESC-HF-Long term registry of 12,440 patients from 21 European and Mediterranean countries recruited between 2011 and 2013 reported a high frequency of prescription of GDMT for HF patients, substantial between-region differences with a much lower use of HFrEF GDMT were reported in Northern Africa than other ESC regions (14). For South and Central America, the data from the first 1000 patients recruited in the American Registry of Ambulatory or acutely decompensated heart failure (AMERICCAASS) Registry, which was published in November 2023, report slightly lower prevalence of BB (71%) and RASi (52%) use and higher prevalence of MRA (57%), use, compared with our estimates (32). Similar estimates were reported by the G-CHF for BB (80% among women and 88% among men) and RASi (72% among women and 69% among men; 27). Further, the Sub-Saharan Africa Survey of Heart Failure (THESUS-HF) registry involves patients hospitalized with acute HF in 9 African countries. We report a higher prevalence of BB use (53% vs. 25%), similar for diuretics (76% vs. 80%) and RASi (72% vs. 87%), and a much lower prevalence for MRA (48% vs. 80%) use for the African region (33). It should be noted that the uptake of ARNI and SGLT2i is expected to have increased, especially among the HICs, since the end date of our search, given the stronger guideline recommendations and adoption. For instance, the G-CHF registry that recruited patients between 2016 and 2020 reported the use of ARNI to be 12% among men and 8% among women, which is similar to our estimate of 8% (27). However, the use of ARNI (25%) as well as SGLT2i (31%) reported by the AMERICCAASSS registry (2022–2023) was markedly higher (32). Another study, based on data from the Swedish HF registry, reported an increase in ARNI use from 8% in 2017 to 27% in 2021 (34). Nonetheless, there appears to be minimal improvement in LMICs; according to the prospective Cardiology Society of India-Kerala Acute Heart Failure Registry (CSI-KHFR), a very low proportion (2%) of patients with HFrEF used ARNI (35).

From the limited data available from LMICs, it is clear that the uptake of GDMT for HFrEF in LMICs, especially in Africa and parts of Asia, remains very suboptimal. This continues to be concerning and probably part of the reason for the large differences in outcomes for HFrEF patients across different regions and income levels. The underlying reasons influencing prescription patterns are likely multifactorial. Previous studies reported several factors to be associated with a lesser likelihood of GDMT use at the target dose, such as female sex, absence of health insurance, living in LMICs, or no scheduled follow-up in 6 months (26). Underuse of GDMT among women has also been shown in other studies (36), an important observation given the overrepresentation of young women in HFrEF cohorts especially from African countries, relative to HFrEF patients from other regions (driven mainly by rheumatic heart disease and anemia; 29,37). The higher prevalence of MRAs in LMICs compared with many HICs has been observed previously and is likely driven by a lower cost and availability.

### Implication for clinical practice and policy

HF is a significant health condition with high morbidity and mortality and substantial impact on quality of life and costs for individuals and societies around the globe. Around half of HF patients have HFrEF and for them guidelines recommend multidrug concurrent treatment with RAS inhibitors/ARNI, BBs, MRA, SGLT2i, and, if needed, diuretics to reduce mortality and disease progression. The Disease Control Priorities Project has recommended these drugs as the highest priority health system interventions (38). Yet, the use of GDMT is sub-optimal even in HICs (14,26). Despite younger age and fewer comorbidities, HFrEF patients in LMICs experience a higher risk of mortality compared to those in HICs, yet the prevalence of use of GDMT in LMICs is lower than HICs. This could be mainly due to the lack of recognition of GDMT as essential medicines (39), unavailability and unaffordability of GDMT for a large proportion of individuals, lack of medical specialists and programs to treat HF, and low patient literacy (11,40). Therefore, multi-level interventions are needed to address these health-system, healthcare provider, and patient-level barriers to improve the use of GDMT for better outcomes among patients with HFrEF. For instance, quality-of-care improvement initiatives have been shown to improve the use of GDMT (41). Health insurance, patient education, and HF awareness initiatives particularly in LMICs could significantly improve the use of GDMT. More data is needed for several LMICs to appreciate the magnitude of the problem, and the various registries of HF patients will be valuable in guiding future direction.

### Strengths and limitations

To our knowledge, this is the largest systematic review to date of the prevalence of use of GDMT across geographic regions, income groups, and individual countries. For 12 studies that did not report country-specific data, we contacted authors to obtain it with some success (17%). We excluded studies which did not clearly report if the patient population included had HFrEF, which yielded accurate estimates of the prevalence of use of GDMT among patients with HFrEF. Unfortunately, we could not collect data on the administered dose which may have resulted in overestimation of appropriate use of GDMT. In addition, although combination therapy is recommended for most cases of HFrEF, assessing this was outside the scope of the current review. Analyzing data based on the data collection period in the included studies enabled us to assess the prevalence of use of GDMT over time, particularly in recent years which is important for policy decisions. There were substantially fewer studies from LIC and lower MICs and for some drugs contributing to the analysis of the prevalence of use of GDMT in the last 5 years, therefore the trends should be interpreted with caution. Although sensitivity analyses showed no association on treatment prevalence of GDMT by the presence of hypertension or IHD, indication bias cannot be completely ruled out. Our definition of prevalence of use of GDMT was broad and included hospital and pharmacy records, discharge reports, self-reported use, and physical verification and is therefore a surrogate to actual use, which could have resulted in overestimation of prevalence of use. Next, determining the exact causes for poor uptake of GDMT was beyond the scope of the current review, given that the present meta-analysis is based on aggregate data and not individual participant data. Finally, there was high heterogeneity across studies which could be attributed to variation in study period and study population.

## Conclusion

Our systematic review and meta-analysis have shown that the prevalence of use of GDMT for HFrEF has been increasing over time. The prevalence was rather high for BBs and RAS inhibitors, especially in high-income countries. However, large gaps still exist, particularly in LMICs, where GDMT uptake for HFrEF is largely under-studied compared to HMICs. These persistent gaps need to be further understood. Multi-level interventions are needed to address health-system, healthcare provider and patient-level barriers to improve the use of GDMT for better outcomes among patients with HFrEF.

## Data Accessibility Statement

Datasets used in the research can be obtained from the corresponding author upon reasonable request.

## Additional File

The additional file for this article can be found as follows:

10.5334/gh.1355.s1 Supplementary File. Tables S1–Table S16 and Figure S1.
