# Supplementary material for: Use of Guideline-Recommended Heart Failure Drugs in High-, Middle-, and Low-Income Countries: A Systematic Review and Meta-Analysis
Source: Glob Heart. 2024 Sep 12;19(1):74. doi: 10.5334/gh.1355 (PMC11396255; doi:10.5334/gh.1355)

SUPPLEMENTARY FILE

CONTENTS

Table S1: Search strategy MEDLINE........ 2

Table S2: Search strategy Global Health........ 5

Table S3: Search strategy CINAHL........ 8

Table S4: Characteristics of the included studies 9

Table S5. Characteristics of registry-based studies 37

Table S6. Prevalence of GDMT in the acute and non-acute setting by WHO Regions........ 49

Table S7. Prevalence of GDMT in acute and non-acute settings by income setting........ 50

Table S8: Prevalence of Beta blocker use by countries. 51

Table S9: Prevalence of RAS inhibitor (angiotensin converting enzyme receptor inhibitor and/or angiotensin receptor II blocker) use by countries. 53

Table S10. Prevalence of mineralocorticoid receptor blockers (MRA) use by countries. 55

Table S11. Prevalence of Diuretic use by countries........ 57

Table S12. Prevalence of other Guideline-directed Medical Therapy (GDMT) by WHO regions. 59

Table S13: Prevalence of other GDMT by income status 61

Table S14. Quality assessment using Joanna Briggs Institute (JBI) critical appraisal tool........ 62

Table S15: Bivariate and multivariate sensitivity analyses for prevalence of use of beta-blockers........ 76

Table S16: Bivariate and multivariate sensitivity analyses for prevalence of use of RAS inhibitors........ 77

Figure S1: Bivariate scatter plot with meta-regression line y-axis= effect, X-axis = year of time publication 78

Table S1: Search strategy MEDLINE

Database(s): Ovid MEDLINE(R) 1946 to October 05, 2020

Search Strategy:	
#	Searches
1	heart failure.tw.
2	cardiac failure.tw.
3	cardiomyopathy*.tw.
4	((heart or cardiac or myocardial) adj2 (failure or decompensation)).tw.
5	CHF.tw.
6	HF.tw.
7	HFrEF.tw.
8	HFpEF.tw.
9	Heart Failure/
10	or/1-9
11	prescription*.tw.
12	(guideline* adj3 treatment*).tw.
13	(guideline* adj3 drug*).tw.
14	(guideline* adj3 medic*).tw.
15	GDMT.tw.
16	adherence.tw.
17	compliance.tw.
18	persistence.tw.
19	drug therapy.tw.
20	exp Angiotensin-Converting Enzyme Inhibitors/
21	acei.tw.
22	(angiotensin adj3 Inhibitor*).tw.
23	ace inhibitor*.tw.
24	exp Angiotensin Receptor Antagonists/
25	(angiotensin adj3 blocker*).tw.
26	(angiotensin adj3 antagonist*).tw.
27	exp angiotensin II type 1 receptor blockers/
28	arb?.tw.
29	exp adrenergic beta-antagonists/
30	(beta adj block*).tw.
31	(beta adj3 antagonist*).tw.
32	digoxin.tw.
33	lanoxin.tw.
34	Angiotensin-Receptor Neprilysin Inhibitors.tw.
35	(neprilysin adj3 Inhibitor*).tw.
36	ARNI*.tw.
37	If Channel Blocker*.tw.
38	If Channel inhibitor*.tw.
39	Mineralocorticoid Receptor Antagonists/
40	(aldosterone adj3 antagonist*).tw.

41	Diuretics/
42	diuretic*.tw.
43	Hydralazine/
44	hydralazine.tw.
45	Isosorbide Dinitrate/
46	isosorbide dinitrate.tw.
47	or/11-46
48	Population Surveillance/
49	population based.tw.
50	community.tw.
51	"Surveys and Questionnaires"/
52	survey.tw.
53	Sentinel Surveillance/
54	surveillance.tw.
55	Cohort Studies/
56	cohort.tw.
57	Cross-Sectional Studies/
58	cross-sectional.tw.
59	Registries/
60	registry.tw.
61	Observational Study/ or Observational Studies as Topic/
62	observational.tw.
63	Longitudinal Studies/
64	longitudinal.tw.
65	Qualitative Research/
66	qualitative.tw.
67	interview*.tw.
68	(focus adj group*).tw.
69	Clinical Trial/
70	Comparative Study/
71	Randomized Controlled Trials as Topic/
72	Non-Randomized Controlled Trials as Topic/
73	Randomized Controlled Trial/
74	experimental.tw.
75	trial.tw.
76	rct.tw.
77	random*.tw.
78	quasi.tw.
79	non-comparative.tw.
80	(time adj2 series).tw.
81	(interventional adj study).tw.
82	(non-randomi?ed adj study).tw.
83	or/48-82
84	10 and 47 and 83
85	limit 84 to humans

86	limit 85 to yr="2010 - 2019"

Table S2: Search strategy Global Health
Database(s): Global Health 1910 to 2020 Week 39

Search Strategy:	
#	Searches
1	heart failure.mp.
2	cardiac failure.mp.
3	cardiomyopathy*.mp.
4	((heart or cardiac or myocardial) adj2 (failure or decompensation)).mp.
5	CHF.mp.
6	HF.mp.
7	HFrEF.mp.
8	HFpEF.mp.
9	prescription*.mp.
10	(guideline* adj3 treatment*).mp.
11	(guideline* adj3 drug*).mp.
12	(guideline* adj3 medic*).mp.
13	GDMT.mp.
14	'Guideline directed medical therapy'.mp.
15	adherence.mp.
16	compliance.mp.
17	persistence.mp.
18	drug therapy.mp.
19	acei.mp.
20	(angiotensin adj3 Inhibitor*).mp.
21	ace inhibitor*.mp.
22	(angiotensin adj3 blocker*).mp.
23	(angiotensin adj3 antagonist*).mp.
24	arb?.mp.
25	exp adrenergic beta-antagonists/
26	(beta adj block*).mp.

27	(beta adj3 antagonist*).mp.
28	digoxin.mp.
29	Angiotensin-Receptor Neprilysin Inhibitors.mp.
30	(neprilysin adj3 Inhibitor*).mp.
31	ARNI*.mp.
32	(aldosterone adj3 antagonist*).mp.
33	Diuretics/
34	diuretic*.mp.
35	hydralazine.mp.
36	isosorbide dinitrate.mp.
37	population based.mp.
38	community.mp.
39	survey.mp.
40	Sentinel Surveillance/
41	surveillance.mp.
42	Cohort Studies/
43	cohort.mp.
44	cross-sectional.mp.
45	registry.mp.
46	(Observational adj2 Stud*).mp.
47	observational.mp.
48	Longitudinal Studies/
49	longitudinal.mp.
50	((Qualitative adj2 study) or research).mp.
51	qualitative.mp.
52	interview*.mp.
53	(focus adj group*).mp.
54	Clinical Trial/
55	"Randomized Controlled Trials".mp.
56	"Non Randomized Controlled Trials".mp.
57	Randomized Controlled Trial/

58	experimental.mp.
59	trial.mp.
60	rct.mp.
61	random*.mp.
62	quasi.mp.
63	non-comparative.mp.
64	(time adj2 series).mp.
65	(interventional adj study).mp.
66	(non-randomi?ed adj study).mp.
67	or/1-8
68	or/9-36
69	or/37-66
70	67 and 68 and 69
71	limit 70 to yr="2010 - 2019"

Table S3: Search strategy CINAHL
Database(s): CINAHL

**S1 AND S2 AND S3**	**Limiters - Date Published: 20100101-20191231**
S4	S1 AND S2 AND S3
S3	TX (Cross Sectional Studies) or (Nonexperimental Studies+) or (cohort studies) or (Observational Methods+) or (Quasi-Experimental Studies+) or (Randomized Controlled Trials+) or (Community Trials) or (Clinical Trials+) or (Preventive Trials) or (Intervention Trials) or (Equivalence Trials) or (Therapeutic Trials) or survey or questionnaire or interview or (qualitative study) or (qualitative research) or registry
S2	TX (drug prescriptions) or (guideline adherence) or (prescription drugs) or (medication compliance) or (medication adherence) or (patient compliance) or (Angiotensin-Converting Enzyme Inhibitors+) or (Angiotensin II Type I Receptor Blockers+) or (Adrenergic Beta-Antagonists+) or (Angiotensin-Receptor Neprilysin Inhibitors+) or (ARNI) or digoxin or lanoxin or (Diuretics, Potassium Sparing+) or diuretics or furosemide or torsemide or hydralazine or (Diuretics, Thiazide+)
S1	TX heart failure or cardiac failure or chf or chronic heart failure or congestive heart failure or hfref or hfpef or heart failure with reduced ejection fraction or heart failure with preserved ejection fraction or cardiac decompensation or heart arrest

Table S4: Characteristics of the included studies

Obs.	Author (Year)	Settings	Country	Recruitment period (years)	Sample Size (n)	Mean Age	Female patients (%)	LVEF (%)		Data source for medication use	Type of medication data	Medications studied
								Eligibility	Mean			
Africa												
1.	Abebe, 2016	Acute	Ethiopia	2010-2015	147	52.9	62.6	<50	40.4	Not Reported	Discharge	BB,RASi,MRA,DIG,DIUR
2.	Niriayo, 2019	Non-acute	Ethiopia	2016	308	52.3	48.5	<40		Records/Prescriptions	Baseline	BB,RASi,MRA,DIG,DIUR
3.	Bonsu, 2017	Acute	Ghana	2009-2013	610	59.5	51.0	<50	36.9	Records/Prescriptions	Discharge	BB,RASi,MRA,DIG,DIUR
4.	Ruf, 2010	Non-acute	South Africa	2006-2007	200	56.0	45.5	<=45	32.0	Self-Reported, Records/Prescriptions	Baseline	BB,RASi,MRA,DIG,DIUR
5.	A Makubi, 2016	Non-acute	Tanzania	2012-2013	411	55.0	50.0	<50		Not Reported	Baseline	BB,RASi,MRA,DIG,DIUR
Americas												
6.	Ajam, 2018	Non-acute	US	2007-2015	114010	68.3	2.0	<=40		Not Reported	Baseline	RASI,MRA,DIG,DIUR
7.	AlJaroudi, 2015	Non-acute	US	2002-2010	1509	63.2	19.7	<=30	20.8	Not Reported	Baseline	BB,RASi,MRA,DIG,DIUR
8.	Albert, 2010	Non-acute	US	2005-2007	15381	68.7	29.0	<=35	25.4	Records/Prescriptions	Baseline	BB,RASI,MRA
9.	Alkhawam, 2019	Non-acute	US	2005-2014	1047	65.9	33.4	<=40	25.6	Not Reported	Baseline	BB,RASI,DIG,DIUR
10.	Allen, 2014	Non-acute	US	2005-2008	490	73.6	37.1	<=40	25.0	Records/Prescriptions	Baseline	BB,RASi,MRA,DIG,DIUR
11.	Allen, 2018	Non-acute	US	2008-2014	75107	68.6	30.9	<40		Records/Prescriptions	Baseline	BB,RASI,DIUR
12.	Alvarez, 2019	Non-acute	US	2011-2014	40966	18–65*	36.4			Records/Prescriptions	Baseline	BB,RASI,MRA,DIUR
13.	Antol, 2018	Non-acute	US	2015-2016	200	72.0	61.0			Records/Prescriptions	Baseline	BB,RASI,MRA,DIG,DIUR,HYD/NIT
14.	Arnold, 2019	Non-acute	US	2013-2016	28877	70.0	30.0	<40	27.7	Not Reported	Baseline	BB,RASI,DIUR

Obs.	Author (Year)	Settings	Country	Recruitment period (years)	Sample Size (n)	Mean Age	Female patients (%)	LVEF (%)		Data source for medication use	Type of medication data	Medications studied
								Eligibility	Mean			
15.	Asghar, 2010	Non-acute	US	2005-2006	563	62.4	29.8	<=35	24.3	Records/Prescriptions	Follow-up	BB,RASI,DIUR
16.	Ayan, 2019	Non-acute	US	2012-2017	225	58.6	40.0	<=40	24.9	Records/Prescriptions	Baseline	RASI,MRA,DIG,DIUR
17.	Aziz, 2011	Non-acute	US	NR	116	71.0	41.4		21.0	Records/Prescriptions	Baseline	MRA,DIUR
18.	Bayoumi, 2019	Acute	US	2003-2004	6986	75.9	40.2	<=35	24.9	Records/Prescriptions	Discharge	BB,RASi,MRA,DIG,DIUR
19.	Bhatia, 2015	Acute	US	1998-2001	2202	74.7	46.6	<45	27.8	Not Reported	Discharge	BB,RASI,MRA,DIG,DIUR,HYD/NIT
20.	Bhattacharya, 2010	Non-acute	US	2002-2008	759	66.4	18.7	<=35	22.0	Records/Prescriptions	Baseline	BB,RASI,MRA
21.	Blecker, 2014	Acute	US	2005-2009	4345	75.1	53.0	<50		Records/Prescriptions	Discharge	BB,RASI,MRA
22.	Butler, 2019	Non-acute	US	2011-2014	11064	69.3	35.2	<50	32.5	Records/Prescriptions	Baseline	BB,RASI,MRA
23.	Chang, 2013	Non-acute	US	2006-2008	668	.	33.5	<=40		Records/Prescriptions	Baseline	RASI,DIUR
24.	Church, 2015	Non-acute	US	2000-2008	272	69.0	.	<40	25.6	Not Reported	Baseline	RASI,MRA,DIG,DIUR
25.	Chyu, 2014	Non-acute	US	2000-2011	2255	53.1	30.4		27.8	Records/Prescriptions	Baseline	BB,RASI,DIG,DIUR
26.	Coles, 2015	Acute	US	1995-2006	1935	74.1	44.4	<=49		Not Reported	Discharge	BB,RASi,MRA,DIG,DIUR
27.	Crissinger, 2015	Non-acute	US	2009-2010	641	61.8	40.2	<=40	29.8	Records/Prescriptions	Baseline	BB,RASI
28.	Cutshall, 2018	Non-acute	US	2013-2015	109	63.0	39.0	<40		Records/Prescriptions	Baseline	BB,RASI,MRA,DIG,DIUR,HYD/NIT
29.	DeVore, 2016	Non-acute	US	2008-2010	722	62.0	37.0	<40	30.0	Not Reported	Baseline	BB,RASI,MRA,DIG
30.	DeVore, 2016	Acute	US	2003-2004	10696	68.9	41.4	<40		Not Reported	Discharge	BB,RASI,MRA

Obs.	Author (Year)	Settings	Country	Recruitment period (years)	Sample Size (n)	Mean Age	Female patients (%)	LVEF (%)		Data source for medication use	Type of medication data	Medications studied
								Eligibility	Mean			
31.	DeVore, 2018	Non-acute	US	2015-2017	4216	66.1	29.8	<=40	29.3	Not Reported	Baseline	BB,RASI,MRA,ARNI,IVA,HYD/NIT
32.	DeWolfe, 2010	Non-acute	US	1999-2007	364	54.7	37.1	<=40	29.5	Not Reported	Baseline	BB,RASI,MRA,DIUR
33.	Dec, 2014	Non-acute	US	NR	373	45.0	10.0	<=40	24.0	Not Reported	Baseline	BB,RASI,MRA,DIUR
34.	Desai, 2010	Non-acute	US	NR	549	74.0	20.9		28.9	Not Reported	Baseline	BB,RASI,DIG
35.	Dev, 2015	Non-acute	US	2003-2009	13411	71.0	1.0	<40		Not Reported	Baseline	BB,RASi,MRA,DIG,DIUR
36.	DiDomenico, 2014	Non-acute	US	2003-2006	131	60.0	48.0			Not Reported	Baseline	BB,RASI,MRA,DIUR
37.	Dunlay, 2011	Non-acute	US	2007-2009	101	73.7	41.0			Records/Prescriptions	Follow-up	BB,RASi,MRA,DIG,DIUR
38.	Durstenfeld, 2019	Acute	US	2013-2016	1009	71.9	29.5	<=35	26.4	Records/Prescriptions	Discharge	BB,RASI,DIUR
39.	El-Chami, 2010	Non-acute	US	2003-2004	25025	66.3	28.5	<=40	31.1	Self-reported, Records/Prescriptions	Baseline	BB,RASI
40.	El-Refai, 2013	Non-acute	US	2000-2008	1094	67.5	41.2		28.6	Records/Prescriptions	Baseline	BB,RASI
41.	Elmariah, 2010	Non-acute	US	2000-2003	580	52.0	28.0	<=45	26.0	Records/Prescriptions	Baseline	BB,RASI,MRA,DIG,DIUR,HYD/NIT
42.	Fleming, 2016	Non-acute	US	2009-2012	26658	68.5	42.9	<40		Records/Prescriptions	Baseline	BB,RASI
43.	Fonarow, 2011	Non-acute	US	NR	11621	70.0	28.9	<=35		Records/Prescriptions	Baseline	BB,RASI,MRA
44.	Fontaine, 2016	Non-acute	US	2002-2013	131	65.0	47.0		22.0	Not Reported	Baseline	BB,RASI,MRA,DIUR,HYD/NIT
45.	Gagne, 2018	Non-acute	US	2013-2016	9747	70.6	33.7	<=40	30.1	Records/Prescriptions	Baseline	BB,RASi,MRA,DIG,DIUR
46.	Gilstrap, 2018	Acute	US	2013-2014	148	69.0	42.0	<=40		Records/Prescriptions	Discharge	BB,RASI
47.	Gurwitz, 2017	Non-acute	US	2005-2008	986	71.0	41.2	<=40		Records/Prescriptions	Baseline	BB,RASi,MRA,DIG,DIUR

Obs.	Author (Year)	Settings	Country	Recruitment period (years)	Sample Size (n)	Mean Age	Female patients (%)	LVEF (%)		Data source for medication use	Type of medication data	Medications studied
								Eligibility	Mean			
48.	Han, 2019	pakisNon-acute	US	2015-2017	263	70.4	1.5	<=40	31.1	Not Reported	Baseline	BB,RASi,MRA,DIG,DIUR
49.	Hebert, 2010	Non-acute	US	2007-2009	561	57.2	31.4	<=40	24.6	Self-Reported	Baseline	BB,RASI
50.	Hebert, 2011	Non-acute	US	1999-2007	1000	57.5	34.0	<=40	27.5	Not Reported	Baseline	BB,RASI
51.	Hernandez, 2012	Acute	US	2005-2009	5887	77.6	35.7	<=35	25.4	Not Reported	Discharge	BB,RASI,DIG,DIUR
52.	Inampudi, 2014	Acute	US	1998-2001	1140	76.0	49.0	<45	28.0	Not Reported	Discharge	BB,RASI,DIG,DIUR
53.	Jehu S. Mathew, 2017	Non-acute	US	2013-2014	36786	73.2	27.1	<40	30.1	Records/Prescriptions	Baseline	BB,DIG
54.	Kelesidis, 2013	Non-acute	US	NR	418	64.0	35.9	<=40	30.0	Self-Reported, Records/Prescriptions	Baseline	BB,RASI,MRA,DIG
55.	Kim, 2019	Non-acute	US	2008-2014	6915	69.1	35.1	<50		Records/Prescriptions	Baseline	BB,RASI,MRA
56.	Krishnan, 2015	Non-acute	US	2002-2004	109	53.0	28.0	<=35	21.0	Not Reported	Baseline	BB,RASI,DIG
57.	Laliberte, 2017	Acute	US	2013-2015	123	58.7	30.1		24.2	Not Reported	Discharge	BB,RASI,MRA,DIG,DIUR,HYD/NIT
58.	Lam, 2017	Acute	US	1998-2001	1874	73.6	44.4	<=35	25.1	Not Reported	Discharge	BB,RASi,MRA,DIG,DIUR
59.	Lam, 2018	Acute	US	1998-2001	634	73.0	45.4	<45	29.4	Records/Prescriptions	Discharge	BB,RASi,MRA,DIG,DIUR
60.	Lanfear, 2012	Acute	US	2000-2008	1094	67.5	41.0	<50	28.6	Records/Prescriptions	Discharge	BB,RASI
61.	Lee, 2013	Non-acute	US	2006-2008	2358	69.2	33.9	<40		Not Reported	Baseline	BB,RASi,MRA,DIG,DIUR
62.	Lee, 2015	Non-acute	US	2010-2013	202	56.9	50.0		28.6	Not Reported	Baseline	BB,RASI,MRA
63.	Li, 2018	Non-acute	US	2011-2012	41340	.	58.1	<45		Records/Prescriptions	Baseline	BB,RASi,MRA,DIG,DIUR

Obs.	Author (Year)	Settings	Country	Recruitment period (years)	Sample Size (n)	Mean Age	Female patients (%)	LVEF (%)		Data source for medication use	Type of medication data	Medications studied
								Eligibility	Mean			
64.	Li, 2019	Non-acute	US	2008-2012	1852	68.3	43.8	40-49	43.5	Not Reported	Baseline	BB,RASI,MRA,HYD/NIT
65.	Lin, 2010	Non-acute	US	1993-2007	1369	64.6	22.5	<=40	30.8	Records/Prescriptions	Baseline	BB,RASI
66.	Loh, 2013	Non-acute	US	1993-2010	2507	53.4	25.9	<=40	23.3	Records/Prescriptions	Baseline	BB,RASI,MRA,DIUR
67.	Luo, 2018	Acute	US	2016	3738	70.0	35.7	<=40	25.0	Not Reported	Discharge	BB,RASI,MRA
68.	Luo, 2019	Acute	US	2015-2016	16674	69.0	35.4	<=40	25.0	Records/Prescriptions	Discharge	BB,RASI,MRA,ARNI
69.	Luzum, 2019	Non-acute	US	2007-2015	951	68.3	35.9	<50	34.7	Records/Prescriptions	Baseline	BB,RASI
70.	M Fudim, 2018	Non-acute	US	2007-2015	111970	68.5	0.0			Records/Prescriptions	Baseline	RASI,MRA,DIG,DIUR
71.	Mathew, 2017	Non-acute	US	2013-2014	36786	73.2	27.1	<40	30.1	Records/Prescriptions	Baseline	BB,DIG
72.	McNamara, 2011	Non-acute	US	2002-2008	373	45.0	38.0	<=40	24.0	Not Reported	Baseline	BB,RASI,MRA,DIUR
73.	Mefford, 2019	Non-acute	US	2013-2016	124	77.2	43.0	<50		Not Reported	Baseline	BB,RASI
74.	Mohanty, 2019	Non-acute	US	2015-2017	38677	69.2	1.7	<=40	29.4	Records/Prescriptions	Baseline	BB,RASI,MRA,ARNI,DIG,DIUR, HYD/NIT
75.	Mojadidi, 2016	Non-acute	US	2005-2011	1907	65.5	39.5	<40	28.9	Not Reported	Baseline	DIG,DIUR
76.	Murphy, 2019	Non-acute	US	2013-2015	100	68.0	58.0		35.0	Records/Prescriptions	Baseline	BB,RASi,MRA,DIG,DIUR
77.	Nagatomo, 2017	Non-acute	US	2002-2008	353	45.0	38.8	<40		Not Reported	Baseline	BB,RASi,MRA,DIG,DIUR
78.	Pandey, 2016	Acute	US	2005-2014	111846	69.9	36.0	<40	25.1	Records/Prescriptions	Discharge	BB,RASI
79.	Parakh, 2012	Non-acute	US	1997-2004	171	48.6	42.0		25.0	Not Reported	Baseline	BB,RASi,MRA,DIG,DIUR
80.	Patel, 2016	Non-acute	US	2005-2014	117761	68.9	36.3			Not Reported	Baseline	BB,RASi,MRA,DIG,DIUR
81.	Qamer, 2019	Acute	US	2003-2004	8401	76.0	43.7	<=45	29.4	Not Reported	Discharge	BB,RASI,MRA,DIUR

Obs.	Author (Year)	Settings	Country	Recruitment period (years)	Sample Size (n)	Mean Age	Female patients (%)	LVEF (%)		Data source for medication use	Type of medication data	Medications studied
								Eligibility	Mean			
82.	Richardson, 2016	Acute	US	2010-2015	281	67.8	40.9	<=40		Records/Prescriptions	Discharge	BB,RASI,MRA
83.	Rickard, 2014	Non-acute	US	2002-2008	879	66.5	29.9		22.5	Records/Prescriptions	Baseline	BB,RASI,DIUR
84.	Roth, 2016	Non-acute	US	2007-2011	19773	74.9	35.4	<=40	25.6	Records/Prescriptions	Baseline	BB,RASI
85.	Sanam, 2016	Acute	US	1998-2001	1384	74.9	46.5	<45	28.5	Not Reported	Discharge	BB,RASi,MRA,DIG,DIUR
86.	Sartipy, 2014	Non-acute	US	NR	180	52.7	26.0		21.0	Records/Prescriptions	Baseline	BB,RASI,MRA
87.	Schneider, 2014	Non-acute	US	2006-2008	45392	69.3	31.5	<=35		Not Reported	Baseline	BB,RASI
88.	Shen, 2013	Non-acute	US	NR	136	70.0	27.9	<=35	20.6	Not Reported	Baseline	BB,RASi,MRA,DIG,DIUR
89.	Shore, 2012	Non-acute	US	1998-2008	3716	60.9	53.3	<=40	31.6	Records/Prescriptions	Baseline	BB,RASi,MRA,DIG,DIUR
90.	Shreibati, 2016	Acute	US	2009-2015	1458	60.0	29.6	<=25		Not Reported	Discharge	BB,RASI,MRA,HYD/NIT
91.	Steinman, 2011	Non-acute	US	2004	2772	71.0	8.0	<40		Records/Prescriptions	Baseline	BB,RASI
92.	Stewart, 2016	Non-acute	US	2010-2011	166	57.0	29.0	<=30	18.0	Not Reported	Baseline	BB,RASI,MRA,DIUR,HYD/NIT
93.	Tran, 2018	Non-acute	US	2005-2011	1585	74.0	45.0	<50	30.0	Not Reported	Baseline	BB,RASi,MRA,DIG,DIUR
94.	Ujeyl, 2011	Non-acute	US	2007-2009	122	57.0	57.4	<=40	25.5	Not Reported	Baseline	BB,RASI,DIG,DIUR
95.	Valika, 2018	Non-acute	US	2004-2014	244	63.7	38.5	<=35	23.0	Not Reported	Follow-up	BB,RASI,MRA
96.	WA Teeter, 2012	Non-acute	US	1998-2006	270	52.0	37.0	<30		Records/Prescriptions	Baseline	BB,RASI,MRA
97.	Wang, 2016	Non-acute	US	2005-2015	102	57.9	46.0	<=35	21.5	Not Reported	Discharge	BB,RASi,MRA,DIG,DIUR
98.	Wang, 2018	Non-acute	US	1998-2016	123	62.4	55.0	<=35	22.8	Not Reported	Baseline	BB,RASi,MRA,DIG,DIUR
99.	Wilcox, 2012	Non-acute	US	NR	3994	66.6	29.7	<=35	25.8	Records/Prescriptions	Baseline	BB,RASI,MRA,DIG

Obs.	Author (Year)	Settings	Country	Recruitment period (years)	Sample Size (n)	Mean Age	Female patients (%)	LVEF (%)		Data source for medication use	Type of medication data	Medications studied
								Eligibility	Mean			
100.	Woodruff, 2016	Acute	US	2012	131	66.7	35.9	<=40	25.2	Records/Prescriptions	Discharge	BB,RASI,MRA,DIUR
101.	Wu, 2013	Acute	US	1999-2007	461	64.1	33.0	<40	33.8	Not Reported	Discharge	BB,RASI,DIG,DIUR
102.	Zepeda, 2019	Non-acute	US	2000-2016	22683	67.3	43.6	<40	30.6	Records/Prescriptions	Baseline	BB,RASI,MRA,DIUR,HYD/NIT
103.	Ziaeian, 2017	Non-acute	US	2007-2013	5168	65.2	2.3	<40	23.3	Not Reported	Baseline	BB,RASI
104.	Albuquerque, 2014	Non-acute	Brazil	2009-2012	111	59.5	39.6	<=50	34.0	Records/Prescriptions	Baseline	BB,RASI,MRA,DIG,DIUR
105.	Carlo, 2014	Non-acute	Brazil	1992-2000, 2005-2006	333	58.7	36.0	<40	28.0	Not Reported	Baseline	BB,RASi,MRA,DIG,DIUR
106.	Correa, 2016	Acute	Brazil	2006-2008	1052	70.6	29.9	<40	30.5	Records/Prescriptions	Discharge	BB,RASI
107.	Lindemberg, 2014	Non-acute	Brazil	NR	154	61.5	44.2	<50	41.7	Not Reported	Baseline	BB,RASI,DIG,DIUR
108.	Moreno, 2013	Non-acute	Brazil	2011-2012	104	64.1	33.7	<45	37.3	Not Reported	Baseline	RASI,MRA,DIG
109.	Olsen, 2014	Non-acute	Brazil	2003-2007	184	62.0	32.6	<45	31.0	Not Reported	Baseline	BB,RASI
110.	Passos, 2016	Non-acute	Brazil	2008-2012	194	62.4	39.7	<=40	31.1	Records/Prescriptions	Baseline	BB,RASI
111.	Alba, 2013	Non-acute	Canada	2010-2011	121	56.0	21.0	<40	29.0	Not Reported	Baseline	BB,RASI,MRA,DIG,DIUR
112.	Azizi-Namini, 2019	Non-acute	Canada	2009-2011	100	65.0	18.0	<=45		Not Reported	Baseline	BB,RASI,MRA,DIG,DIUR
113.	Diamant, 2019	Non-acute	Canada	2015-2017	370	66.2	29.0	<=40	26.6	Not Reported	Baseline	BB,RASi,MRA,DIG,DIUR
114.	Ghimire, 2019	Non-acute	Canada	2008-2016	3124	66.8	27.6	<=40	28.7	Not Reported	Baseline	BB,RASI,MRA,ARNI,DIG,DIUR
115.	Roth, 2017	Non-acute	Canada	2014-2015	356	71.5	29.2	<=35	27.2	Records/Prescriptions	Baseline	BB,RASI,MRA,DIG
116.	Klassen, 2018	Acute	Guyana	2015-2017	232	60.4	51.3	<40		Records/Prescriptions	Discharge	BB,RASI,MRA

Obs.	Author (Year)	Settings	Country	Recruitment period (years)	Sample Size (n)	Mean Age	Female patients (%)	LVEF (%)		Data source for medication use	Type of medication data	Medications studied
								Eligibility	Mean			
117.	Gonzalez-Zuelgaray, 2013	Acute	Argentina, Chile, Colombia, Mexico, Dominican Republic	2008-2009	153	67.1	24.0	<=35	26.3	Not Reported	Discharge	BB,RASI,MRA
Eastern Mediterranean												
118.	Mohamadi, 2012	Non-acute	Iran	2010	100	59.2	0.0	<40	35.2	Not Reported	Baseline	BB,RASi,MRA,DIG,DIUR
119.	El-Menyar, 2015	Non-acute	Qatar	2003-2013	1776	61.0	26.4	<50		Records/Prescriptions	Baseline	BB,RASI,DIUR
120.	Rahhal, 2017	Acute	Qatar	2013-2014	400	.	21.0	<=40		Records/Prescriptions	Discharge	BB,RASi,MRA,DIG,DIUR
121.	AlShamiri, 2018	Acute	Saudi Arabia	2009-2010	387	61.6	20.4	<=40		Not Reported	Discharge	BB,RASI,DIG,DIUR
122.	Al-khateeb, 2017	Non-acute	Saudi Arabia	2000-2015	2298	57.2	28.3	<45	29.5	Records/Prescriptions	Baseline	BB,RASi,MRA,DIG,DIUR
123.	Atallah, 2019	Non-acute	UAE	2016-2017	102	58.3	35.3	<=40		Records/Prescriptions	Baseline	BB,RASI,MRA,DIUR
124.	Abi Khalil, 2017	Non-acute	Oman, Saudi Arabia, UAE, Qatar, Bahrain, Yemen, Kuwait	2012	1278	60.8	27.4	<40	27.0	Not Reported	Baseline	RASi,MRA,IVA,DIUR
125.	Abi Khalil, 2018	Acute	Bahrain, Kuwait, Oman, Qatar, UAE, Yemen	2009-2010	334	62.0	34.0	<40	31.0	Not Reported	Discharge	BB,RASI,DIG,DIUR
Europe												
	Hebert, 2011	Non-acute	Georgia	1999-2007	400	57.5	34.0	<=40	27.5	Not Reported	Baseline	BB,RASI,DIUR

Obs.	Author (Year)	Settings	Country	Recruitment period (years)	Sample Size (n)	Mean Age	Female patients (%)	LVEF (%)		Data source for medication use	Type of medication data	Medications studied
								Eligibility	Mean			
126.	Frohlich, 2016	Non-acute	Germany	1995-2012	381	69.0	32.0	<45	32.0	Not Reported	Baseline	BB,MRA,DIG,DIUR
127.	Cleland, 2011	Non-acute	UK	2008-2009	3590	76.0	35.0	<=40		Not Reported	Discharge	BB,RASI,MRA,DIUR
128.	Cubbon, 2011	Non-acute	UK	2006-2009	357	66.0	29.0	<=45	31.0	Not Reported	Baseline	BB,RASi,MRA,DIG,DIUR
129.	Cubbon, 2019	Non-acute	UK	2006-2014	1802	69.6	26.8	<=45	32.0	Not Reported	Baseline	BB,RASI,MRA
130.	D Cullington, 2011	Non-acute	UK	2000-2010	2211	72.0	27.0	<=50		Not Reported	Baseline	BB,RASi,MRA,DIG,DIUR
131.	Dierckx, 2015	Non-acute	UK	2013	454	73.0	24.4	<50	35.7	Not Reported	Baseline	BB,RASI,MRA,IVA,DIUR
132.	Frankenstein, 2011	Non-acute	UK	1995-2005	1312	64.9	21.6	<40	31.1	Not Reported	Baseline	BB,RASI,MRA,DIUR
	Frohlich, 2016	Non-acute	UK	1995-2012	341	69.0	32.0	<45	32.0	Not Reported	Baseline	BB,MRA,DIG,DIUR
133.	Shelton, 2010	Non-acute	UK	NR	891	70.0	29.3	<45	32.0	Not Reported	Baseline	BB,RASi,MRA,DIG,DIUR
134.	Taylor, 2012	Non-acute	UK	1995-1999	328	70.4	25.9	<40		Not Reported	Baseline	BB,RASI,DIUR
135.	Walker, 2016	Non-acute	UK	2006-2009	628	67.2	26.3	<45	31.2	Not Reported	Baseline	BB,RASI,MRA,DIUR
136.	Witte, 2018	Non-acute	UK	2006-2014	1797	69.6	26.8	<=45	32.0	Records/Prescriptions	Baseline	BB,RASI
137.	Adlbrecht, 2010	Non-acute	Austria	NR	205	65.7	22.4		27.6	Not Reported	Follow-up	BB,MRA,DIG,DIUR
138.	Bartko, 2019	Non-acute	Austria	NR	423	66.0	21.0		26.0	Not Reported	Baseline	BB,RASI,MRA,DIUR
139.	Neuhold, 2010	Non-acute	Austria	NR	181	70.0	35.0		29.0	Not Reported	Baseline	BB,RASI,MRA,DIUR
140.	Poelzl, 2014	Non-acute	Austria	2006-2010	1014	65.0	27.4	<=40		Not Reported	Baseline	BB,RASi,MRA,DIG,DIUR
141.	Rychli, 2011	Non-acute	Austria	NR	351	75.0	34.0	<=40	30.0	Not Reported	Baseline	BB,RASI
142.	Wurm, 2017	Non-acute	Austria	2009-2013	128	66.0	16.0	<=35	26.0	Not Reported	Baseline	BB,RASI,MRA

Obs.	Author (Year)	Settings	Country	Recruitment period (years)	Sample Size (n)	Mean Age	Female patients (%)	LVEF (%)		Data source for medication use	Type of medication data	Medications studied
								Eligibility	Mean			
143.	De Sutter, 2015	Acute	Belgium	2008-2012	543	75.3	34.7	<=49	30.7	Not Reported	Discharge	BB,RASI,MRA
144.	Houard, 2019	Non-acute	Belgium	2002-2015	266	60.0	29.0	<=35	23.0	Not Reported	Baseline	BB,RASi,MRA,DIG,DIUR
145.	Lau, 2019	Non-acute	Belgium	2016-2018	201	67.7	18.0	<35		Records/Prescriptions	Baseline	BB,RASI,MRA,DIUR
146.	Martens, 2017	Non-acute	Belgium	2008-2015	687	71.8	32.5		29.2	Records/Prescriptions	Baseline	BB,RASI,MRA,DIUR
147.	Martens, 2018	Non-acute	Belgium	2016-2017	120	66.0	19.0	<35	26.0	Records/Prescriptions	Baseline	BB,MRA,DIUR
148.	Martens, 2018	Non-acute	Belgium	2016-2017	125	66.0	19.0	<35	29.6	Not Reported	Baseline	BB,MRA,DIUR
149.	Martens, 2019	Non-acute	Belgium	2016-2018	151	67.7	32.3	<35	29.0	Records/Prescriptions	Baseline	BB,MRA,IVA,DIG,DIUR
150.	Martens, 2019	Non-acute	Belgium	2016-2018	201	67.7	18.0	<35	29.0	Records/Prescriptions	Baseline	BB,MRA,DIUR
151.	Nasser, 2017	Non-acute	Belgium	2007-2013	163	62.0	24.0	<=40	29.0	Records/Prescriptions	Baseline	BB,RASI,MRA,DIUR
152.	Vandenberk, 2016	Non-acute	Belgium	1996-2014	244	56.6	26.2		27.6	Not Reported	Baseline	BB,RASI,DIG,DIUR
153.	Wohlfahrt, 2015	Non-acute	Czech Republic	2007-2011	369	59.0	16.0	<50	25.0	Not Reported	Baseline	BB,RASI,MRA,DIUR
154.	Anne Nakano1, 2019	Non-acute	Denmark	2003-2010	17758	70.9	36.4	<=40		Records/Prescriptions	Baseline	BB,RASI
155.	Balling, 2011	Non-acute	Denmark	2002-2006	3465	68.3	27.4		32.0	Not Reported	Baseline	BB,RASi,MRA,DIG,DIUR
156.	Gjesing, 2013	Non-acute	Denmark	2002-2009	8792	69.0	28.0	<45	30.0	Records/Prescriptions	Baseline	BB,RASI,MRA
157.	Pasternak, 2014	Non-acute	Denmark	2003-2012	11664	69.2	29.3	<=40		Not Reported	Baseline	BB,RASI,MRA,DIUR
158.	Veien, 2011	Non-acute	Denmark	2006	3346	69.1	28.1	<=45	30.0	Records/Prescriptions	Baseline	BB,RASi,MRA,DIG,DIUR
159.	Bitar, 2019	Acute	France	2011-2012	354	.	35.0	<40		Records/Prescriptions	Discharge	BB,RASI,MRA

Obs.	Author (Year)	Settings	Country	Recruitment period (years)	Sample Size (n)	Mean Age	Female patients (%)	LVEF (%)		Data source for medication use	Type of medication data	Medications studied
								Eligibility	Mean			
160.	Boully, 2019	Non-acute	France	2012	234	88.2	68.9			Not Reported	Baseline	BB,RASI,MRA,IVA,DIG,DIUR
161.	Busson, 2018	Acute	France	2011-2012	624	73.8	35.4	<40	28.2	Records/Prescriptions	Discharge	BB,RASI,MRA,DIUR
162.	Cohen Solal, 2012	Non-acute	France	2007-2008	792	71.0	26.0	<=40		Records/Prescriptions	Discharge	BB,RASI,DIUR
163.	Juilliere, 2014	Non-acute	France	2007-2010	2394	66.2	26.1	<=50	34.2	Records/Prescriptions	Baseline	BB,RASi,MRA,DIG,DIUR
164.	Koukoui, 2015	Non-acute	France	2007-2013	202	58.0	21.5	<45	30.0	Not Reported	Baseline	BB,RASI,MRA,DIUR
165.	Hebert, 2010	Non-acute	Georgia	2007-2008	180	62.7	0.0	<=40	31.4	Not Reported	Baseline	BB,RASI
166.	Hebert, 2011	Non-acute	Georgia	2007-2008	400	63.7	27.4	<=40	32.2	Self Reported	Baseline	BB,RASI
167.	Angermann, 2011	Non-acute	Germany	NR	702	67.0	29.0	<=40	30.2	Not Reported	Baseline	BB,RASi,MRA,DIG,DIUR
168.	Bohm, 2015	Non-acute	Germany	2012	364	68.7	28.3	<50		Not Reported	Baseline	BB,RASI,MRA,IVA,DIUR
169.	Frankenstein, 2010	Non-acute	Germany	1994-2007	3292	60.8	22.3		28.2	Records/Prescriptions	Baseline	BB,RASi,MRA,DIG,DIUR
170.	Grimm, 2015	Non-acute	Germany	2007-2011	267	60.0	25.0	<=50	34.0	Not Reported	Baseline	BB,RASi,MRA,DIG,DIUR
171.	Muller, 2017	Non-acute	Germany	2010-2015	143	62.0	24.0	<45		Not Reported	Baseline	BB,RASi,MRA,DIG,DIUR
172.	Oldenburg, 2016	Non-acute	Germany	2002-2013	963	65.0	19.5	<45	29.9	Not Reported	Baseline	BB,RASi,MRA,DIG,DIUR
173.	Pfister, 2011	Non-acute	Germany	2006	125	57.0	22.4	<50	30.0	Not Reported	Baseline	BB,RASI,MRA,DIUR
174.	von Scheidt, 2014	Non-acute	Germany	2009-2011	1803	70.0	24.3	<=40	30.0	Not Reported	Baseline	BB,RASI,MRA,IVA,DIG,DIUR
175.	Simopoulos, 2015	Non-acute	Greece	NR	332	64.2	10.2	<=45	36.0	Not Reported	Baseline	BB,RASI
176.	Goland, 2011	Non-acute	Israel	2005-2007	100	69.9	21.0	<40		Not Reported	Baseline	BB,RASI,DIG,DIUR
177.	Murninkas, 2019	Non-acute	Israel	2016-2017	552	73.0	22.6	<=40	35.0	Records/Prescriptions	Baseline	BB,RASI,MRA

Obs.	Author (Year)	Settings	Country	Recruitment period (years)	Sample Size (n)	Mean Age	Female patients (%)	LVEF (%)		Data source for medication use	Type of medication data	Medications studied
								Eligibility	Mean			
178.	Aleksova, 2011	Non-acute	Italy	1988-2007	491	45.0	25.0		30.0	Not Reported	Baseline	BB,RASI,DIUR
179.	Bertero, 2019	Non-acute	Italy	2004-2015	570	69.0	25.6	<50	32.4	Not Reported	Baseline	BB,RASI,MRA,DIUR
180.	Bobbo, 2017	Non-acute	Italy	2005-2010	310	54.0	30.0	<50	33.6	Not Reported	Baseline	BB,RASI,MRA
181.	Boriani, 2012	Non-acute	Italy	NR	659	66.3	9.9	<=35	26.0	Not Reported	Baseline	BB,RASI,DIUR
182.	Campodonico, 2018	Non-acute	Italy	NR	958	67.0	16.0	<40	33.9	Not Reported	Baseline	RASI,MRA,DIG,DIUR
183.	Castelli, 2013	Non-acute	Italy	1977-2011	603	53.0	27.0		32.0	Not Reported	Baseline	BB,RASi,MRA,DIG,DIUR
184.	Corra, 2013	Non-acute	Italy	1995-2009	529	60.0	22.0	<=40	23.0	Records/Prescriptions	Baseline	BB,RASI,MRA,DIUR
185.	Fontanive, 2013	Non-acute	Italy	2002-2010	638	68.0	22.0	<=45	32.0	Not Reported	Baseline	BB,RASI,DIUR
186.	Fragasso, 2013	Non-acute	Italy	1992-2005	372	66.0	27.7	<=45		Records/Prescriptions	Baseline	BB,RASi,MRA,DIG,DIUR
187.	Fragasso, 2013	Non-acute	Italy	2002-2010	669	65.0	26.2	<45	33.1	Records/Prescriptions	Baseline	BB,DIG,DIUR
188.	Francia, 2015	Non-acute	Italy	NR	183	64.0	15.0	<50	28.0	Not Reported	Baseline	BB,RASI
189.	Franco, 2014	Non-acute	Italy	2012-2013	528	74.5	38.0	<=45	36.1	Not Reported	Baseline	BB,RASi,MRA,DIG,DIUR
190.	Gavazzi, 2015	Non-acute	Italy	2010-2012	174	72.0	27.0	<40	26.0	Records/Prescriptions	Baseline	BB,RASI,MRA,DIG
191.	Gentile, 2019	Non-acute	Italy	1990-2010	280	46.0	32.0	36-50	43.4	Not Reported	Baseline	BB,RASI,MRA,DIUR
192.	Grosu, 2011	Non-acute	Italy	2002-2003	106	65.0	25.0	<=35	26.0	Not Reported	Baseline	BB,RASI,MRA,DIG,DIUR,HYD/NIT
193.	Magri, 2014	Non-acute	Italy	2002-2011	1045	58.0	19.0	<=40	28.0	Not Reported	Baseline	BB,RASi,MRA,DIG,DIUR
194.	Magri, 2015	Non-acute	Italy	2001-2013	2976	59.7	13.6	<=40	28.9	Not Reported	Baseline	BB,RASi,MRA,DIG,DIUR

Obs.	Author (Year)	Settings	Country	Recruitment period (years)	Sample Size (n)	Mean Age	Female patients (%)	LVEF (%)		Data source for medication use	Type of medication data	Medications studied
								Eligibility	Mean			
195.	Merlo, 2011	Non-acute	Italy	1988-1997	242	43.0	26.0	<=35	31.0	Not Reported	Baseline	BB,RASI,DIG,DIUR
196.	Metra, 2010	Non-acute	Italy	NR	200	57.0	5.5	<=35	21.1	Not Reported	Baseline	RASI,MRA,DIG,DIUR
197.	Miniati, 2013	Non-acute	Italy	2000-2007	260	68.0	20.0	<50	32.0	Not Reported	Baseline	BB,RASI,MRA,DIUR
198.	Paolillo, 2017	Non-acute	Italy	NR	5242	61.0	19.0	<40	33.0	Not Reported	Baseline	BB,RASi,MRA,DIG,DIUR
199.	Santini, 2011	Non-acute	Italy	NR	1193	66.0	21.0	<=35	27.0	Not Reported	Baseline	BB,RASI,DIUR
200.	Scrutinio, 2012	Acute	Italy	2005-2009	275	67.0	18.5	<40	26.5	Records/Prescriptions	Discharge	BB,RASI,MRA
201.	Scrutinio, 2014	Acute	Italy	NR	445	62.0	15.3	<=30	22.9	Not Reported	Discharge	BB,MRA,DIUR
202.	Senni, 2014	Acute	Italy	2007-2009	2584	71.1	33.6	<50	31.6	Not Reported	Discharge	BB,RASI,MRA,DIUR
203.	Simioniuc, 2016	Non-acute	Italy	2004-2013	1137	66.3	22.2	<50	31.4	Not Reported	Baseline	BB,RASI,MRA,DIUR
204.	Stabile, 2018	Non-acute	Italy	2011-2013	930	71.6	26.2		29.1	Not Reported	Baseline	BB,RASI,MRA,IVA,DIUR
205.	Stolfo, 2015	Non-acute	Italy	1988-2009	470	45.0	30.0	<=35	30.0	Not Reported	Baseline	BB,RASI,DIG,DIUR
206.	Zecchin, 2012	Non-acute	Italy	1988-2006	287	43.0	25.0	<=35	29.0	Not Reported	Baseline	BB,RASI,DIG,DIUR
207.	Brunner-La Rocca, 2019	Non-acute	Netherlands	2013-2016	8360	72.3	36.1	<50		Not Reported	Baseline	BB,RASI,MRA,IVA,DIG,DIUR
208.	Flu, 2010	Non-acute	Netherlands	2002-2008	194	67.0	23.0	<=50		Not Reported	Baseline	BB,RASI,DIUR
209.	Kessing, 2014	Non-acute	Netherlands	2006-2008	238	66.9	22.0	<=40	33.5	Not Reported	Baseline	BB,RASI,DIUR
210.	Kessing, 2019	Non-acute	Netherlands	NR	460	66.2	25.0	<=40	31.8	Records/Prescriptions	Baseline	BB,RASI,DIUR
211.	Mastenbroek, 2016	Non-acute	Netherlands	2009-2011	139	65.7	30.0		24.9	Not Reported	Baseline	BB,RASI

Obs.	Author (Year)	Settings	Country	Recruitment period (years)	Sample Size (n)	Mean Age	Female patients (%)	LVEF (%)		Data source for medication use	Type of medication data	Medications studied
								Eligibility	Mean			
212.	Szymanski, 2011	Non-acute	Netherlands	NR	100	58.0	23.5	<45	28.0	Not Reported	Baseline	BB,RASI,MRA,DIUR
213.	Veenis, 2019	Non-acute	Netherlands	2013-2016	8351	72.3	36.4	<50	32.7	Not Reported	Baseline	BB,RASI,MRA,IVA,DIUR
214.	Broch, 2015	Non-acute	Norway	2008-2012	102	51.0	27.0	<40	26.0	Not Reported	Baseline	BB,RASi,MRA,DIG,DIUR
215.	Gasior, 2018	Acute	Poland	2009-2013	28080	68.7	34.1	<=40		Not Reported	Discharge	BB,RASI,DIUR
216.	Jankowska, 2014	Non-acute	Poland	2009	5563	67.0	37.0	<=45	36.0	Not Reported	Baseline	BB,RASi,MRA,DIG,DIUR
217.	Kaplon-Cieslicka, 2014	Non-acute	Poland	2009-2010	598	69.4	35.4		36.6	Records/Prescriptions	Baseline	BB,RASI,MRA
218.	Kaufmann, 2019	Non-acute	Poland	2009-2018	193	64.0	8.0		33.0	Not Reported	Baseline	BB,RASi,MRA,DIG,DIUR
219.	Kisiel, 2018	Non-acute	Poland	2006-2014	552	67.8	18.3		24.3	Not Reported	Baseline	BB,RASI,MRA,DIUR
220.	Migaj, 2018	Non-acute	Poland	2012-2013	653	63.4	.	<=45	29.9	Not Reported	Baseline	BB,RASI,MRA
221.	Opolski, 2017	Non-acute	Poland	2014	209	67.4	23.0	<=40	30.8	Records/Prescriptions	Baseline	BB,RASI,MRA,IVA,DIG,DIUR
222.	RafaÅ, Dankowski, 2018	Non-acute	Poland	NR	120	62.1	26.7	<45	37.5	Not Reported	Baseline	BB,RASI,IVA,DIUR
223.	Rywik, 2011	Non-acute	Poland	2004-2005	822	68.5	44.0	<45		Self Reported	Baseline	BB,RASi,MRA,DIG,DIUR
224.	Siennicka, 2016	Non-acute	Poland	2012-2013	758	64.0	21.0	<45	31.0	Not Reported	Baseline	BB,RASi,MRA,DIG,DIUR
225.	Jorge, 2013	Non-acute	Portugal	2003-2006	268	55.1	25.7		26.0	Self Reported, Records/Prescriptions	Baseline	BB,RASi,MRA,DIG,DIUR
226.	Laszczynska, 2017	Non-acute	Portugal	2000-2011	560	70.0	33.0	<50	26.0	Records/Prescriptions	Baseline	BB,RASI,MRA
227.	Marques, 2017	Acute	Portugal	2012	118	79.0	62.5	<40		Self Reported, Records/Prescriptions	Discharge	BB,RASI,MRA

Obs.	Author (Year)	Settings	Country	Recruitment period (years)	Sample Size (n)	Mean Age	Female patients (%)	LVEF (%)		Data source for medication use	Type of medication data	Medications studied
								Eligibility	Mean			
228.	Sargento, 2016	Non-acute	Portugal	2010-2012	138	77.9	33.3	<40	28.8	Records/Prescriptions	Baseline	BB,RASI,MRA,IVA,DIG,DIUR
229.	Sargento, 2017	Non-acute	Portugal	2010-2013	266	68.5	27.8	<40	28.0	Records/Prescriptions	Baseline	BB,RASI
230.	Toste, 2011	Non-acute	Portugal	NR	206	53.3	25.7	<40	27.7	Not Reported	Baseline	BB,RASI,MRA,DIUR
231.	A Bayes-Genas, 2015	Non-acute	Spain	2006-2013	480	67.5	22.7	<=35	26.0	Not Reported	Follow-up	BB,RASi,MRA,DIG,DIUR
232.	Agra Bermejo, 2018	Non-acute	Spain	2007-2014	116	66.0	11.0	<=40	29.1	Not Reported	Baseline	BB,RASI,MRA,DIUR
233.	Crespo-Leiro, 2015	Non-acute	Spain	2012-2013	1526	65.0	28.5	<40	35.0	Not Reported	Baseline	BB,RASI,MRA,IVA,DIG,DIUR
234.	Franco Pelaez, 2016	Non-acute	Spain	2008-2014	559	81.2	32.4	<=35	30.0	Not Reported	Baseline	BB,RASI,MRA,IVA,DIG,DIUR
235.	Franco, 2015	Acute	Spain	NR	238	76.7	38.0	<50		Not Reported	Discharge	BB,RASI
236.	Franco, 2019	Acute	Spain	2008-2016	385	76.9	36.6	<50		Not Reported	Discharge	BB,RASI,MRA,DIG
237.	Guisado-Espartero, 2018	Non-acute	Spain	NR	1089	79.3	38.7	<=49	35.3	Not Reported	Baseline	BB,RASI,MRA,IVA,DIG,DIUR
238.	Guzman, 2018	Non-acute	Spain	2006-2015	2364	70.7	71.4		34.6	Not Reported	Baseline	BB,RASI,MRA,IVA,DIG,DIUR
239.	Lopez-Azor, 2019	Non-acute	Spain	2016-2018	527	70.0	26.9		30.0	Not Reported	Baseline	BB,RASI,MRA
240.	Lupon, 2015	Non-acute	Spain	2006-2012	304	66.1	20.4	<40	28.0	Not Reported	Baseline	BB,RASI,MRA,IVA
241.	Martinez-Milla, 2019	Non-acute	Spain	2008-2014	390	82.6	37.7	<=35	27.9	Records/Prescriptions	Baseline	BB,RASI,MRA,IVA,DIG,DIUR
242.	Martinez-Selles, 2010	Acute	Spain	2001-2004	611	66.0	23.0	<40		Not Reported	Discharge	RASI,MRA,DIUR

Obs.	Author (Year)	Settings	Country	Recruitment period (years)	Sample Size (n)	Mean Age	Female patients (%)	LVEF (%)		Data source for medication use	Type of medication data	Medications studied
								Eligibility	Mean			
243.	Munoz, 2017	Non-acute	Spain	2009-2012	297	74.2	33.0	<50		Not Reported	Baseline	BB,RASI,DIUR
244.	Perez-Rodon, 2018	Non-acute	Spain	NR	107	66.0	26.0	<35	26.0	Not Reported	Baseline	BB,RASI,MRA,IVA,DIUR
245.	Pons, 2010	Non-acute	Spain	2001-2008	960	69.0	29.1		31.0	Not Reported	Follow-up	BB,RASi,MRA,DIG,DIUR
246.	Vicent, 2018	Non-acute	Spain	2016-2017	427	68.1	30.5		28.8	Not Reported	Baseline	BB,RASI,MRA
247.	Vicent, 2019	Non-acute	Spain	NR	427	68.1	29.5		28.8	Not Reported	Baseline	BB,RASI,MRA
248.	Vicent, 2019	Non-acute	Spain	2016-2017	427	68.1	30.5		28.9	Not Reported	Baseline	BB,RASI,MRA
249.	Vicent, 2019	Non-acute	Spain	2013-2014	810	69.4	26.0	<=50		Not Reported	Baseline	BB,RASI,MRA,DIG
250.	de Diego, 2018	Non-acute	Spain	NR	120	69.0	9.0	<=40	30.4	Not Reported	Baseline	BB,MRA,DIUR
251.	A Makubi, 2016	Non-acute	Sweden	2012-2013	1232	55.0	50.0	<50		Not Reported	Baseline	BB,RASi,MRA,DIG,DIUR
252.	Bakos, 2017	Non-acute	Sweden	2011-2014	211	71.6	20.0		26.5	Not Reported	Baseline	BB,RASI,MRA,DIG,DIUR
253.	Barywani, 2015	Non-acute	Sweden	2000-2008	324	82.9	33.9	<=40	32.9	Records/Prescriptions	Baseline	MRA,DIUR
254.	Desta, 2016	Acute	Sweden	1998-2010	30958	73.3	36.1	<49		Not Reported	Discharge	BB,RASI
255.	Fu, 2017	Non-acute	Sweden	2014-2016	612	69.2	23.0	<50	32.7	Not Reported	Baseline	BB,RASI,MRA,IVA,DIG,DIUR
256.	Jan StÃ¥lhammar, 2012	Non-acute	Sweden	2005-2006	252	74.1	31.0	<=40		Records/Prescriptions	Follow-up	BB,RASi,MRA,DIG,DIUR
257.	Jonsson, 2018	Non-acute	Sweden	2010-2016	812	75.0	31.0	<=40	33.0	Records/Prescriptions	Baseline	BB,RASI,DIG,DIUR
258.	Kontogeorgos, 2017	Non-acute	Sweden	2007-2008	189	78.0	36.5	<50	34.0	Records/Prescriptions	Baseline	BB,RASi,MRA,DIG,DIUR
259.	Li, 2015	Non-acute	Sweden	2000-2013	18858	72.5	29.7	<40		Not Reported	Baseline	BB,RASi,MRA,DIG,DIUR

Obs.	Author (Year)	Settings	Country	Recruitment period (years)	Sample Size (n)	Mean Age	Female patients (%)	LVEF (%)		Data source for medication use	Type of medication data	Medications studied
								Eligibility	Mean			
260.	Lund, 2018	Non-acute	Sweden	2000-2014	17193	71.9	27.6	<40		Not Reported	Baseline	BB,RASI,MRA
261.	Reitan, 2015	Non-acute	Sweden	1999-2012	705	69.6	16.5		25.0	Records/Prescriptions	Baseline	BB,RASI,DIG,DIUR
262.	Savarese, 2015	Non-acute	Sweden	2000-2012	22947	72.2	28.8	<40		Not Reported	Baseline	BB,RASi,MRA,DIG,DIUR
263.	Altunbas, 2016	Acute	Turkey	2007-2011	388	70.3	33.6	<40	29.5	Records/Prescriptions	Discharge	BB,RASI,MRA,DIG
264.	Ege, 2012	Acute	Turkey	NR	959	61.0	28.3	<45	32.7	Records/Prescriptions	Discharge	BB,RASi,MRA,DIG,DIUR
265.	Ekmekci, 2016	Non-acute	Turkey	2013-2014	293	46.2	19.7		26.0	Records/Prescriptions	Baseline	BB,RASi,MRA,DIG,DIUR
266.	Kozdag, 2012	Non-acute	Turkey	2003-2009	580	63.0	35.7	<45	26.0	Not Reported	Baseline	BB,RASI
267.	Ozlek, 2019	Non-acute	Turkey	2018	246	68.0	42.3	40-49	45.0	Self Reported	Baseline	BB,RASI,MRA,IVA,DIG,DIUR
268.	Tokatli, 2015	Non-acute	Turkey	2004-2013	630	66.0	36.7	<45	25.2	Not Reported	Baseline	BB,RASI,MRA,DIG
269.	Yucel, 2015	Non-acute	Turkey	NR	392	60.0	30.9	<=40	31.3	Not Reported	Baseline	BB,RASi,MRA,DIG,DIUR
270.	Zorlu, 2012	Non-acute	Turkey	2009-2011	150	68.0	39.3	<45	31.6	Not Reported	Baseline	BB,RASI
271.	A Bayes-Genis, 2012	Non-acute	Germany, Spain	2006-2010	891	70.2	28.4		26.9	Not Reported	Follow-up	BB,RASi,MRA,DIG,DIUR
272.	Frohlich, 2017	Non-acute	Norway, Germany, UK	NR	6010	67	27.2	<45	33	Not Reported	Baseline	RASI,MRA,DIUR
273.	Frohlich, 2018	Non-acute	Norway, Germany, UK	NR	4723	67	23.6	<45	30	Not Reported	Baseline	BB,RASI,MRA,DIUR
274.	Frohlich, 2019	Non-acute	Norway, Germany, UK	1995-2015	9166	66.8	24.9	<=49	30.6	Not Reported	Baseline	BB,RASI,MRA,DIUR

Obs.	Author (Year)	Settings	Country	Recruitment period (years)	Sample Size (n)	Mean Age	Female patients (%)	LVEF (%)		Data source for medication use	Type of medication data	Medications studied
								Eligibility	Mean			
275.	Kapelios, 2019	Non-acute	Austria, Bosnia and Herzegovina, Bulgaria, Czech Republic, Egypt, France, Greece, Hungary, Israel, Italy, Latvia, Lithuania, Poland, Portugal, Romania, Serbia, Slovakia, Slovenia, Spain, Sweden, Turkey	2011-2013	5443	63.8	22	<=40		Not Reported	Baseline	BB,RASI,MRA,DIUR
276.	Lopatin, 2018	Non-acute	Armenia, Azerbaijan, Belarus, Georgia, Kazakhstan, Russia, Ukraine, Uzbekistan	2015-2016	370	61.6	26	<40	29.4	Not Reported	Baseline	RASI,MRA,IVA,DIUR

Obs.	Author (Year)	Settings	Country	Recruitment period (years)	Sample Size (n)	Mean Age	Female patients (%)	LVEF (%)		Data source for medication use	Type of medication data	Medications studied
								Eligibility	Mean			
277.	Maggioni, 2013	Non-acute	Bulgaria, Czech Republic, Hungary, Poland, Romania, Slovakia, Latvia, Lithuania, Sweden, Bosnia and Herzegovina, Greece, Italy, Portugal, Serbia, Slovenia, Spain, Turkey, Austria, France, Israel, Egypt	2011-2013	4792	68	32.2	<=45		Not Reported	Baseline	BB,RASI,MRA,IVA,DIG,DIUR
278.	Scrutinio, 2015	Acute	Italy, Europe	2006-2014	701	63	16.3	<=30	23.1	Records/Prescriptions	Discharge	BB,MRA,DIUR
279.	Tromp, 2018	Non-acute	Netherland, Germany, UK, Norway, Greece, Italy, Poland, France	NR	2615	69.2	25.2	<=40	29	Not Reported	Baseline	BB,RASI,MRA,DIUR
South-East Asia												
280.	Chopra, 2019	Non-acute	India	2014-2017	5590	59.1	17.0	<=40	30.0	Not Reported	Baseline	BB,RASI,IVA,DIG,DIUR

Obs.	Author (Year)	Settings	Country	Recruitment period (years)	Sample Size (n)	Mean Age	Female patients (%)	LVEF (%)		Data source for medication use	Type of medication data	Medications studied
								Eligibility	Mean			
281.	Harikrishnan, 2015	Non-acute	India	2013	894	61.2	31.0		.	Not Reported	Baseline	BB,RASi,MRA,DIG,DIUR
282.	Pokharel, 2016	Non-acute	India	2008-2014	15870	56.0	22.9	<40	.	Records/Prescriptions	Baseline	BB,RASI
283.	Ahn, 2019	Non-acute	Korea	2011-2014	2769	66.4	39.0	<40	26.8	Not Reported	Baseline	BB,RASI,MRA
284.	Choi, 2018	Non-acute	Korea	2010-2012	157	59.0	27.0	<40	28.0	Not Reported	Baseline	RASI,MRA,DIG,DIUR
285.	Gwag, 2018	Acute	Korea	2011-2014	2577	67.8	39.4	<50	29.6	Not Reported	Discharge	BB,RASI,MRA
286.	Son, 2011	Non-acute	Korea	NR	232	65.0	28.9	<40	29.5	Records/Prescriptions	Baseline	BB,RASi,MRA,DIG,DIUR
287.	Yoo, 2014	Acute	Korea	2009	1297	69.0	43.7	<45	29.7	Not Reported	Discharge	BB,RASI,MRA
288.	Youn, 2012	Acute	Korea	2004-2009	1527	69.1	44.1	<40	28.7	Not Reported	Discharge	BB,RASI,MRA
289.	Silavanich, 2019	Non-acute	Thailand	2015-2017	180	63.1	38.9	<=40	28.2	Self Reported, Records/Prescriptions	Baseline	BB,RASI,MRA,IVA,DIG,DIUR, HYD/NIT
Western Pacific												
290.	Alison Mudge, 2010	Acute	Australia	2000-2002	416	76.6	53.1		26.1	Not Reported	Discharge	BB,RASI
291.	Ne, 2019	Non-acute	Australia	2001-2012	177	76.5	43.5	<50		Not Reported	Baseline	BB,RASI,DIUR
292.	Newton, 2016	Acute	Australia	2013	433	75.0	31.0	<50		Not Reported	Discharge	BB,RASi,MRA,DIG,DIUR
293.	V Khalil, 2017	Acute	Australia	2013	618	78.9	51.8			Records/Prescriptions	Discharge	BB,RASi,MRA,DIG,DIUR
294.	Wai, 2012	Non-acute	Australia	NR	125	70.9	23.2	<50		Not Reported	Baseline	RASI,MRA,DIUR
295.	Wong, 2010	Non-acute	Australia	1994-2004	564	75.0	38.5	<=45		Records/Prescriptions	Baseline	BB,RASI,MRA,DIG
296.	Jackson, 2018	Non-acute	China	2016	677	66.0	45.0	<50		Not Reported	Baseline	BB,RASI,MRA

Obs.	Author (Year)	Settings	Country	Recruitment period (years)	Sample Size (n)	Mean Age	Female patients (%)	LVEF (%)		Data source for medication use	Type of medication data	Medications studied
								Eligibility	Mean			
297.	Kang, 2015	Non-acute	China	NR	260	71.1	40.8	<50	39.2	Self Reported	Baseline	BB,RASi,MRA,DIG,DIUR
298.	Peng, 2018	Non-acute	China	2005-2014	993	59.0	16.4	<=40	37.5	Not Reported	Baseline	BB,RASi,MRA,DIG,DIUR
299.	SHI Chuan, 2010	Non-acute	China	1995-2009	1119	65.0	28.7	<45	37.8	Records/Prescriptions	Baseline	BB,RASI,DIG,DIUR
300.	Xin, 2019	Non-acute	China	2014-2015	197	64.0	56.3	40-49		Not Reported	Baseline	BB,RASi,MRA,DIG,DIUR
301.	Zhang, 2017	Acute	China	2012-2015	4482	60.0	30.2	<45		Not Reported	Discharge	BB,RASI,MRA,DIUR
302.	Akita, 2017	Acute	Japan	2005-2014	773	70.8	36.0	<45	32.2	Not Reported	Discharge	BB,RASI,MRA,DIG,DIUR
303.	Goto, 2013	Acute	Japan	NR	527	63.5	26.9	<50	29.4	Not Reported	Discharge	BB,RASi,MRA,DIG,DIUR
304.	Ikeda, 2016	Non-acute	Japan	1996-2011	207	55.0	23.0	<45	31.0	Not Reported	Baseline	BB,RASI,MRA,DIUR
305.	Ito, 2019	Acute	Japan	2014-2016	357	68.8	28.6	<=49	27.7	Not Reported	Discharge	BB,RASI,MRA,DIUR
306.	Kato, 2013	Non-acute	Japan	2002-2009	227	62.4	24.0	<50	32.5	Records/Prescriptions	Baseline	RASI,MRA,DIG,DIUR
307.	Shiga, 2019	Non-acute	Japan	2013-2014	707	73.9	34.9	<50	35.6	Not Reported	Baseline	BB,RASi,MRA,DIG,DIUR
308.	Takada, 2014	Non-acute	Japan	NR	885	66.6	26.0	<=50	38.8	Not Reported	Baseline	BB,MRA,DIUR
309.	Tsuchihashi-Makaya 2010	Acute	Japan	2004-2005	947	66.3	27.8	<40	27.1	Not Reported	Discharge	BB,RASi,MRA,DIG,DIUR
310.	Grainger, 2013	Non-acute	New Zealand	1997-2011	451	74.8	31.0	<36	.	Records/Prescriptions	Baseline	BB,RASI,MRA
311.	Hoong, 2015	Acute	Singapore	2009	244	66.0	34.8	<=50	.	Records/Prescriptions	Discharge	BB,RASi,MRA,DIG,DIUR
312.	CH Wang, 2018	Non-acute	Taiwan	2011-2014	599	60.9	32.9	<50	33.9	Records/Prescriptions	Baseline	BB,RASI,DIUR
313.	Chang, 2017	Non-acute	Taiwan	2013-2014	1509	63.9	27.6	<40	28.2	Not Reported	Baseline	BB,RASI,MRA

Obs.	Author (Year)	Settings	Country	Recruitment period (years)	Sample Size (n)	Mean Age	Female patients (%)	LVEF (%)		Data source for medication use	Type of medication data	Medications studied
								Eligibility	Mean			
314.	Chang, 2019	Acute	Taiwan	2013-2014	393	69.2	28.0	<40	28.8	Not Reported	Discharge	BB,RASi,MRA,DIG,DIUR
315.	Chang, 2019	Non-acute	Taiwan	2016-2017	932	61.7	25.6	<40	27.2	Not Reported	Baseline	BB,RASI,MRA,ARNI,IVA
316.	H Altay, 2012	Non-acute	Taiwan	2011-2014	599	60.9	32.9	<50	33.9	Records/Prescriptions ,physically Examining	Baseline	BB,RASI,DIUR
317.	Lin, 2016	Non-acute	Taiwan	2004-2009	128	50.3	27.3		23.4	Not Reported	Baseline	BB,RASI,DIUR
318.	Shih-Hung Hsiao, 2018	Non-acute	Taiwan	2008-2016	765	60.4	22.6	<35	29.2	Not Reported	Baseline	BB,RASI,MRA,DIUR
319.	Shih-Hung Hsiao, 2018	Non-acute	Taiwan	2008-2016	985	60.6	22.5	<35	29.4	Not Reported	Baseline	BB,RASI,MRA,DIUR
320.	Sung, 2018	Acute	Taiwan	2003-2012	423	84.5	21.7	<50	34.7	Records/Prescriptions	Discharge	BB,RASi,MRA,DIG,DIUR
321.	Yeh, 2016	Non-acute	Taiwan	2010	428	64.0	29.0	<=35	26.9	Not Reported	Baseline	BB,RASI,DIG,DIUR
Multiple Regions												
322.	Agostoni, 2010	Non-acute	Italy, US	2001-2009	572	62.4	18.2	<=50	35.9	Not Reported	Baseline	BB,RASI,MRA,DIUR
323.	Arnold, 2019	Non-acute	Indonesia, Philippines, India, China, Thailand, Malaysia	2010-2016	1160	59.8	21.7	<40	28.1	Records/Prescriptions	Baseline	BB,RASI,SGLT,DIUR
324.	Arnold, 2019	Non-acute	Singapore, Hongkong, Taiwan, South Korea, Japan	2010-2016	1075	63.2	21	<40	27	Records/Prescriptions	Baseline	BB,RASI,SGLT,DIUR

Obs.	Author (Year)	Settings	Country	Recruitment period (years)	Sample Size (n)	Mean Age	Female patients (%)	LVEF (%)		Data source for medication use	Type of medication data	Medications studied
								Eligibility	Mean			
325.	Atherton, 2012	Acute	Australia, Malaysia, Singapore, Philippines, England, US, Hongkong, Taiwan, Thailand, Indonesia	2006-2008	10171	66	43	<40	.	Records/Prescriptions	Discharge	BB,RASi,MRA,DIG,DIUR
326.	Dokainish, 2016	Non-acute	Argentina, Chile, China, Colombia, Ecuador, Egypt, India, Malaysia, Mozambique, Nigeria, Philippines, Qatar, Saudi Arabia, South Africa, Sudan, Uganda	2012-2014	3306	57.7	31.7	<50		Not Reported	Baseline	BB,RASi,MRA,DIG,DIUR

327.	Komajda, 2016	Non-acute	Armenia, Australia, Austria, Azerbaijan, Bahrain, Belarus, Brunei, Canada, China, Denmark, Ecuador, Egypt, Georgia, Germany, Greece, Hungary, Ireland, Jordan, Kazakhstan, Korea, Kuwait, Lebanon, Lithuania, Malaysia, Morocco, Poland, Portugal, Qatar, Romania, Russia, Slovakia, Spain, Thailand, Turkey, UK, UAE	2013-2014	7092	63.1	26	<40	31.9	Not Reported	Baseline	BB,RASI,MRA,IVA,DIG,DIUR

328.	Komajda, 2017	Non-acute	Armenia, Australia, Austria, Azerbaijan, Bahrain, Belarus, Brunei, Canada, China, Denmark, Ecuador, Egypt, Georgia, Germany, Greece, Hungary, Ireland, Jordan, Kazakhstan, Korea, Kuwait, Lebanon, Lithuania, Malaysia, Morocco, Poland, Portugal, Qatar, Romania, Russia, Slovakia, Spain, Thailand, Turkey, UK, UAE	2013-2014	6669	63	25.8	<=40	31.9	Not Reported	Baseline	BB,RASi,MRA,DIG,DIUR

329.	Komajda, 2019	Non-acute	Armenia, Australia, Austria, Azerbaijan, Bahrain, Belarus, Brunei, Canada, China, Denmark, Ecuador, Egypt, Georgia, Germany, Greece, Hungary, Ireland, Jordan, Kazakhstan, Korea, Kuwait, Lebanon, Lithuania, Malaysia, Morocco, Poland, Portugal, Qatar, Romania, Russia, Slovakia, Spain, Thailand, Turkey, UK, UAE	2013-2014	7315	63.2	26.2	<=40	33	Not Reported	Baseline	BB,RASI,MRA,IVA

Obs.	Author (Year)	Settings	Country	Recruitment period (years)	Sample Size (n)	Mean Age	Female patients (%)	LVEF (%)		Data source for medication use	Type of medication data	Medications studied
								Eligibility	Mean			
330.	Kubota, 2018	Non-acute	China, Hongkong, India, Indonesia, Japan, South Korea, Malaysia, Philippines, Singapore, Taiwan, Thailand	2010-2015	5232	59.7	21.9	<=40	27.3	Not Reported	Baseline	BB,RASI,MRA,DIUR
331.	Magana-Serrano, 2011	Non-acute	Chile, Colombia, Mexico, Iran, Lebanon, Saudi Arabia, UAE, Algeria, Egypt, Tunisia	NR	699	62	28	<45	32	Not Reported	Baseline	BB,RASi,MRA,DIG,DIUR
332.	Tang, 2013	Non-acute	US, New Zealand	2001-2003	138	58	23	<=35	26	Not Reported	Baseline	BB,RASI,MRA,DIUR

Obs.	Author (Year)	Settings	Country	Recruitment period (years)	Sample Size (n)	Mean Age	Female patients (%)	LVEF (%)		Data source for medication use	Type of medication data	Medications studied
								Eligibility	Mean			
333.	Teng, 2018	Non-acute	China, Hongkong, India, Indonesia, Japan, South Korea, Malaysia, Philippines, Singapore, Taiwan, Thailand	2012-2015	5276	59.6	23	<=40	27	Not Reported	Baseline	
334.	Yoo, 2015	Non-acute	South Korea, Taiwan, China	NR	1470	66.3	33.5	<45	30.9	Not Reported	Baseline	BB,RASi,MRA,DIG,DIUR

BB: Beta blockers; RASi: Renin-Angiotensin System inhibitors (includes angiotensin converting enzyme inhibitor and/or angiotensin receptor II blocker); MRA: Mineralocorticoid Receptor Antagonists; DIUR: Diuretics (includes loop and thiazide); DIG: Digoxin; IVA: Ivabradine; ARNI: Angiotensin Receptor Neprilysin inhibitor; SGLT: Sodium-glucose transport protein 2 inhibitors; HYD/NIT: Hydralazine/Nitrate; LVEF: Left ventricular ejection fraction
*Median/Range

Table S5. Characteristics of registry-based studies

	Author, Year	Setting	Registry Name	Country	Recruitment period	Sample Size (n)	Mean Age	Female (%)	LVEF (%)		Data source for medication use	Type of medication data	Medications studied
									Eligibility	Mean			
1	A Makubi, 2016	Non-acute	TAHEF	Tanzania	2012-2013	411	55.0	50.0	<50	.	Not reported	Baseline	BB,RASi,MRA,DIG,DIUR
2	Ghimire, 2019	Non-acute	Not reported	Canada	2008-2016	3124	66.8	27.6	<=40	28.7	Not reported	Baseline	BB,RASI,MRA,ARNI,DIG,DIUR
3	El-Chami, 2010	Non-acute	ADVACENT	US	2003-2004	25025	66.3	28.5	<=40	31.1	Self-reported, Records/prescriptions	Baseline	BB,RASI
4	Lam, 2017	Acute	ALABAMA-HF	US	1998-2001	1874	73.6	44.4	<=35	25.1	Not reported	Discharge	BB,RASi,MRA,DIG,DIUR
5	Hernandez, 2012	Acute	AMERICAN HEART ASSOCIATION-GET WITH THE GUIDELINES-HEART FAILURE	US	2005-2009	5887	77.6	35.7	<=35	25.4	Not reported	Discharge	BB,RASI,DIG,DIUR
6	DeVore, 2018	Non-acute	CHAMP-HF	US	2015-2017	4216	66.1	29.8	<=40	29.3	Not reported	Baseline	BB,RASI,MRA,ARNI,IVA,HYD/NIT
7	Arnold, 2019	Non-acute	DIABETES COLLABORATIVE REGISTRY	US	2013-2016	28877	70.0	30.0	<40	27.7	Not reported	Baseline	BB,RASI,DIUR
8	Luo, 2018	Acute	GET WITH THE GUIDELINE	US	2016	3738	70.0	35.7	<=40	25.0	Not reported	Discharge	BB,RASI,MRA
9	Luo, 2019	Acute	GET WITH THE GUIDELINE	US	2015-2016	16674	69.0	35.4	<=40	25.0	Records/prescriptions	Discharge	BB,RASI,MRA,ARNI
10	Pandey, 2016	Acute	GET WITH THE GUIDELINE	US	2005-2014	111846	69.9	36.0	<40	25.1	Records/prescriptions	Discharge	BB,RASI
11	Patel, 2016	Non-acute	GET WITH THE GUIDELINE	US	2005-2014	117761	68.9	36.3		.	Not reported	Baseline	BB,RASi,MRA,DIG,DIUR
12	Shreibati, 2016	Acute	GET WITH THE GUIDELINE	US	2009-2015	1458	60.0	29.6	<=25	.	Not reported	Discharge	BB,RASI,MRA,HYD/NIT
13	Schneider, 2014	Non-acute	ICD	US	2006-2008	45392	69.3	31.5	<=35	.	Not reported	Baseline	BB,RASI
14	Wilcox, 2012	Non-acute	IMPROVE HF	US	Not reporte	3994	66.6	29.7	<=35	25.8	Records/prescriptions	Baseline	BB,RASI,MRA,DIG

**15**	Albert, 2010	Non-acute	IMPROVE-HF	US	2005-2007	15381	68.7	29.0	<=35	25.4	Records/prescriptions	Baseline	BB,RASI,MRA
**16**	Fonarow, 2011	Non-acute	IMPROVE-HF	US	Not reporte	11621	70.0	28.9	<=35	.	Records/prescriptions	Baseline	BB,RASI,MRA
**17**	Roth, 2016	Non-acute	NCDR-ICD	US	2007-2011	19773	74.9	35.4	<=40	25.6	Records/prescriptions	Baseline	BB,RASI
**18**	Allen, 2018	Non-acute	NCDR-PINNACLE	US	2008-2014	75107	68.6	30.9	<40	.	Records/prescriptions	Baseline	BB,RASI,DIUR
**19**	Jehu S. Mathew, 2017	Non-acute	NCDR-PINNACLE	US	2013-2014	36786	73.2	27.1	<40	30.1	Records/prescriptions	Baseline	BB,DIG
**20**	Mathew, 2017	Non-acute	NCDR-PINNACLE	US	2013-2014	36786	73.2	27.1	<40	30.1	Records/prescriptions	Baseline	BB,DIG
**21**	Wang, 2016	Non-acute	NEOLITH	US	2005-2015	102	57.9	46.0	<=35	21.5	Not reported	Discharge	BB,RASi,MRA,DIG,DIUR
**22**	Luzum, 2019	Non-acute	Not reported	US	2007-2015	951	68.3	35.9	<50	34.7	Records/prescriptions	Baseline	BB,RASI
**23**	Bayoumi, 2019	Acute	OPTIMIZE-HF	US	2003-2004	6986	75.9	40.2	<=35	24.9	Records/prescriptions	Discharge	BB,RASi,MRA,DIG,DIUR
**24**	DeVore, 2016	Acute	OPTIMIZE-HF	US	2003-2004	10696	68.9	41.4	<40	.	Not reported	Discharge	BB,RASI,MRA
**25**	Qamer, 2019	Acute	OPTIMIZE-HF	US	2003-2004	8401	76.0	43.7	<=45	29.4	Not reported	Discharge	BB,RASI,MRA,DIUR
**26**	Butler, 2019	Non-acute	PINNACLE	US	2011-2014	11064	69.3	35.2	<50	32.5	Records/prescriptions	Baseline	BB,RASI,MRA
**27**	Fleming, 2016	Non-acute	PINNACLE	US	2009-2012	26658	68.5	42.9	<40	.	Records/prescriptions	Baseline	BB,RASI
**28**	Wu, 2013	Acute	UNIVERSITY OF MICHIGAN ACS	US	1999-2007	461	64.1	33.0	<40	33.8	Not reported	Discharge	BB,RASI,DIG,DIUR
**29**	Abi Khalil, 2018	Acute	GULF-SAFE	Bahrain, Kuwait, Oman, Qatar, UAE, Yemen	2009-2010	334	62.0	34.0	<40	31.0	Not reported	Discharge	BB,RASI,DIG,DIUR
**30**	Abi Khalil, 2017	Non-acute	GULF-CARE	Oman, Saudi Arabia, UAE, Qatar, Bahrain, Yemen, Kuwait	2012	1278	60.8	27.4	<40	27.0	Not reported	Baseline	RASi,MRA,IVA,DIUR
**31**	AlShamiri, 2018	Acute	HEARTS	Saudi Arabia	2009-2010	387	61.6	20.4	<=40	.	Not reported	Discharge	BB,RASI,DIG,DIUR
**32**	Lopatin, 2018	Non-acute	OPTIMIZE-HF	Armenia, Azerbaijan,	2015-2016	370	61.6	26.0	<40	29.4	Not reported	Baseline	RASI,MRA,IVA,DIUR

				Belarus, Georgia, Kazakhstan, Russia, UKraine, Uzbekistan									
**33**	Poelzl, 2014	Non-acute	HIR-AUSTRIA	Austria	2006-2010	1014	65.0	27.4	<=40	.	Not reported	Baseline	BB,RASi,MRA,DIG,DIUR
**34**	Kapelios, 2019	Non-acute	ESC-EORP-HFA	Austria, Bosniaherzegovina, Bulgaria, Czechrepublic, Egypt, France, Greece, Hungary, Israel, Italy, Latvia, Lithuania, Poland, Portugal, Romania, Serbia, Slovakia, Slovenia, Spain, Sweden, Turkey	2011-2013	5443	63.8	22.0	<=40	.	Not reported	Baseline	BB,RASI,MRA,DIUR
**35**	De Sutter, 2015	Acute	BIO-HF	Belgium	2008-2012	543	75.3	34.7	<=49	30.7	Not reported	Discharge	BB,RASI,MRA
**36**	Houard, 2019	Non-acute	NOT REPORTED	Belgium	2002-2015	266	60.0	29.0	<=35	23.0	Not reported	Baseline	BB,RASi,MRA,DIG,DIUR
**37**	Vandenberk, 2016	Non-acute	NOT REPORTED	Belgium	1996-2014	244	56.6	26.2		27.6	Not reported	Baseline	BB,RASI,DIG,DIUR
**38**	Maggioni, 2013	Non-acute	ESC-HFLTR	Bulgaria, Czech Republic, Hungary,	2011-2013	4792	68.0	32.2	<=45	.	Not reported	Baseline	BB,RASI,MRA,IVA,DIG,DIUR

				Poland,<br>Romania,<br>Slovakia,<br>Latvia,<br>Lithuania,<br>Sweden,<br>Bosnia<br>Erzegovina,<br>Greece, Italy,<br>Portugal,<br>Serbia,<br>Slovenia,<br>Spain,<br>Turkey,<br>Austria,<br>France,<br>Israel, Egypt									
**39**	Gjesing, 2013	Non-acute	DANISH REGISTRY OF MEDICINAL PRODUCT STATISTICS	Denmark	2002-2009	8792	69.0	28.0	<45	30.0	Records/prescriptions	Baseline	BB,RASI,MRA
**40**	Anne Nakano1, 2019	Non-acute	DANISH-HF	Denmark	2003-2010	17758	70.9	36.4	<=40	.	Records/prescriptions	Baseline	BB,RASI
**41**	Pasternak, 2014	Non-acute	DANISH-HF	Denmark	2003-2012	11664	69.2	29.3	<=40	.	Not reported	Baseline	BB,RASI,MRA,DIUR
**42**	Bitar, 2019	Acute	EPICAL2	France	2011-2012	354	.	35.0	<40	.	Records/prescriptions	Discharge	BB,RASI,MRA
**43**	Busson, 2018	Acute	EPICAL2	France	2011-2012	624	73.8	35.4	<40	28.2	Records/prescriptions	Discharge	BB,RASI,MRA,DIUR
**44**	von Scheidt, 2014	Non-acute	EVITA-HF	Germany	2009-2011	1803	70.0	24.3	<=40	30.0	Not reported	Baseline	BB,RASI,MRA,IVA,DIG,DIUR
**45**	Frankenstein, 2010	Non-acute	HELUMA	Germany	1994-2007	3292	60.8	22.3		28.2	Records/prescriptions	Baseline	BB,RASi,MRA,DIG,DIUR
**46**	Stabile, 2018	Non-acute	CRT-MORE	Italy	2011-2013	930	71.6	26.2		29.1	Not reported	Baseline	BB,RASI,MRA,IVA,DIUR
**47**	Gentile, 2019	Non-acute	HEART MUSCLE DISEASE	Italy	1990-2010	280	46.0	32.0	36-50	43.4	Not reported	Baseline	BB,RASI,MRA,DIUR

			REGISTRY OF TRIESTE										
**48**	Merlo, 2011	Non-acute	HEART MUSCLE DISEASE REGISTRY OF TRIESTE	Italy	1988-1997	242	43.0	26.0	<=35	31.0	Not reported	Baseline	BB,RASI,DIG,DIUR
**49**	Stolfo, 2015	Non-acute	HEART MUSCLE DISEASE REGISTRY OF TRIESTE	Italy	1988-2009	470	45.0	30.0	<=35	30.0	Not reported	Baseline	BB,RASI,DIG,DIUR
**50**	Zecchin, 2012	Non-acute	HEART MUSCLE DISEASE REGISTRY OF TRIESTE	Italy	1988-2006	287	43.0	25.0	<=35	29.0	Not reported	Baseline	BB,RASI,DIG,DIUR
**51**	Senni, 2014	Acute	IN-HF	Italy	2007-2009	2584	71.1	33.6	<50	31.6	Not reported	Discharge	BB,RASI,MRA,DIUR
**52**	Boriani, 2012	Non-acute	INSYNC ICD ITALIAN	Italy	Not reporte	659	66.3	9.9	<=35	26.0	Not reported	Baseline	BB,RASI,DIUR
**53**	Gavazzi, 2015	Non-acute	Not Reported	Italy	2010-2012	174	72.0	27.0	<40	26.0	Records/prescriptions	Baseline	BB,RASI,MRA,DIG
**54**	Aleksova, 2011	Non-acute	HEART MUSCLE DISEASE REGISTRY OF TRIESTE	Italy	1988-2007	491	45.0	25.0		30.0	Not reported	Baseline	BB,RASI,DIUR
**55**	Tromp, 2018	Non-acute	BIOSTAT-CHF	Netherland, Germany, UK, Norway, Greece, Italy, Poland, France	Not reporte	2615	69.2	25.2	<=40	29.0	Not reported	Baseline	BB,RASI,MRA,DIUR
**56**	Brunner-La Rocca, 2019	Non-acute	CHECK-HF	Netherlands	2013-2016	8360	72.3	36.1	<50	.	Not reported	Baseline	BB,RASI,MRA,IVA,DIG,DIUR
**57**	Veenis, 2019	Non-acute	CHECK-HF	Netherlands	2013-2016	8351	72.3	36.4	<50	32.7	Not reported	Baseline	BB,RASI,MRA,IVA,DIUR
**58**	Frohlich, 2017	Non-acute	NORWEGIAN-HF, HEIDELBERG-HF, HULL-HF	Norway, Germany, UK	Not reporte	6010	67.0	27.2	<45	33.0	Not reported	Baseline	RASI,MRA,DIUR

**59**	Frohlich, 2018	Non-acute	NORWEGIAN-HF, HEIDELBERG-HF, HULL-HF	Norway, Germany, UK	Not reporte	4723	67.0	23.6	<45	30.0	Not reported	Baseline	BB,RASI,MRA,DIUR
**60**	Frohlich, 2019	Non-acute	NORWEGIAN-HF, HEIDELBERG-HF, HULL-HF	Norway, Germany, UK	1995-2015	9166	66.8	24.9	<=49	30.6	Not reported	Baseline	BB,RASI,MRA,DIUR
**61**	Jankowska, 2014	Non-acute	DATA-HELP	Poland	2009	5563	67.0	37.0	<=45	36.0	Not reported	Baseline	BB,RASi,MRA,DIG,DIUR
**62**	Gasior, 2018	Acute	PL-ACS, AMI-PL	Poland	2009-2013	28080	68.7	34.1	<=40	.	Not reported	Discharge	BB,RASI,DIUR
**63**	Opolski, 2017	Non-acute	QUALIFY	Poland	2014	209	67.4	23.0	<=40	30.8	Records/prescriptions	Baseline	BB,RASI,MRA,IVA,DIG,DIUR
**64**	Agra Bermejo, 2018	Non-acute	CARDIOCHUS-CHOP	Spain	2007-2014	116	66.0	11.0	<=40	29.1	Not reported	Baseline	BB,RASI,MRA,DIUR
**65**	Martinez-Selles, 2010	Acute	CHULAPA	Spain	2001-2004	611	66.0	23.0	<40	.	Not reported	Discharge	RASI,MRA,DIUR
**66**	Crespo-Leiro, 2015	Non-acute	ESC HEART FAILURE LONG-TERM	Spain	2012-2013	1526	65.0	28.5	<40	35.0	Not reported	Baseline	BB,RASI,MRA,IVA,DIG,DIUR
**67**	Vicent, 2019	Non-acute	Not reported	Spain	Not reporte	427	68.1	29.5		28.8	Not reported	Baseline	BB,RASI,MRA
**68**	Vicent, 2019	Non-acute	Not reported	Spain	2016-2017	427	68.1	30.5		28.9	Not reported	Baseline	BB,RASI,MRA
**69**	Vicent, 2019	Non-acute	Not reported	Spain	2013-2014	810	69.4	26.0	<=50	.	Not reported	Baseline	BB,RASI,MRA,DIG
**70**	Guisado-Espartero, 2018	Non-acute	RICA	Spain	Not reporte	1089	79.3	38.7	<=49	35.3	Not reported	Baseline	BB,RASI,MRA,IVA,DIG,DIUR
**71**	Franco, 2015	Acute	SPANISH NATIONAL REGISTRY ON HF	Spain	Not reporte	238	76.7	38.0	<50	.	Not reported	Discharge	BB,RASI
**72**	Franco, 2019	Acute	SPANISH REGISTRY OF ACUTE HEART FAILURE	Spain	2008-2016	385	76.9	36.6	<50	.	Not reported	Discharge	BB,RASI,MRA,DIG
**73**	Vicent, 2018	Non-acute	SUMA	Spain	2016-2017	427	68.1	30.5		28.8	Not reported	Baseline	BB,RASI,MRA
**74**	Desta, 2016	Acute	SWEDEHEART	Sweden	1998-2010	30958	73.3	36.1	<49	.	Not reported	Discharge	BB,RASI

**75**	Jan StÃ¥lhammar, 2012	Non-acute	SWEDISH PATIENT REGISTRY, SWEDISH PRESCRIPTION REGISTRY, SWEDISH CAUSE OF DEATH REGISTRY	Sweden	2005-2006	252	74.1	31.0	<=40	.	Records/prescriptions	Follow-up	BB,RASi,MRA,DIG,DIUR
**76**	A Makubi, 2016	Non-acute	SWEDISH-HF	Sweden	2012-2013	1232	55.0	50.0	<50	.	Not reported	Baseline	BB,RASi,MRA,DIG,DIUR
**77**	Li, 2015	Non-acute	SWEDISH-HF	Sweden	2000-2013	18858	72.5	29.7	<40	.	Not reported	Baseline	BB,RASi,MRA,DIG,DIUR
**78**	Lund, 2018	Non-acute	SWEDISH-HF	Sweden	2000-2014	17193	71.9	27.6	<40	.	Not reported	Baseline	BB,RASI,MRA
**79**	Savarese, 2015	Non-acute	SWEDISH-HF	Sweden	2000-2012	22947	72.2	28.8	<40	.	Not reported	Baseline	BB,RASi,MRA,DIG,DIUR
**80**	Ozlek, 2019	Non-acute	APOLLON	Turkey	2018	246	68.0	42.3	40-49	45.0	Self reported	Baseline	BB,RASI,MRA,IVA,DIG,DIUR
**81**	Dokainish, 2016	Non-acute	INTER-CHF	Argentina, Chile, China, Colombia, Ecuador, Egypt, India, Malaysia, Mozambique, Nigeria, Philippines, Qatar, Saudiarabia, Southafrica, Sudan, Uganda	2012-2014	3306	57.7	31.7	<50	.	Not reported	Baseline	BB,RASi,MRA,DIG,DIUR
**82**	Komajda, 2016	Non-acute	QUALIFY	Armenia, Australia, Austria, Azerbaijan, Bahrain, Belarus, Brunei,	2013-2014	7092	63.1	26.0	<40	31.9	Not reported	Baseline	BB,RASI,MRA,IVA,DIG,DIUR

				Canada, China, Denmark, Ecuador, Egypt, Georgia, Germany, Greece, Hungary, Ireland, Jordan, Kazakhstan, Korea, Kuwait, Lebanon, Lithuania, Malaysia, Morocco, Poland, Portugal, Qatar, Romania, Russia, Slovakia, Spain, Thailand, Turkey, UK, UAE									
**83**	Komajda, 2017	Non-acute	QUALIFY	Armenia, Australia, Austria, Azerbaijan, Bahrain, Belarus, Brunei, Canada, China, Denmark,	2013-2014	6669	63.0	25.8	<=40	31.9	Not reported	Baseline	BB,RASi,MRA,DIG,DIUR

				Ecuador, Egypt, Georgia, Germany, Greece, Hungary, Ireland, Jordan, Kazakhstan, Korea, Kuwait, Lebanon, Lithuania, Malaysia, Morocco, Poland, Portugal, Qatar, Romania, Russia, Slovakia, Spain, Thailand, Turkey, UK, UAE									
**84**	Komajda, 2019	Non-acute	QUALIFY	Armenia, Australia, Austria, Azerbaijan, Bahrain, Belarus, Brunei, Canada, China, Denmark, Equador, Egypt, Georgia,	2013-2014	7315	63.2	26.2	<=40	33.0	Not reported	Baseline	BB,RASI,MRA,IVA

				Germany, Greece, Hungary, Ireland, Jordan, Kazakhstan, Korea, Kuwait, Lebanon, Lithuania, Malaysia, Morocco, Poland, Portugal, Qatar, Romania, Russia, Slovakia, Spain, Thailand, Turkey, UK, UAE									
**85**	Kubota, 2018	Non-acute	ASIAN-HF	China, Hongkong, India, Indonesia, Japan, Southkorea, Malaysia, Philippines, Singapore, Taiwan, Thailand	2010-2015	5232	59.7	21.9	<=40	27.3	Not reported	Baseline	BB,RASI,MRA,DIUR
**86**	Teng, 2018	Non-acute	ASIAN-HF	China, Hongkong, India, Indonesia, Japan,	2012-2015	5276	59.6	23.0	<=40	27.0	Not reported	Baseline	

				Southkorea, Malaysia, Philippines, Singapore, Taiwan, Thailand									
**87**	Arnold, 2019	Non-acute	ASIAN-HF	Indonesia, Philippines, India, China, Thailand, Malaysia	2010-2016	1160	59.8	21.7	<40	28.1	Records/prescriptions	Baseline	BB,RASI,SGLT,DIUR
**88**	Arnold, 2019	Non-acute	ASIAN-HF	Singapore, Hongkong, Taiwan, South Korea, Japan	2010-2016	1075	63.2	21.0	<40	27.0	Records/prescriptions	Baseline	BB,RASI,SGLT,DIUR
**89**	Yoo, 2015	Non-acute	COAST	South Korea, Taiwan, China	Not reporte	1470	66.3	33.5	<45	30.9	Not reported	Baseline	BB,RASi,MRA,DIG,DIUR
**90**	Pokharel, 2016	Non-acute	PINNACLE	India	2008-2014	15870	56.0	22.9	<40	.	Records/prescriptions	Baseline	BB,RASI
**91**	Harikrishnan, 2015	Non-acute	TRIVANDRUM HEART FAILURE REGISTRY	India	2013	894	61.2	31.0		.	Not reported	Baseline	BB,RASi,MRA,DIG,DIUR
**92**	Zhang, 2017	Acute	CHINA-HF	China	2012-2015	4482	60.0	30.2	<45	.	Not reported	Discharge	BB,RASI,MRA,DIUR
**93**	Goto, 2013	Acute	JCARE-CARD	Japan	Not reporte	527	63.5	26.9	<50	29.4	Not reported	Discharge	BB,RASi,MRA,DIG,DIUR
**94**	Tsuchihashi-Makaya, 2010	Acute	JCARE-CARD	Japan	2004-2005	947	66.3	27.8	<40	27.1	Not reported	Discharge	BB,RASi,MRA,DIG,DIUR
**95**	Akita, 2017	Acute	WET-HF	Japan	2005-2014	773	70.8	36.0	<45	32.2	Not reported	Discharge	BB,RASI,MRA,DIG,DIUR
**96**	Youn, 2012	Acute	KOREAN HEART FAILURE (KORHF) REGISTRY	South Korea	2004-2009	1527	69.1	44.1	<40	28.7	Not reported	Discharge	BB,RASI,MRA
**97**	Ahn, 2019	Non-acute	KOREAN-AHF	South Korea	2011-2014	2769	66.4	39.0	<40	26.8	Not reported	Baseline	BB,RASI,MRA
**98**	Gwag, 2018	Acute	KOREAN-AHF	South Korea	2011-2014	2577	67.8	39.4	<50	29.6	Not reported	Discharge	BB,RASI,MRA
**99**	Sung, 2018	Acute	HARVEST	Taiwan	2003-2012	423	84.5	21.7	<50	34.7	Records/prescriptions	Discharge	BB,RASi,MRA,DIG,DIUR

**100**	Chang, 2017	Non-acute	TSOC-HFREF	Taiwan	2013-2014	1509	63.9	27.6	<40	28.2	Not reported	Baseline	BB,RASI,MRA
**101**	Chang, 2019	Acute	TSOC-HFREF	Taiwan	2013-2014	393	69.2	28.0	<40	28.8	Not reported	Discharge	BB,RASi,MRA,DIG,DIUR

Table S6. Prevalence of GDMT in the acute and non-acute setting by WHO Regions

GDMT (by WHO Region)	Settings	
	**Acute** (inpatient discharge)	**Non-acute** (inpatient/ outpatient baseline)
Beta Blockers		
**Studies (N)|patients**	**50 | 131,846**	**237 | 811,301**
Americas (N=96)	75 (66–83)	84 (81–86)
Europe (N=142)	76 (69–82)	82 (80–84)
E. Mediterranean(N=7)	85 (48–97)	81 (29–98)
Western Pacific (N=29)	71 (64–77)	65 (57–72)
South-East Asia (N=8)	56*(28–80)	68 (45–85)
Africa (N=5)	37*(33–40)	63 (37–83)
**Total**<br>Heterogeneity: I^2= %	**73 (68–78)**<br>99.60	**81 (79–82)**<br>99.69
RAS inhibitors		
**Studies (patients)**	**49 | 130,956**	**234 |1,036,260**
Americas (N=99)	70 (62–77)	83 (80–86)
Europe (N=132)	81 (79–82)	85 (83–87)
E. Mediterranean (N=7)	81 (77–85)	76 (49–92)
Western Pacific (N=31)	76 (69–82)	73 (65–79)
South-East Asia (N=9)	81*(52–94)	67 (53–79)
Africa (N=5)	53*(30–75)	82 (66–91)
**Total**<br>Heterogeneity: I^2= %	**75 (72–78)**<br>99.21	**83 (81–85)**<br>99.81
MRA		
**Studies (patients)**	**39 | 61,475**	**191 | 763,771**
Americas (N=71)	24 (19–30)	27 (25–30)
Europe (N=117)	55 (42–67)	49 (46–52)
E. Mediterranean (N=5)	34*(29–39)	38 (24–54)
Western Pacific (N=25)	46 (34–59)	44 (35–55)
South-East Asia (N=7)	52*(26–77)	42 (26–61)
Africa (N=5)	39*(24–58)	53 (22–82)
**Total**<br>Heterogeneity: I^2= %	**40 (33–47)**<br>99.55	**42 (39–44)**<br>99.62
Diuretics[a]		
**Studies (patients)**	**33 | 69,733**	**183 | 781,852**
Americas (N=63)	71 (64–77)	74 (71–77)
Europe (N=110)	90 (71–97)	77 (75–79)
E. Mediterranean (N=8)	91 (81–96)	66 (47–81)
Western Pacific (N=25)	87 (80–91)	67 (57–75)
South-East Asia (N=5)	-	73 (52–87)
Africa (N=5)	68*(65–72)	87 (78–93)
**Total**<br>Heterogeneity: I^2= %	**82 (76–87)**<br>99.70	**75 (74–77)**<br>99.46
Data from studies involving multiple WHO regions not shown.		

Table S7. Prevalence of GDMT in acute and non-acute settings by income setting

GDMT (by income status)	Settings	
	**Acute** (inpatient discharge)	**Non-acute** (inpatient/ outpatient baseline)
Beta Blockers		
**Studies (N)| patients**	**50 | 131,846**	**237 | 811,301**
**High Income (N=238)**	76 (71–81)	82 (81–84)
**LMIC (N=44)**	61 (51–70)	69 (61–77)
U. Middle Income(N=36)	67 (59–75)	72 (66–78)
L. Middle Income (N=6)	37*(33–41)	46 (22–73)
Low Income (N=2)	37*(29–45)	61*(56–67)
**Total**<br>Heterogeneity: I^2= %	**73 (68–78)**<br>99.60	**81 (79–82)**<br>99.69
RAS inhibitors		
**Studies (patients)**	**49 | 130,956**	**234 |1,036,260**
**High Income (N=233)**	75 (72–79)	84 (82–86)
**LMIC (N=44)**	74 (64–81)	75 (68–81)
U. Middle Income (N=36)	79 (68–86)	77 (71–83)
L. Middle Income (N=6)	65*(61–68)	64 (44–81)
Low Income (N=2)	41*(33–49)	75*(70–79)
**Total**<br>Heterogeneity: I^2= %	**75 (72–78)**<br>99.21	**83 (81–85)**<br>99.81
MRA		
**Studies (patients)**	**39 | 61,475**	**191 | 763,771**
**High Income (N=195)**	34 (29–41)	40 (38–42)
**LMIC (N=29)**	56 (43–68)	48 (40–57)
U. Middle Income (N=23)	62 (51–71)	51 (42–61)
L. Middle Income (N=4)	31*(27–35)	39 (17–67)
Low Income (N=2)	49*(41–57)	22*(18–27)
**Total**<br>Heterogeneity: I^2= %	**40 (33–47)**<br>99.55	**42 (39–44)**<br>99.62
Diuretics [a]		
**Studies (patients)**	**33 | 69,733**	**183 | 781,852**
**High Income (N=181)**	80 (72–87)	74 (73–76)
**LMIC (N=30)**	81 (72–87)	79 (73–84)
U. Middle Income (N=23)	85 (73–92)	79 (72–84)
L. Middle Income (N=5)	68*(64–72)	81 (62–92)
Low Income (N=2)	71*(63–78)	79*(74–83)
**Total**<br>Heterogeneity: I^2= %	**82 (76–87)**<br>99.70	**75 (74–77)**<br>99.46
Data from studies involving multiple WHO regions not shown.		

Table S8: Prevalence of Beta blocker use by countries.

Country, by WHO Region [Number of studies (N)| Sample size (n)]	Overall Prevalence Percentage (95% CI)	Prevalence in the last ten years Percentage (95% CI)	Prevalence in the last five years Percentage (95% CI)	Settings	
				Acute	Non-acute
Americas					
Brazil (6|2028)	81 (71–88)	-	-	69 (66–72)	87 (73–95)
Canada (5|4071)	91 (88–93)	90 (86–93)	-	-	91 (88–93)
Guyana (1|232)	74 (68–79)	74 (68–79)	-	74 (68–79)	-
US (83|647,202)	82 (79–84)	84 (81–86)	-	76 (65–84)	83 (80–85)
Multiple (1|153)	75 (68–81)		-	75 (68–81)	-
**Total (96|653,686)**	**82 (80–84)**	**84 (81–86)**	**-**	**75 (66–83)**	**84 (81–86)**
Europe					
Austria (6|2302)	83 (77–88)	-	-	-	-
Belgium (10|2701)	91 (88–94)	96 (94–97)	96 (94–97)	83 (79–86)	83 (77–88)
Czech Republic (1|369)	93 (90–95)	-	-	-	93 (90–95)
Denmark (4|33,361)	74 (60–85)	-	-	-	74 (60–85)
France (5|4246)	75 (70–81)	71 (51–84)	-	79 (76–83)	74 (66–81)
Georgia (3|980)	22 (1–86)		-	-	22 (1–86)
Germany (9|8040)	87 (83–91)	90 (87–93)	-	-	87 (83–91)
Greece (1|332)	98 (96–99)	-	-	-	98 (96–99)
Israel (2|652)	95 (53–100)	84 (81–87)	84 (81–87)	-	95 (53–100)
Italy (24|222,389)	71 (65–77)	78 (73–82)	-	72 (65–79)	71 (63–78)
Netherlands (6|9491)	72 (57–83)	84 (83–84)	-	-	72 (57–83)
Norway (1|102)	93 (86–97)		-	-	93 (86–97)
Poland (10|37,548)	93 (89–96)	96 (95–97)	-	87 (87–88)	94 (87–97)
Portugal (6|1556)	74 (64–82)	75 (67–82)	-	75 (67–82)	74 (62–83)
Spain (19|11,553)	85 (79–89)	90 (72–97)	93 (91–95)	72 (68–75)	86 (80–91)
Sweden (8|50,932)	87 (82–90)	93 (81–98)	-	86 (85–90)	87 (80–92)
Turkey (8|3638)	79 (67–87)	84 (51–96)	70 (64–75)	71 (26–94)	81 (72–88)
UK (10|13,370)	69 (58–78)	91 (89–94)	-	59 (57–61)	70 (56–80)
Multiple (8|28,672)	85 (81–89)	91 (88–94)	-	81 (78–84)	86 (81–89)
**Total (141|232,234)**	**81 (79–83)**	**90 (87–92)**	**93 (88–96)**	**76 (69–82)**	**82 (80–84)**
Africas					
Ethiopia (2|455)	49 (27–72)	61 (56–67)	61 (56–67)	37 (29–45)	61 (56–67)
Ghana (1|610)	37 (33–41)	-	-	37 (33–41)	-
South Africa (1|200)	84 (78–89)	-	-	-	84 (78–89)
Tanzania (1|411)	38 (34–33)	38 (34–43)	-	-	38 (34–43)
**Total (5|1676)**	**53 (36–68)**	**50 (28–71)**	**61 (56–67)**	**37 (33–40)**	**63 (37–83)**
E. Mediterranean					
Iran (1|100)	19 (12–28)	-	-	-	19 (12–28)
Qatar (2|2176)	75 (18–98)	92 (89–95)	-	92 (89–95)	44 (42–47)
Saudi Arabia (2|2685)	95 (91 – 97)	-	-	93 (90–95)	96 (95–97)

UAE (1|102)	99 (93–100)	99 (93–100)	99 (93–100)	-	99 (93–100)
Multiple (1|334)	53 (48–58)	-	-	53 (48–58)	85 (81–89)
**Total (7|5397)**	**82 (54–95)**	**97 (78–99)**	**99 (93–100)**	**85 (48–97)**	**68 (22–94)**
South East Asia					
India (3|22,354)	59 (24–87)	70 (41–89)	-	-	59 (24–87)
Korea (4|5825)	57 (34–77)	27 (25–29)	-	56 (28–80)	59 (9–96)
Thailand (1|180)	95 (91–97)	95 (91–97)			95 (91–97)
**Total (8|28,359)**	**65 (47–80)**	**71 (36–91)**	**-**	**56 (28–80)**	**68 (45–85)**
Western Pacific					
Australia (5|2208)	44 (26–63)	65 (34–87)	-	61 (25–88)	33 (18–53)
China (6|7728)	64 (55–71)	67 (63–71)	69 (65–72)	70 (69–71)	62 (49–74)
Japan (7|4403)	77 (71–83)	83 (54–97)	-	79 (69–86)	76 (65–84)
New Zealand (1|451)	74 (70–78)	-	-	-	74 (70–78)
Singapore (N=1|244)	81 (75–85)	-	-	81 (75–85)	-
Taiwan (N=10|6761)	70 (62–77)	70 (59–79)	81 (78–83)	58 (50–65)	73 (64–80)
**Total (30|21,795)**	**67 (62–72)**	**70 (65–75)**	**75 (62–85)**	**71 (64–77)**	**65 (57–72)**

Table S9: Prevalence of RAS inhibitor (angiotensin converting enzyme receptor inhibitor and/or angiotensin receptor II blocker) use by countries.

**Country, by WHO Region** [Number of studies (N)| Sample size (n)]	**Overall Prevalence** Percentage (95% CI)	**Prevalence in the last ten years** Percentage (95% CI)	**Prevalence in the last five years** Percentage (95% CI)	**Settings**	
				Acute	Non-acute
Americas					
Brazil (6|1938)	87 (84–89)	93 (87–97)	-	84 (82–86)	88 (85–91)
Canada (5|4071)	89 (68–97)	71 (61–79)	-	-	89 (68–97)
Guyana (1|232)	60 (54–66)	60 (54–66)	-	60 (54–66)	-
US (86|874,237)	80 (77–83)	73 (64–80)	-	69 (61–77)	82 (79–85)
Multiple (1|153)	78 (71–84)	-	-	78 (71–84)	-
**Total (99|880,631)**	**81 (78–83)**	**74 (66–80)**	**-**	**70 (62–77)**	**83 (80–86)**
Europe					
Austria (5|2097)	87 (77–93)	-	-	-	87 (77–93)
Belgium (6|2104)	92 (85–96)	100 (96–100)	100 (96–100)	74 (70–77)	94 (88–97)
Czech Republic (1|369)	88 (84–91)	-	-	-	88 (84–91)
Denmark (4|33,361)	82 (68–91)	-	-	-	82 (69–79)
France (5|4246)	77 (63–86)	66 (32–89)	-	83 (77–87)	71 (40–90)
Georgia (3|980)	49 (9–91)	-	-	-	49 (9–90)
Germany (8|7659)	88 (81–93)	91 (88–94)	-	-	88 (82–93)
Israel (2|652)	96 (43–100)	84 (81–87)	84 (81–87)	-	96 (43–100)
Italy (23|22,158)	84 (79–88)	84 (59–95)	-	78 (71–84)	84 (79–89)
Netherlands (6|9491)	74 (53–87)	82 (81–83)	-	-	74 (53–87)
Norway (1|102)	97 (91–99)	-	-	-	97 (91–99)
Poland (9|31,985)	83 (73–90)	91 (89–92)	-	82 (81–82)	84 (69–92)
Portugal (6|1556)	88 (78–94)	59 (49–67)	-	59 (49–67)	91 (85–95)
Spain (19|12,044)	89 (85–92)	92 (76–98)	96 (94–97)	84 (66–93)	90 (85–93)
Sweden (8|50,932)	90 (83–94)	96 (95–98)	-	82 (81–82)	91 (84–95)
Turkey (8|3638)	85 (75–90)	80 (41–96)	62 (56–68)	88 (47–98)	83 (73–89)
UK (10|13,370)	84 (79–88)	91 (88–93)	-	-	84 (79–88)
Multiple (8|34,010)	79 (60–90)	91 (89–93)	-	-	79 (60–90)
**Total (132|230,754)**	**85 (83–87)**	**88 (85–91)**	**93 (83–97)**	**81 (79–82)**	**85 (83–87)**
Africas					
Ethiopia (2|455)	59 (26–86)	75 (70–79)	75 (70–79)	41 (33–49)	75 (70–79)
Ghana (1|610)	65 (61–68)	-	-	65 (61–68)	-
South Africa (1|200)	74 (67–80)	-	-	-	74 (68–80)
Tanzania (1|411)	92 (89–94)	92 (89–94)	-	-	92 (89–94)
**Total (5|1676)**	**72 (56–84)**	**86 (60–96)**	**75 (70–79)**	**53 (30–75)**	**82 (66–91)**

E. Mediterranean					
Iran (1|100)	64 (54–73)	-	-	-	64 (54–73)
Qatar (2|2176)	62 (23–89)	79 (75–83)	-	79 (75–83)	41 (38–43)
Saudi Arabia (1|387)	85 (81–88)	-	-	85 (81–88)	
UAE (1|102)	97 (91–99)	97 (91–99)	97 (91–99)	-	97 (91–99)
Multiple (2|1612)	80 (78–82)	80 (78–82)	-	79 (74–83)	80 (78–82)
**Total (7|4377)**	**78 (61–89)**	**83 (76–89)**	**97 (91–99)**	**81 (77–85)**	**61 (33–83)**
South East Asia				-	
India (3|22,354)	49 (26–73)	58 (39–75)	-	-	49 (26–73)
Korea (5|5982)	79 (57–92)	40 (38–42)	-	81 (52–95)	78 (32–96)
Thailand (1|180)	82 (75–87)	82 (75–87)	-	-	82 (75–87)
**Total (9|28,516)**	**71 (57–82)**	**60 (43–75)**	**-**	**81 (52–94)**	**67 (53–79)**
Western Pacific					
Australia (6|2333)	53 (40–65)	58 (46–69)	-	63 (51– 74)	40 (14 –73)
China (6|7728)	70 (55–82)	79 (53–93)	96 (94–97)	67 (66–69)	71 (45–88)
Japan (7|3745)	87 (80–92)	81 (75–85)	-	84 (72–92)	91 (76–97)
New Zealand (1|451)	68 (64–72)	-	-	-	68 (64–72)
Singapore (1|244)	86 (81–90)	-	-	86 (81–90)	
Taiwan (10|6761)	75 (68–81)	76 (64–85)	72 (69–75)	76 (45–92)	75 (67–82)
**Total (31|21,262)**	**74 (69–79)**	**75 (69–81)**	**88 (47–98)**	**76 (69–82)**	**73 (65–79)**

Table S10. Prevalence of mineralocorticoid receptor blockers (MRA) use by countries.

**Country, by WHO Region** [Number of studies (N)| Sample size (n)]	**Overall Prevalence** Percentage (95% CI)	**Prevalence in the last ten years** Percentage (95% CI)	**Prevalence in the last five years** Percentage (95% CI)	**Settings**	
				Acute	Non-acute
Americas					
Brazil (3|548)	48 (13–84)	69 (60–77)	-	-	48 (13–84)
Canada (5|4071)	42 (39–46)	41 (35–47)	-	-	43 (39–46)
Guyana (1|232)	42 (36–49)	42 (36–49)	-	42 (36–49)	-
US (61|626,222)	24 (22–26)	31 (26–36)	-	21 (16–27)	25 (22–27)
Multiple (1|153)	46 (38–54)	-	-	46 (38–54)	-
**Total (71|631,226)**	**27 (24–29)**	**35 (30–40)**	**-**	**24 (19–30)**	**27 (25–30)**
Europe					
Austria (5|1951)	44 (35–53)	-	-	-	44 (35–53)
Belgium (9|2457)	72 (60–82)	84 (81–86)	84 (81–86)	38 (8–58)	76 (66–84)
Czech Republic (1|369)	78 (74–82)	-	-	-	78 (74–82)
Denmark (4|27,267)	47 (32–62)	-	-	-	47 (32–62)
France (4|3454)	28 (19–39)	15 (3–53)	-	31 (28–35)	26 (12–47)
Germany (9|8040)	49 (40–57)	49 (44–54)	-	-	48 (40–57)
Israel (1|552)	44 (40–48)	44 (40–48)	44 (40–48)	-	44 (40–48)
Italy (20|19,524)	47 (42–52)	38 (25–53)	-	74 (57–86)	42 (37–47)
Netherlands (2|8460)	45 (30–61)	53 (52–54)	-	-	45 (30–61)
Norway (1|102)	22 (15–31)		-	-	22 (15–31)
Poland (8|9348)	61 (54–68)	73 (68–77)	-	-	61 (54–68)
Portugal (5|1290)	47 (34–60)	31 (24–40)	-	31 (24–40)	51 (36–65)
Spain (18|11.629)	55 (46–63)	65 (46–80)	72 (63–79)	27 (16–43)	59 (50–67)
Sweden (6|18,781)	34 (24–45)	56 (52–60)	-	-	34 (24–45)
Turkey (6|2908)	58 (42–72)	54 (8–94)	24 (19–30)	68 (53–80)	53 (32–72)
UK (9|11.586)	34 (28–40)	59 (54–63)	-	-	34 (28–40)
Multiple (9|34,711)	55 (44–67)	74 (67–80)	-	80 (77–82)	52 (40–64)
**Total (117|162,429)**	**50 (47–53)**	**61 (56–66)**	**72 (59–82)**	**55 (42–67)**	**49 (46–52)**
Africas					
Ethiopia (2|455)	34 (14–63)	22 (18–27)	22 (18–27)	49 (41–57)	22 (18–27)
Ghana (1|610)	31 (27–35)	-	-	31 (27–35)	-
South Africa (1|200)	64 (57–70)	-	-	-	64 (57–70)
Tanzania (1|411)	74 (70–78)	74 (70–78)	-	-	74 (70–78)
**Total (5|1676)**	**48 (28–68)**	**47 (9–90)**	**22 (18–27)**	**39 (24–58)**	**53 (22–82)**
E. Mediterranean					
Iran (1|100)	8 (4–15)	-	-	-	8 (4–15)

Qatar (1|400)	34 (29–39)	34 (29–38)	-	34 (29–38)	-
Saudi Arabia (1|2298)	57 (55–59)	-	-	-	57 (55–59)
UAE (1|102)	60 (50–69)	60 (50–69)	60 (50–69)	-	60 (60–69)
Multiple (1|1278)	36 (34–39)	36 (34–39)	-	-	36 (34–39)
**Total (5|4178)**	**37 (25–51)**	**42 (32–52)**	**60 (50–69)**	**34 (29–39)**	**38 (24–54)**
South East Asia					-
India (1|894)	47 (44–50)	47 (44–50)	-	-	47 (44–50)
Korea (5|5982)	41 (23–62)	20 (18–21)	-	52 (26–77)	35 (18–57)
Thailand (1|180)	61 (53–67)	61 (53–67)	-	-	61 (53–67)
**Total (7|7056)**	**45 (30–61)**	**41 (19–67)**	**-**	**52 (26–77)**	**42 (26–61)**
Western Pacific					
Australia (4|1740)	31 (15–52)	26 (8–58)	-	26 (8–58)	36 (7–80)
China (5|6609)	44 (19–72)	48 (18–80)	29 (26–32)	74 (73–75)	36 (19–56)
Japan (8|4630)	46 (41–51)	53 (50–56)	-	47 (44–50)	44 (34–55)
New Zealand (1|451)	25 (21–29)	-	-	-	25 (21–29)
Singapore (1|244)	35 (29–41)	-	-	35 (29–41)	-
Taiwan (6|5007)	61 (53–69)	55 (42–67)	67 (64–70)	57 (39–73)	63 (53–72)
**Total (25|18,681**)	**45 (35–53)**	**46 (34–59)**	**48 (16–82)**	**46 (34–59)**	**44 (35–55)**

Table S11. Prevalence of Diuretic use by countries.

Country, by WHO Region [Number of studies (N)| Sample size (n)]	Overall Prevalence Percentage (95% CI)	Prevalence in the last ten years Percentage (95% CI)	Prevalence in the last five years Percentage (95% CI)	Settings	
				Acute	Non-acute
Americas					
Brazil (3|598)	77 (60–88)	-	-	-	77 (60–77)
Canada (4|3715)	85 (69–93)	84 (83–85)	-	-	85 (69–93)
US (56|651,300)	73 (70–75)	82 (82–83)	-	71 (64–77)	73 (70–76)
**Total (63|655,613)**	**73 (71–76)**	**62 (54–69)**	**-**	**71 (64–77)**	**74 (71–77)**
Europe					
Austria (4|1823)	76 (63–86)	90 (87–92)	-	90 (87–92)	69 (60–77)
Belgium (9|2158)	68 (56–77)	53 (49–58)	5 (49–58)	84 (80–88)	64 (54–73)
Czech Republic (1|369)	77 (71–82)	77 (71–82)	-	-	77 (71–82)
Denmark (2|6811)	75 (72–78)	-	-	-	75 (72–78)
France (5|4246)	80 (69–88)	85 (72–92)	79(74–83)	85 (53–96)	76 (69–82)
Georgia (1|400)	90 (88–92)	-	-	-	90 (88–92)
Germany (9|8040)	83 (76–89)	-	-	-	83 (75–89)
Israel (1|100)	68 (64–72)	-	-	68 (64–72)	
Italy (23|22,885)	82 (78–86)	85 (75–92)	-	96 (95–97)	81 (77–84)
Netherlands (5|9352)	67 (61–72)	-	-	-	67 (61–72)
Norway (1|102)	81 (79–83)	81 (79–83)	-	-	81 (79–83)
Poland (7|36,088)	76 (53–90)	85 (82–87)	-	45 (45–46)	80 (69–88)
Portugal (3|612)	89 (84–92)	-	-	-	89 (84–92)
Spain (11|7530)	79 (66–87)	83 (81–85)	-	21 (18–24)	83 (79–86)
Sweden (7|21,711)	57 (43–69)	20 (1–88)	-	-	57 (43–69)
Turkey (4|1890)	79 (70–87)	74 (69–79)	71 (66–77)	88 (86–90)	75 (72–78)
UK (9|10,112)	67 (56–77)	75 (71–79)	-	-	67 (56–77)
Multiple (8|29,268)	77 (64–86)	83 (83–84)	-	68 (64–71)	78 (62–89)
**Total (110|163,497)**	**78 (75–81)**	**74 (68–79)**	**57 (49–65)**	**90 (71–97)**	**77 (75–79)**
Africas					
Ethiopia (2|455)	82 (47–96)	-	-	-	83 (47–96)
Ghana (1|610)	73 (67–78)	-	-	-	73 (67–78)
South Africa (1|200)	93 (89–96)	-	-	-	93 (89–96)
Tanzania (1|411)	88 (84–91)	88 (84–91)	-	-	88 (84–91)
**Total (5|1676)**	**81 (71–89)**	**84 (73–91)**	**79 (74–83)**	**68 (65–72)**	**87 (78–93)**
E. Mediterranean					
Iran (1|100)	85 (82–87)	-	-	-	85 (82–87)
Qatar (2|2176)	87 (12–100)	98 (96–99)	-	98 (96–99)	48 (46–50)

Saudi Arabia (2|2685)	82 (74–87)	-	-	85 (81–88)	78 (76–80)
UAE (1|102)	76 (66–83)	75 (66–83)	76 (66–83)	-	75 (66–83)
Multiple (2|1612)	70 (43–88)	-	-	-	70 (43–88)
**Total (8|6675)**	**78 (65–87)**	**89 (72–96)**	**76 (66–83)**	**91 (81–96)**	**66 (47–81)**
South East Asia					
India (2|6484)	81 (70–89)	85 (81–89)	-	-	81 (70–89)
Korea (2|389)	89 (87–91)	90 (87–93)	-	89 (87–91)	
Thailand (1|180)	69 (62–76)	69 (62–76)	-	-	69 (62–76)
**Total (5|7053)**	**73 (52–87)**	**85 (67–94)**	**-**	**-**	**73 (52–87)**
Western Pacific					
Australia (4|1353)	73 (21–96)	96 (93–97)	-	95 (92–96)	59 (10–95)
China (5|7051)	64 (50–77)	74 (69–79)	-	72 (71–74)	62 (38–81)
Japan (7|4103)	90 (82–95)	91 (86–95)	-	99 (97–100)	82 (70–89)
Singapore (1|244)	88 (84–92)	-	-	89 (84–92)	-
Taiwan (8|4320)	75 (67–82)	68 (49–83)	-	88 (85–90)	69 (62–76)
**Total (25|17,071)**	**75 (69–81)**	**83 (74–89)**	**-**	**87 (80–91)**	**67 (57–75)**

Table S12. Prevalence of other Guideline-directed Medical Therapy (GDMT) by WHO regions.

GDMT (by WHO Region)	Overall Prevalence Percentage (95% CI)	Prevalence in the last ten years Percentage (95% CI)	Prevalence in the last five years Percentage (95% CI)	Settings	
				**Acute**	**Non-acute**
Digoxin					
Studies (Patients)	**152|648,023**	**37|157,261**	**3|705**	**26|30,594**	**126|617,429**
Americas (N=55)	29 (27–32)	17 (14–19)	-	40 (32–48)	27 (25–30)
Europe (N=66)	21 (19–24)	16 (13–20)	8 (6–11)	24 (15–36)	21 (19–24)
E. Mediterranean (N=5)	30 (15–49)	20 (16–24)	-	30 (9–66)	29 (12–53)
Western Pacific (N=16)	29 (21–37)	27 (19–35)	-	28 (21–36)	29 (17–46)
South-East Asia (N=5)	26 (8–59)	12 (3–41)	-	-	26 (8–59)
Africa (N=5)	23 (13–37)	30 (11–60)	14 (11–17)	16 (13–18)	28 (15–47)
**Total**	**25 (24–27)**	**18 (16–20)**	**11 (6–21)**	**31 (26–36)**	**24 (23–26)**
Heterogeneity: I^2= %	99.3	98.5	88.5	99.0	99.2
Ivabradine					
Studies (Patients)	**26|37,318**	**17|30,444**	**3|443**	**-**	**26|37,318**
Americas (N=1)	1 (1–2)	1 (1–2)	-	-	1 (1–2)
Europe (N-21)	7 (5–10)	8 (5–13)	7 (3–18)	-	7 (5–10)
E. Mediterranean (N=1)	4 (3–6)	4 (3–6)	-	-	4 (3–6)
Western Pacific (N=1)	10 (8–12)	10 (8–12)	10 (8–12)	-	10 (8–12)
South-East Asia (N=2)	9 (1–40)	9 (1–40)	-	-	9 (1–40)
Africa	-	-	-	-	-
**Total**	**7 (5–9)**	**7 (5–11)**	**8 (5–13)**	-	**7 (5–9)**
Heterogeneity: I^2= %	98.6	99.0	76.6		98.6
ARNI					
Studies (Patients)	**5|63,623**	**4|60,499**	**1|932**	**1|16,674**	**4|46,949**
Americas (N=4)	5 (3–10)	7 (4–14)	-	-	4 (1–11)
Europe	-	-	-	-	-
E. Mediterranean	-	-	-	-	-
Western Pacific (N=1)	50 (47–53)	50 (47–53)	50 (47–53)	50 (47–53)	50 (47–53)
South-East Asia	-	-	-	-	-
Africa	-	-	-	-	-
**Total**	**8 (3–19)**	**13 (5–29)**	**50 (47–53)**	**50 (47–53)**	**7 (2–24)**
Heterogeneity: I^2= %	99.8	99.9	-	-	99.9
Hydralazine/Nitrate					
Studies (Patients)	**14|72,683**	**6|43,505**	**-**	**3|3783**	**11|68,900**
Americas (N=12)	13 (11–16)	16 (8–29)	-	13 (4– 38)	13 (11–16)
Europe (N=1)	37 (28–46)	-	-	-	37 (28–46)
E. Mediterranean	-	-	-	-	-

Western Pacific	-	-	-	-	-
South-East Asia (N=1)	13 (9–19)	13 (9–19)	-	-	13 (9–19)
Africa	-	-	-	-	-
**Total**	**14 (12–17)**	**15 (8–26)**	-	**13 (4–38)**	**15 (12–18)**
Heterogeneity: I^2= %	98.3	98.8		99.2	98.1
SGLT-2 Inhibitors					
Studies (Patients)	1 | 2235	-	-	-	1 | 2235
Western Pacific/ South-East Asia	0.2 (0–1)	-	-	-	0.2 (0–1)
**Total**	**0.2 (0–1)**	**-**	**-**	**-**	**0.2 (0–1)**
Heterogeneity: I^2= %					

Table S13: Prevalence of other GDMT by income status

GDMT (by income status)	Overall *Prevalence* *Percentage (95% CI)*	Prevalence in the last ten years Percentage (95% CI)	Prevalence in the last five years Percentage (95% CI)	Settings	
				**Acute**	**Non-acute**
Digoxin					
Studies (Patients)	**152|648,023**	**37|157,261**	**3|705**	**26|30,594**	**126|617,429**
**High Income (N=124)**	25 (23–26)	17 (15–18)	9 (6–15)	30 (25–36)	24 (22–25)
U. Middle Income (N=19)	29 (22–38)	22 (11–40)	7 (5–11)	34 (25–45)	28 (19–40)
L. Middle Income (N=5)	23 (9–49)	22 (5–60)	-	15 (13–18)	26 (8–60)
Low Income (N=2)	18 (15–22)	19 (15–24)	19 (15–24)	18 (12–25)	19 (15–24)
**LMIC (N=26)**	27 (20–36)	22 (11–38)	12 (5–28)	28 (19–38)	27 (18–39)
Heterogeneity: I^2= %	99.3	98.5	88.5	99.0	99.2
Ivabradine					
Studies (Patients)	**26|37,318**	**17|30,444**	**3|443**	**-**	**26|37,318**
**High Income (N=19)**	6 (4–9)	6 (3–10)	11 (7–17)	-	6 (4–9)
U. Middle Income (N=3)	5 (2 – 12)	6 (3–12)	7 (3 – 15)	-	5 (2 – 12)
L. Middle Income (N=1)	2 (2–21)	20 (19–21)	-	-	2 (2–21)
Low Income	-	-	-	-	-
**LMIC (N=4)**	8 (4–15)	8 (4–15)	7 (3–15)	-	8 (4–15)
Heterogeneity: I^2= %	98.6	99.0	76.6		98.6
ARNI					
Studies (Patients)	**5|63,623**	**4|60,499**	**1|932**	**1|16,674**	**4|46,949**
**High Income (N=3)**	5 (3–10)	7 (4–14)	-	61 (57–65)	4 (1–11)
U. Middle Income (N=1)	50 (47–53)	50 (47–53)	50 (47–53)	-	50 (47–53)
L. Middle Income	-	-	-	-	-
Low Income	-	-	-	-	-
**LMIC (N=1)**	50 (47–53)	50 (47–53)	50 (47–53)	-	50 (47–53)
Heterogeneity: I^2= %	99.8	99.9	0.0	0.0	99.9
Hydralazine/Nitrate					
Studies (Patients)	**14|72,683**	**6|43,505**	**-**	**3|3783**	**11|68,900**
**High Income (N=13)**	14 (12–17)	16 (8–29)	-	13 (4–38)	15 (12–18)
U. Middle Income (N=1)	13 (9–19)	13 (10–19)	-	-	13 (9–19)
Lower Middle Income	-	-	-	-	-
Low Income	-	-	-	-	-
**LMIC (N=1)**	13 (9–19)	13 (10–19)	-	-	13 (9–19)
Heterogeneity: I^2= %	98.3	98.8		99.2	98.1

Table S14. Quality assessment using Joanna Briggs Institute (JBI) critical appraisal tool

**Author et al. (Year)**	**Were the criteria for inclusion in the sample clearly defined?**	**Were the study subjects and the setting described in detail?**	**Was the exposure measured in a valid and reliable way?**	**Were objective, standard criteria used for measurement of the condition?**	**Were confounding factors identified? (Age, comorbid conditions)**	**Were strategies to deal with confounding factors stated?**	**Were the outcomes measured in a valid and reliable way?**	**Was appropriate statistical analysis used?**	**Interpretation**
Albert, 2010	✓	✓	N/A	✓	✓	✗	✓	✓	Low risk
Albuquerque, 2014	✓	✓	N/A	✓	✓	✗	✓	✓	Low risk
Allen, 2014	✓	✓	N/A	✓	✓	✗	✓	✓	Low risk
Altunbas, 2016	✓	✓	N/A	✓	✓	✗	✓	✓	Low risk
Alvarez, 2019	✓	✓	N/A	✓	✓	✗	✓	✓	Low risk
Angermann, 2011	✓	✓	N/A	✓	✓	✓	Unclear	✓	Low risk
Anne Naka✗1	✓	✓	N/A	✓	✓	✗	✓	✓	Low risk
Arnold, 2019	✓	✓	N/A	✓	✓	✗	✓	✓	Low risk
Arnold, 2019	✓	✓	N/A	✓	✓	✗	✓	✓	Low risk
Asghar, 2010	✓	✓	N/A	✓	✓	✗	✓	✓	Low risk
Atallah, 2019	✓	✓	N/A	✓	✓	✗	✓	✓	Low risk
Atherton, 2012	✓	✓	N/A	✓	✓	✗	✓	✓	Low risk
Ayan, 2019	✓	✓	N/A	✓	✓	✗	✓	✓	Low risk
Barywani, 2015	✓	✓	N/A	✓	✓	✗	✓	✓	Low risk
Bayoumi, 2019	✓	✓	N/A	✓	✓	✗	✓	✓	Low risk
Bertero, 2019	✓	✓	N/A	✓	✓	✓	Unclear	✓	Low risk
Bhattacharya, 2010	✓	✓	N/A	✓	✓	✗	✓	✓	Low risk
Bitar, 2019	✓	✓	N/A	✓	✓	✓	✓	✓	Low risk
Blecker, 2014	✓	✓	N/A	✓	✓	✗	✓	✓	Low risk

Bonsu, 2017	✓	✓	N/A	✓	✓	✗	✓	✓	Low risk
Busson, 2018	✓	✓	N/A	✓	✓	✗	✓	✓	Low risk
Butler, 2019	✓	✓	N/A	✓	✓	✗	✓	✓	Low risk
CH Wang, 2018	✓	✓	N/A	✓	✓	✗	✓	✓	Low risk
Chang, 2013	✓	✓	N/A	✓	✓	✗	✓	✓	Low risk
Chang, 2017	✓	✓	N/A	✓	✓	✓	Unclear	✓	Low risk
Chyu, 2014	✓	✓	N/A	✓	✓	✗	✓	✓	Low risk
Cleland, 2011	✓	✓	N/A	✓	✓	✓	Unclear	✓	Low risk
Cohen Solal, 2012	✓	✓	N/A	✓	✓	✗	✓	✓	Low risk
Corra, 2013	✓	✓	N/A	✓	✓	✗	✓	✓	Low risk
Correa, 2016	✓	✓	N/A	✓	✓	✓	✓	✓	Low risk
Crissinger, 2015	✓	✓	N/A	✓	✓	✗	✓	✓	Low risk
Cutshall, 2018	✓	✓	N/A	✓	✓	✗	✓	✓	Low risk
DeVore, 2018	✓	✓	N/A	✓	✓	✓	Unclear	✓	Low risk
Dunlay, 2011	✓	✓	N/A	✓	✓	✗	✓	✓	Low risk
Durstenfeld, 2019	✓	✓	N/A	✓	✓	✗	✓	✓	Low risk
Ege, 2012	✓	✓	N/A	✓	✓	✗	✓	✓	Low risk
Ekmekci, 2016	✓	✓	N/A	✓	✓	✗	✓	✓	Low risk
El-Chami, 2010	✓	✓	N/A	✓	✓	✗	✓	✓	Low risk
Elmariah, 2010	✓	✓	N/A	✓	✓	✗	✓	✓	Low risk
El-Menyar, 2015	✓	✓	N/A	✓	✓	✗	✓	✓	Low risk
El-Refai, 2013	✓	✓	N/A	✓	✓	✗	✓	✓	Low risk
Fonarow, 2011	✓	✓	N/A	✓	✓	✗	✓	✓	Low risk
Fragasso, 2013	✓	✓	N/A	✓	✓	✗	✓	✓	Low risk
Fragasso, 2013	✓	✓	N/A	✓	✓	✗	✓	✓	Low risk
Frankenstein, 2010	✓	✓	N/A	✓	✓	✗	✓	✓	Low risk

Gagne, 2018	✓	✓	N/A	✓	✓	✗	✓	✓	Low risk
Gavazzi, 2015	✓	✓	N/A	✓	✓	✗	✓	✓	Low risk
Gilstrap, 2018	✓	✓	N/A	✓	✓	✗	✓	✓	Low risk
Gjesing, 2013	✓	✓	N/A	✓	✓	✗	✓	✓	Low risk
Grainger, 2013	✓	✓	N/A	✓	✓	✗	✓	✓	Low risk
Gurwitz, 2017	✓	✓	N/A	✓	✓	✓	✓	✓	Low risk
Guzman, 2018	✓	✓	N/A	✓	✓	✓	Unclear	✓	Low risk
H Altay, 2012	✓	✓	N/A	✓	✓	✗	✓	✓	Low risk
Hebert, 2010	✓	✓	N/A	✓	✓	✗	✓	✓	Low risk
Hebert, 2011	✓	✓	N/A	✓	✓	✗	✓	✓	Low risk
Hoong, 2015	✓	✓	N/A	✓	✓	✗	✓	✓	Low risk
Jan StÃ¥lhammar	✓	✓	N/A	✓	✓	✗	✓	✓	Low risk
Jehu S. Mathew	✓	✓	N/A	✓	✓	✗	✓	✓	Low risk
Jonsson, 2018	✓	✓	N/A	✓	✓	✗	✓	✓	Low risk
Jorge, 2013	✓	✓	N/A	Unclear	✓	✓	✓	✓	Low risk
Juilliere, 2014	✓	✓	N/A	✓	✓	✓	✓	✓	Low risk
Kang, 2015	✓	✓	N/A	✓	✓	✗	✓	✓	Low risk
Kato, 2013	✓	✓	N/A	✓	✓	✗	✓	✓	Low risk
Kelesidis, 2013	✓	✓	N/A	✓	✓	✗	✓	✓	Low risk
Kessing, 2019	✓	✓	N/A	✓	✓	✗	✓	✓	Low risk
Kim, 2019	✓	✓	N/A	✓	✓	✗	✓	✓	Low risk
Klassen, 2018	✓	✓	N/A	✓	✓	✗	✓	✓	Low risk
Kontogeorgos, 2017	✓	✓	N/A	✓	✓	✗	✓	✓	Low risk
Lam, 2018	✓	✓	N/A	✓	✓	✗	✓	✓	Low risk
Laszczynska, 2017	✓	✓	N/A	✓	✓	✗	✓	✓	Low risk
Lau, 2019	✓	✓	N/A	✓	✓	✗	✓	✓	Low risk

Lee, 2015	✓	✓	N/A	✓	✓	✓	Unclear	✓	Low risk
Li, 2018	✓	✓	N/A	✓	✓	✗	✓	✓	Low risk
Lin, 2010	✓	✓	N/A	✓	✓	✗	✓	✓	Low risk
Loh, 2013	✓	✓	N/A	✓	✓	✗	✓	✓	Low risk
Luo, 2019	✓	✓	N/A	✓	✓	✗	✓	✓	Low risk
Luzum, 2019	✓	✓	N/A	✓	✓	✗	✓	✓	Low risk
M Fudim, 2018	✓	✓	N/A	✓	✓	✗	✓	✓	Low risk
Marques, 2017	✓	✓	N/A	✓	✓	✗	✓	✓	Low risk
Martens, 2017	✓	✓	N/A	✓	✓	✗	✓	✓	Low risk
Martens, 2018	✓	✓	N/A	✓	✓	✗	✓	✓	Low risk
Martens, 2019	✓	✓	N/A	✓	✓	✗	✓	✓	Low risk
Martens, 2019	✓	✓	N/A	✓	✓	✗	✓	✓	Low risk
Martinez-Milla, 2019	✓	✓	N/A	✓	✓	✗	✓	✓	Low risk
May Al-khateeb	✓	✓	N/A	✓	✓	✗	✓	✓	Low risk
McNamara, 2011	✓	✓	N/A	✓	✓	✓	Unclear	✓	Low risk
Mohanty, 2019	✓	✓	N/A	✓	✓	✓	✓	✓	Low risk
Murninkas, 2019	✓	✓	N/A	✓	✓	✗	✓	✓	Low risk
Murphy, 2019	✓	✓	N/A	✓	✓	✗	✓	✓	Low risk
Nasser, 2017	✓	✓	N/A	✓	✓	✗	✓	✓	Low risk
Niriayo, 2019	✓	✓	N/A	✓	✓	✓	✓	✓	Low risk
Opolski, 2017	✓	✓	N/A	✓	✓	✗	✓	✓	Low risk
Ozlek, 2019	✓	✓	N/A	✓	✓	✗	✓	✓	Low risk
Pandey, 2016	✓	✓	N/A	✓	✓	✗	✓	✓	Low risk
Passos, 2016	✓	✓	N/A	✓	✓	✗	✓	✓	Low risk
Pokharel, 2016	✓	✓	N/A	✓	✓	✗	✓	✓	Low risk
Rahhal, 2017	✓	✓	N/A	✓	✓	✗	✓	✓	Low risk

Richardson, 2016	√	√	N/A	√	√	✗	√	√	Low risk
Rickard, 2014	√	√	N/A	√	√	✗	√	√	Low risk
Roth, 2016	√	√	N/A	√	√	✗	√	√	Low risk
Roth, 2017	√	√	N/A	√	√	✗	√	√	Low risk
Ruf, 2010	√	√	N/A	√	√	✗	√	√	Low risk
Rywik, 2011	√	√	N/A	√	√	✗	√	√	Low risk
Sargento, 2016	√	√	N/A	√	√	✗	√	√	Low risk
Sargento, 2017	√	√	N/A	√	√	✗	√	√	Low risk
Sartipy, 2014	√	√	N/A	√	√	✗	√	√	Low risk
Scrutinio, 2012	√	√	N/A	√	√	✗	√	√	Low risk
Scrutinio, 2015	√	√	N/A	√	√	✗	√	√	Low risk
SHI Chuan	√	√	N/A	√	√	√	√	√	Low risk
Shore, 2012	√	√	N/A	√	√	✗	√	√	Low risk
Silavanich, 2019	√	√	N/A	√	√	✗	√	√	Low risk
Son, 2011	√	√	N/A	√	√	✗	√	√	Low risk
Steinman, 2011	√	√	N/A	√	√	Unclear	√	√	Low risk
Sung, 2018	√	√	N/A	√	√	✗	√	√	Low risk
Teng, 2018	√	√	N/A	√	√	√	Unclear	√	Low risk
V Khalil, 2017	√	√	N/A	√	√	✗	√	√	Low risk
Veenis, 2019	√	√	N/A	√	√	√	Unclear	√	Low risk
Veien, 2011	√	√	N/A	√	√	✗	√	√	Low risk
WA Teeter, 2012	√	√	N/A	√	√	✗	√	√	Low risk
Wilcox, 2012	√	√	N/A	√	√	✗	√	√	Low risk
Witte, 2018	√	√	N/A	√	√	√	√	√	Low risk
Wong, 2010	√	√	N/A	√	√	✗	√	√	Low risk
Woodruff, 2016	√	√	N/A	√	√	✗	√	√	Low risk

Zepeda, 2019	✓	✓	N/A	✓	✓	✗	✓	✓	Low risk
A Bayés-Genís, 2015	✓	✓	N/A	✓	✓	✗	Unclear	✓	Intermediate
A Makubi, 2016	✓	✓	N/A	✓	✓	✗	Unclear	✓	Intermediate
A Makubi, 2016	✓	✓	N/A	✓	✓	✗	Unclear	✓	Intermediate
Abebe, 2016	✓	✓	N/A	✓	✓	✗	Unclear	✓	Intermediate
Abi Khalil, 2017	✓	✓	N/A	✓	✓	✗	Unclear	✓	Intermediate
Abi Khalil, 2018	✓	✓	N/A	✓	✓	✗	Unclear	✓	Intermediate
Agostoni, 2010	✓	✓	N/A	✓	✓	✗	Unclear	✓	Intermediate
Agra Bermejo, 2018	✓	✓	N/A	✓	✓	✗	Unclear	✓	Intermediate
Ahn, 2019	✓	✓	N/A	✓	✓	✗	Unclear	✓	Intermediate
Ajam, 2018	✓	✓	N/A	✓	✓	✗	Unclear	✓	Intermediate
Akita, 2017	✓	✓	N/A	✓	✓	✗	Unclear	✓	Intermediate
Alba, 2013	✓	✓	N/A	✓	✓	✗	Unclear	✓	Intermediate
Aleksova, 2011	✓	✓	N/A	✓	✓	✗	Unclear	✓	Intermediate
AlJaroudi, 2015	✓	✓	N/A	✓	✓	✗	Unclear	✓	Intermediate
Alkhawam, 2019	✓	✓	N/A	✓	✓	✗	Unclear	✓	Intermediate
Allen, 2018	✓	✓	N/A	Unclear	✓	✗	✓	✓	Intermediate
AlShamiri, 2018	✓	✓	N/A	✓	✓	✗	Unclear	✓	Intermediate
Antol, 2018	✓	✓	N/A	Unclear	✓	✗	✓	✓	Intermediate
Arnold, 2019	✓	✓	N/A	✓	✓	✗	Unclear	✓	Intermediate
Aziz, 2011	✓	✓	N/A	✓	✓	✗	Unclear	✓	Intermediate
Azizi-Namini, 2019	✓	✓	N/A	✓	✓	✗	Unclear	✓	Intermediate
Bartko, 2019	✓	✓	N/A	✓	✓	✗	Unclear	✓	Intermediate
Bayes-Genis, 2012	✓	✓	N/A	✓	✓	✗	Unclear	✓	Intermediate
Bhatia, 2015	✓	✓	N/A	✓	✓	✗	Unclear	✓	Intermediate
Bobbo, 2017	✓	✓	N/A	✓	✓	✗	Unclear	✓	Intermediate

Bohm, 2015	✓	✓	N/A	✓	✓	✗	Unclear	✓	Intermediate
Boriani, 2012	✓	✓	N/A	✓	✓	✗	Unclear	✓	Intermediate
Boully, 2019	✓	✓	N/A	✓	✓	✗	Unclear	✓	Intermediate
Broch, 2015	✓	✓	N/A	✓	✓	✗	Unclear	✓	Intermediate
Brunner-La Rocca, 2019	✓	✓	N/A	✓	✓	✗	Unclear	✓	Intermediate
Campodonico, 2018	✓	✓	N/A	✓	✓	✗	Unclear	✓	Intermediate
Carlo, 2014	✓	✓	N/A	✓	✓	✗	Unclear	✓	Intermediate
Chang, 2019	✓	✓	N/A	✓	✓	✗	Unclear	✓	Intermediate
Chang, 2019	✓	✓	N/A	✓	✓	✗	Unclear	✓	Intermediate
Choi, 2018	✓	✓	N/A	✓	✓	✗	Unclear	✓	Intermediate
Chopra, 2019	✓	✓	N/A	✓	✓	✗	Unclear	✓	Intermediate
Church, 2015	✓	✓	N/A	✓	✓	✗	Unclear	✓	Intermediate
Coles, 2015	✓	✓	N/A	✓	✓	✗	Unclear	✓	Intermediate
Crespo-Leiro, 2015	✓	✓	N/A	✓	✓	✗	Unclear	✓	Intermediate
Cubbon, 2011	✓	✓	N/A	✓	✓	✗	Unclear	✓	Intermediate
Cubbon, 2019	✓	✓	N/A	✓	✓	✗	Unclear	✓	Intermediate
D Cullington, 2011	✓	✓	N/A	✓	✓	✗	Unclear	✓	Intermediate
de Diego, 2018	✓	✓	N/A	✓	✓	✗	Unclear	✓	Intermediate
De Sutter, 2015	✓	✓	N/A	✓	✓	✗	Unclear	✓	Intermediate
Dec, 2014	✓	✓	N/A	✓	✓	✗	Unclear	✓	Intermediate
Desai, 2010	✓	✓	N/A	✓	✓	✗	Unclear	✓	Intermediate
Desta, 2016	✓	✓	N/A	✓	✓	✗	Unclear	✓	Intermediate
Dev, 2015	✓	✓	N/A	✓	✓	✗	Unclear	✓	Intermediate
DeVore, 2016	✓	✓	N/A	✓	✓	✗	Unclear	✓	Intermediate
DeVore, 2016	✓	✓	N/A	✓	✓	✗	Unclear	✓	Intermediate
DeWolfe, 2010	✓	✓	N/A	✓	✓	✗	Unclear	✓	Intermediate

Diamant, 2019	✓	✓	N/A	✓	✓	✗	Unclear	✓	Intermediate
DiDomenico, 2014	✓	✓	N/A	✓	✓	✗	Unclear	✓	Intermediate
Dierckx, 2015	✓	✓	N/A	✓	✓	✗	Unclear	✓	Intermediate
Fleming, 2016	✓	✓	N/A	Unclear	✓	✗	✓	✓	Intermediate
Flu, 2010	✓	✓	N/A	✓	✓	✗	Unclear	✓	Intermediate
Fontaine, 2016	✓	✓	N/A	✓	✓	✗	Unclear	✓	Intermediate
Fontanive, 2013	✓	✓	N/A	✓	✓	✗	Unclear	✓	Intermediate
Francia, 2015	✓	✓	N/A	✓	✓	✗	Unclear	✓	Intermediate
Franco Pelaez, 2016	✓	✓	N/A	✓	✓	✗	Unclear	✓	Intermediate
Franco, 2014	✓	✓	N/A	✓	✓	✗	Unclear	✓	Intermediate
Franco, 2015	✓	✓	N/A	✓	✓	✗	Unclear	✓	Intermediate
Franco, 2019	✓	✓	N/A	✓	✓	✗	Unclear	✓	Intermediate
Frankenstein, 2011	✓	✓	N/A	✓	✓	✗	Unclear	✓	Intermediate
Frohlich, 2016	✓	✓	N/A	✓	✓	✗	Unclear	✓	Intermediate
Frohlich, 2017	✓	✓	N/A	✓	✓	✗	Unclear	✓	Intermediate
Frohlich, 2018	✓	✓	N/A	✓	✓	✗	Unclear	✓	Intermediate
Fu, 2017	✓	✓	N/A	✓	✓	✗	Unclear	✓	Intermediate
Gasior, 2018	✓	✓	N/A	✓	✓	✗	Unclear	✓	Intermediate
GC Stewart, 2016	✓	✓	N/A	✓	✓	✗	Unclear	✓	Intermediate
Ghimire, 2019	✓	✓	N/A	✓	✓	✗	Unclear	✓	Intermediate
Goland, 2011	✓	✓	N/A	✓	✓	✗	Unclear	✓	Intermediate
Gonzalez-Zuelgaray, 2013	✓	✓	N/A	✓	✓	✗	Unclear	✓	Intermediate
Goto, 2013	✓	✓	N/A	✓	✓	✗	Unclear	✓	Intermediate
Grimm, 2015	✓	✓	N/A	✓	✓	✗	Unclear	✓	Intermediate
Grosu, 2011	✓	✓	N/A	✓	✓	✗	Unclear	✓	Intermediate

Guisado-Espartero, 2018	✓	✓	N/A	✓	✓	✗	Unclear	✓	Intermediate
Gwag, 2018	✓	✓	N/A	✓	✓	✗	Unclear	✓	Intermediate
Han, 2019	✓	✓	N/A	✓	✓	✗	Unclear	✓	Intermediate
Harikrishnan, 2015	✓	✓	N/A	✓	✓	✗	Unclear	✓	Intermediate
Hebert, 2010	✓	✓	N/A	✓	✓	✗	Unclear	✓	Intermediate
Hebert, 2011	✓	✓	N/A	✓	✓	✗	Unclear	✓	Intermediate
Hernandez, 2012	✓	✓	N/A	✓	✓	✗	Unclear	✓	Intermediate
Houard, 2019	✓	✓	N/A	✓	✓	✗	Unclear	✓	Intermediate
Ikeda, 2016	✓	✓	N/A	✓	✓	✗	Unclear	✓	Intermediate
Inampudi, 2014	✓	✓	N/A	✓	✓	✗	Unclear	✓	Intermediate
Ito, 2019	✓	✓	N/A	✓	✓	✗	Unclear	✓	Intermediate
Jankowska, 2014	✓	✓	N/A	✓	✓	✗	Unclear	✓	Intermediate
Kapelios, 2019	✓	✓	N/A	✓	✓	✗	Unclear	✓	Intermediate
Kaplon-Cieslicka, 2014	✓	✓	N/A	Unclear	✓	✗	✓	✓	Intermediate
Kaufmann, 2019	✓	✓	N/A	✓	✓	✗	Unclear	✓	Intermediate
Kessing, 2014	✓	✓	N/A	✓	✓	✗	Unclear	✓	Intermediate
Kisiel, 2018	✓	✓	N/A	✓	✓	✗	Unclear	✓	Intermediate
Komajda, 2016	✓	✓	N/A	✓	✓	✗	Unclear	✓	Intermediate
Komajda, 2017	✓	✓	N/A	✓	✓	✗	Unclear	✓	Intermediate
Komajda, 2019	✓	✓	N/A	✓	✓	✗	Unclear	✓	Intermediate
Koukoui, 2015	✓	✓	N/A	✓	✓	✗	Unclear	✓	Intermediate
Kozdag, 2012	✓	✓	N/A	✓	✓	✗	Unclear	✓	Intermediate
Krishnan, 2015	✓	✓	N/A	✓	✓	✗	Unclear	✓	Intermediate
Kubota, 2018	✓	✓	N/A	✓	✓	✗	Unclear	✓	Intermediate
Laliberte,2017	✓	✓	N/A	✓	✓	✗	Unclear	✓	Intermediate

Lam, 2017	✓	✓	N/A	✓	✓	✗	Unclear	✓	Intermediate
Lanfear, 2012	✓	✓	N/A	Unclear	✓	✗	✓	✓	Intermediate
Lee, 2013	✓	✓	N/A	✓	✓	✗	Unclear	✓	Intermediate
Li, 2019	✓	✓	N/A	✓	✓	✗	Unclear	✓	Intermediate
Lin, 2016	✓	✓	N/A	✓	✓	✗	Unclear	✓	Intermediate
Lindemberg, 2014	✓	✓	N/A	✓	✓	✗	Unclear	✓	Intermediate
Lopatin, 2018	✓	✓	N/A	✓	✓	✗	Unclear	✓	Intermediate
Lopez-Azor, 2019	✓	✓	N/A	✓	✓	✗	Unclear	✓	Intermediate
Lund, 2018	✓	✓	N/A	✓	✓	✗	Unclear	✓	Intermediate
Luo, 2018	✓	✓	N/A	✓	✓	✗	Unclear	✓	Intermediate
Lupon, 2015	✓	✓	N/A	✓	✓	✗	Unclear	✓	Intermediate
Magana-Serrano, 2011	✓	✓	N/A	✓	✓	✗	Unclear	✓	Intermediate
Maggioni, 2013	✓	✓	N/A	✓	✓	✗	Unclear	✓	Intermediate
Magri, 2014	✓	✓	N/A	✓	✓	✗	Unclear	✓	Intermediate
Magri, 2015	✓	✓	N/A	✓	✓	✗	Unclear	✓	Intermediate
Martens, 2018	✓	✓	N/A	✓	✓	✗	Unclear	✓	Intermediate
Martinez-Selles, 2010	✓	✓	N/A	✓	✓	✗	Unclear	✓	Intermediate
Mathew, 2017	✓	✓	N/A	✓	✓	Unclear	✗	✓	Intermediate
Merlo, 2011	✓	✓	N/A	✓	✓	✗	Unclear	✓	Intermediate
Metra, 2010	✓	✓	N/A	✓	✓	✗	Unclear	✓	Intermediate
Migaj, 2018	✓	✓	N/A	✓	✓	✗	Unclear	✓	Intermediate
Miniati, 2013	✓	✓	N/A	✓	✓	✗	Unclear	✓	Intermediate
Mohamadi, 2012	✓	✓	N/A	✓	✓	✗	Unclear	✓	Intermediate
Mojadidi, 2016	✓	✓	N/A	✓	✓	✗	Unclear	✓	Intermediate
Moreno, 2013	✓	✓	N/A	✓	✓	✗	Unclear	✓	Intermediate

Muller, 2017	✓	✓	N/A	✓	✓	✗	Unclear	✓	Intermediate
Munoz, 2017	✓	✓	N/A	✓	✓	✗	Unclear	✓	Intermediate
Nagatomo, 2017	✓	✓	N/A	✓	✓	✗	Unclear	✓	Intermediate
Ne, 2019	✓	✓	N/A	✓	✓	✗	Unclear	✓	Intermediate
Newton, 2016	✓	✓	N/A	✓	✓	✗	Unclear	✓	Intermediate
Oldenburg, 2016	✓	✓	N/A	✓	✓	✗	Unclear	✓	Intermediate
Olsen, 2014	✓	✓	N/A	✓	✓	✗	Unclear	✓	Intermediate
Paolillo, 2017	✓	✓	N/A	✓	✓	✗	Unclear	✓	Intermediate
Parakh, 2012	✓	✓	N/A	✓	✓	✗	Unclear	✓	Intermediate
Pasternak, 2014	✓	✓	N/A	✓	✓	✗	Unclear	✓	Intermediate
Patel, 2016	✓	✓	N/A	✓	✓	✗	Unclear	✓	Intermediate
Peng, 2018	✓	✓	N/A	✓	✓	✗	Unclear	✓	Intermediate
Perez-Rodon, 2018	✓	✓	N/A	✓	✓	✗	Unclear	✓	Intermediate
Pfister, 2011	✓	✓	N/A	✓	✓	✗	Unclear	✓	Intermediate
Poelzl, 2014	✓	✓	N/A	✓	✓	✗	Unclear	✓	Intermediate
Pons, 2010	✓	✓	N/A	✓	✓	✗	Unclear	✓	Intermediate
Qamer, 2019	✓	✓	N/A	✓	✓	✗	Unclear	✓	Intermediate
RafaÅ, Dankowski	✓	✓	N/A	✓	✓	✗	Unclear	✓	Intermediate
Reitan, 2015	✓	✓	N/A	Unclear	✓	✗	✓	✓	Intermediate
Rychli, 2011	✓	✓	N/A	✓	✓	✗	Unclear	✓	Intermediate
Sanam, 2016	✓	✓	N/A	✓	✓	Unclear	Unclear	✓	Intermediate
Santini, 2011	✓	✓	N/A	✓	✓	✗	Unclear	✓	Intermediate
Savarese, 2015	✓	✓	N/A	Unclear	✓	✓	Unclear	✓	Intermediate
Schneider, 2014	✓	✓	N/A	✓	✓	✗	Unclear	✓	Intermediate
Scrutinio, 2014	✓	✓	N/A	✓	✓	✗	Unclear	✓	Intermediate
Senni, 2014	✓	✓	N/A	✓	✓	✗	Unclear	✓	Intermediate

Shelton, 2010	✓	✓	N/A	✓	✓	✗	Unclear	✓	Intermediate
Shen, 2013	✓	✓	N/A	✓	✓	✗	Unclear	✓	Intermediate
Shih-Hung Hsiao	✓	✓	N/A	✓	✓	✗	Unclear	✓	Intermediate
Shih-Hung Hsiao	✓	✓	N/A	✓	✓	✗	Unclear	✓	Intermediate
Shreibati, 2016	✓	✓	N/A	✓	✓	✗	Unclear	✓	Intermediate
Siennicka, 2016	✓	✓	N/A	✓	✓	✗	Unclear	✓	Intermediate
Simioniuc, 2016	✓	✓	N/A	✓	✓	✗	Unclear	✓	Intermediate
Simopoulos, 2015	✓	✓	N/A	✓	✓	✗	Unclear	✓	Intermediate
Stabile, 2018	✓	✓	N/A	✓	✓	✗	Unclear	✓	Intermediate
Stolfo, 2015	✓	✓	N/A	✓	✓	✗	Unclear	✓	Intermediate
Szymanski, 2011	✓	✓	N/A	✓	✓	✗	Unclear	✓	Intermediate
Takada, 2014	✓	✓	N/A	✓	✓	✗	Unclear	✓	Intermediate
Tang, 2013	✓	✓	N/A	✓	✓	✗	Unclear	✓	Intermediate
Taylor, 2012	✓	✓	N/A	✓	✓	Unclear	Unclear	✓	Intermediate
Tokatli, 2015	✓	✓	N/A	✓	✓	✗	Unclear	✓	Intermediate
Toste, 2011	✓	✓	N/A	✓	✓	✗	Unclear	✓	Intermediate
Tran, 2018	✓	✓	N/A	✓	✓	✗	Unclear	✓	Intermediate
Tromp, 2018	✓	✓	N/A	✓	✓	✗	Unclear	✓	Intermediate
Tsuchihashi-Makaya, 2010	✓	✓	N/A	✓	✓	✗	Unclear	✓	Intermediate
Ujeyl, 2011	✓	✓	N/A	✓	✓	✗	Unclear	✓	Intermediate
Valika, 2018	✓	✓	N/A	✓	✓	✗	Unclear	✓	Intermediate
Vandenberk, 2016	✓	✓	N/A	✓	✓	✗	Unclear	✓	Intermediate
Vicent, 2019	✓	✓	N/A	✓	✓	✗	Unclear	✓	Intermediate
Vicent, 2019	✓	✓	N/A	✓	✓	✗	Unclear	✓	Intermediate
Vicent, 2019	✓	✓	N/A	✓	✓	✗	Unclear	✓	Intermediate
von Scheidt, 2014	✓	✓	N/A	✓	✓	✗	Unclear	✓	Intermediate

Wai, 2012	✓	✓	N/A	✓	✓	✗	Unclear	✓	Intermediate
Walker, 2016	✓	✓	N/A	✓	✓	✗	Unclear	✓	Intermediate
Wang, 2016	✓	✓	N/A	✓	✓	✗	Unclear	✓	Intermediate
Wang, 2018	✓	✓	N/A	✓	✓	✗	Unclear	✓	Intermediate
Wohlfahrt, 2015	✓	✓	N/A	✓	✓	✗	Unclear	✓	Intermediate
Wu, 2013	✓	✓	N/A	✓	✓	✗	Unclear	✓	Intermediate
Wurm, 2017	✓	✓	N/A	✓	✓	✗	Unclear	✓	Intermediate
Xin, 2019	✓	✓	N/A	✓	✓	✗	Unclear	✓	Intermediate
Yeh, 2016	✓	✓	N/A	✓	✓	✗	Unclear	✓	Intermediate
Yoo, 2014	✓	✓	N/A	✓	✓	✗	Unclear	✓	Intermediate
Yoo, 2015	✓	✓	N/A	✓	✓	✗	Unclear	✓	Intermediate
Youn, 2012	✓	✓	N/A	✓	✓	✗	Unclear	✓	Intermediate
Yucel, 2015	✓	✓	N/A	✓	✓	✗	Unclear	✓	Intermediate
Zecchin, 2012	✓	✓	N/A	✓	✓	✗	Unclear	✓	Intermediate
Ziaeian, 2017	✓	✓	N/A	✓	✓	✗	Unclear	✓	Intermediate
Zorlu, 2012	✓	✓	N/A	✓	✓	✗	Unclear	✓	Intermediate
Adlbrecht, 2010	✓	✓	N/A	Unclear	✓	✗	Unclear	✓	High risk
Alison Mudge, 2010	✓	✓	N/A	Unclear	✓	✗	Unclear	✓	High risk
Bakos, 2017	✗	✓	N/A	✓	✓	✗	Unclear	✓	High risk
Balling, 2011	✓	✓	N/A	✗	✓	✗	Unclear	✓	High risk
Castelli, 2013	✓	✓	N/A	✗	✓	✗	Unclear	✓	High risk
Dokainish, 2016	✓	✓	N/A	Unclear	✓	✗	Unclear	✓	High risk
Frohlich, 2019	✓	✓	N/A	Unclear	✓	✗	Unclear	✓	High risk
Gentile, 2019	✓	✓	N/A	✓	Unclear	✗	Unclear	✓	High risk
Jackson, 2018	✗	✓	N/A	Unclear	✓	✗	Unclear	✓	High risk
Li, 2015	✓	✓	N/A	Unclear	✓	✗	Unclear	✓	High risk

Mastenbroek, 2016	✓	✓	N/A	Unclear	✓	✗	Unclear	✓	High risk
Mefford, 2019	✓	✓	N/A	Unclear	✓	✗	Unclear	✓	High risk
Neuhold, 2010	✓	✓	N/A	Unclear	✓	✗	Unclear	✓	High risk
Shiga, 2019	✓	✓	N/A	✓	✗	✗	Unclear	✓	High risk
Vicent, 2018	✓	✓	N/A	✗	✓	✗	Unclear	✓	High risk
Zhang, 2017	✓	✓	N/A	Unclear	✓	✗	✗	✓	High risk

N/A: Not applicable

Based on the responses to each of the eight questions, studies were graded on a scale of 1–8 and divided into high (grade <5), intermediate (grade=5), and low (grade >5) risk of bias.

Table S15: Bivariate and multivariate sensitivity analyses for prevalence of use of beta-blockers

		**Estimate**	**95% CI**	**R-square**	**I^2**
**Bivariate models**	Study year	0.016	(0.011, 0.022)	11.71%	99.0%
	Hypertension	0.001	(-0.000, 0.003)	0.88%	99.1%
	IHD/CAD	0.002	(0.000, 0.003)	1.70%	98.9%
	Age	-0.001	(-0.004, 0.002)	0.0%	98.9%
	Country-income level	-0.126	(-0.183, -0.069)	6.09%	98.9%
	Sex	-0.003	(-0.005,-0.001)	3.06%	98.9%
**Multivariate model**	Study year	0.016	(0.010, 0.023)		
	Hypertension	-0.000	(-0.002, 0.002)		
	IHD/CAD	0.002	(0.001, 0.004)		
	Age	-0.007	(-0.011, -0.002)		
	Country-income level	-0.214	(-0.287,-0.142)		
	Sex	-0.002	(-0.005, -0.000)	33.94%	98.53%

Prevalence of beta blocker use is the dependent variable.
IHD/CAD: Ischemic Heart Disease/Coronary Artery Disease

Table S16: Bivariate and multivariate sensitivity analyses for prevalence of use of RAS inhibitors

		Estimate	95% CI	R-square	I^2
**Bivariate model**	Study year	0.003	(-0.003, 0.008)	0.00%	99.1%
	Hypertension	-0.001	(-0.002, 0.000)	0.23%	99.3%
	IHD/CAD	0.001	(-0.001, 0.002)	0.00%	99.0%
	Age	-0.003	(-0.006, -0.000)	1.29%	99.0%
	Country-income level	-0.079	(-0.135,-0.022)	2.36%	99.1%
	Sex	-0.004	(-0.005,-0.002)	5.69%	99.0%
**Multivariate model**	Study year	0.002	(-0.005, 0.009)		
	Hypertension	-0.001	(0.003, 0.000)		
	IHD/CAD	-0.000	(-0.002, 0.002)		
	Age	-0.002	(-0.007, 0.003)		
	Country-income level	-0.123	(-0.199,-0.047)		
	Sex	-0.004	(-0.006, -0.001)	13.22%	99.0%

Prevalence of RAS inhibitor use is the dependent variable.
IHD/CAD: Ischemic Heart Disease/Coronary Artery Disease

Figure S1: Bivariate scatter plot with meta-regression line y-axis= effect, X-axis = year of time publication

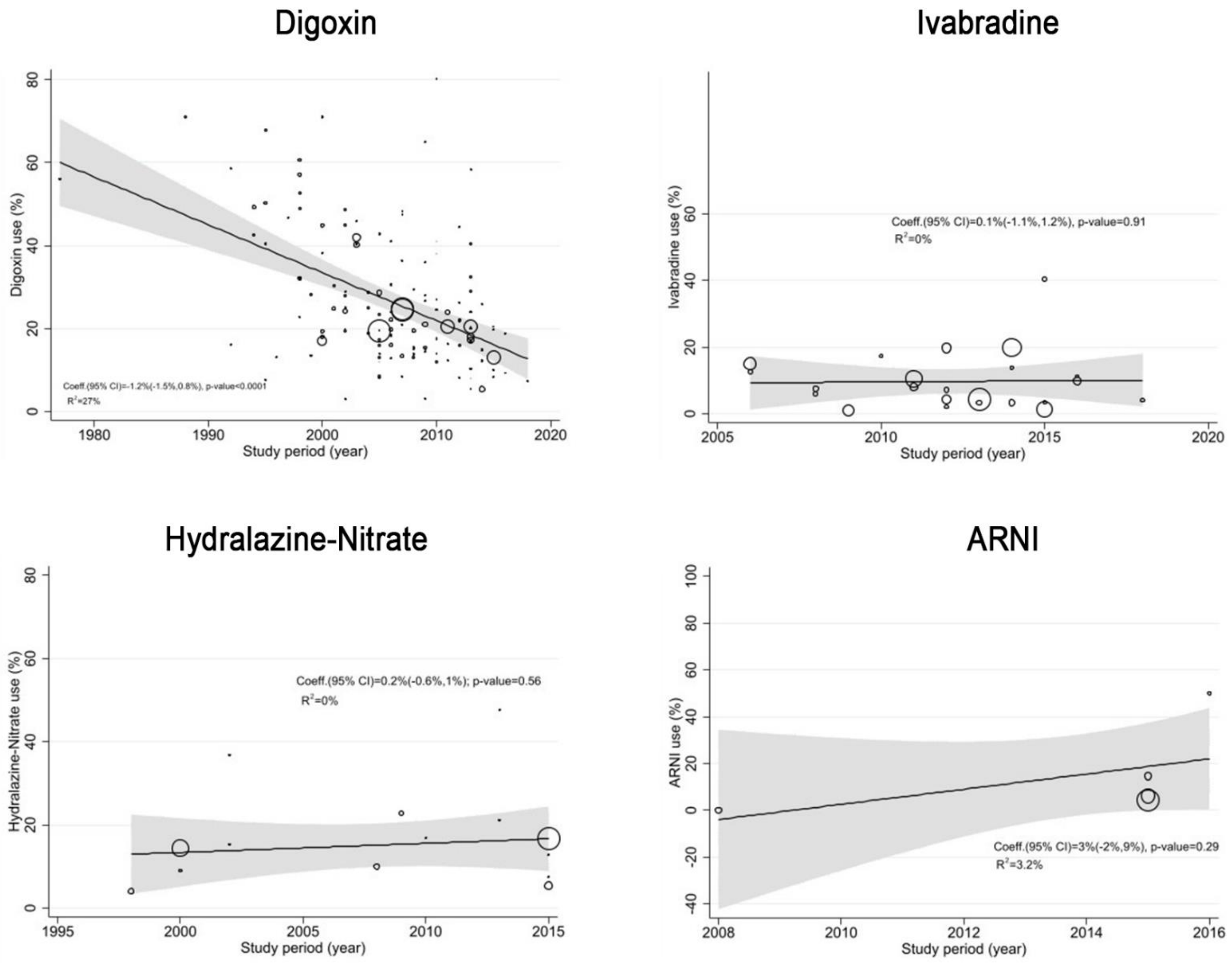

Supplement: Supplementary File. — Tables S1–Table S16 and Figure S1. [file gh-19-1-1355-s1.pdf]
